# Index of the human papillomavirus (HPV) vaccine industry clinical study programmes and non-industry funded studies: a necessary basis to address reporting bias in a systematic review

**DOI:** 10.1186/s13643-018-0675-z

**Published:** 2018-01-18

**Authors:** Lars Jørgensen, Peter C. Gøtzsche, Tom Jefferson

**Affiliations:** Nordic Cochrane Centre, Rigshospitalet 7811, Blegdamsvej 9, 2100 Copenhagen, Denmark

**Keywords:** Human papillomavirus vaccine, Index, Study programme, Clinical study reports, Reporting bias

## Abstract

**Background:**

Unabridged access to drug industry and regulatory trial registers and data reduces reporting bias in systematic reviews and may provide a complete index of a drug’s clinical study programme. Currently, there is no public index of the human papillomavirus (HPV) vaccine industry study programmes or a public index of non-industry funded studies.

**Methods:**

By cross-verification via study programme enquiries to the HPV vaccine manufacturers and regulators and searches of trial registers and journal publication databases, we indexed clinical HPV vaccine studies as a basis to address reporting bias in a systematic review of clinical study reports.

**Results:**

We indexed 206 clinical studies: 145 industry and 61 non-industry funded studies. One of the four HPV vaccine manufacturers (GlaxoSmithKline) provided information on its study programme. Most studies were cross-verified from two or more sources (160/206, 78%) and listed on regulatory or industry trial registers or journal publication databases (195/206, 95%)—in particular, on ClinicalTrials.gov (176/195, 90%). However, study results were only posted for about half of the completed studies on ClinicalTrials.gov (71/147, 48%). Two thirds of the industry studies had a study programme ID, manufacturer specific ID, and national clinical trial (NCT) ID (91/145, 63%). Journal publications were available in journal publication databases (the Cochrane Collaboration’s Central Register of Controlled Trials, Google Scholar and PubMed) for two thirds of the completed studies (92/149, 62%).

**Conclusion:**

We believe we came close to indexing complete HPV vaccine study programmes, but only one of the four manufacturers provided information for our index and a fifth of the index could not be cross-verified. However, we indexed larger study programmes than those listed by major regulators (i.e., the EMA and FDA that based their HPV vaccine approvals on only half of the available trials). To reduce reporting bias in systematic reviews, we advocate the registration and publication of all studies and data in the public domain.

**Electronic supplementary material:**

The online version of this article (10.1186/s13643-018-0675-z) contains supplementary material, which is available to authorized users.

## Background

Healthcare guidelines often rely on drug manufacturers’ studies, regulators’ assessments and independent researchers’ systematic reviews of these studies. Drug manufacturers usually conduct study programmes in agreement with the drug regulators’ pre-approval requirements. However, more than half of all studies are never published [1, 2] and the published studies’ intervention effects are often exaggerated in comparison to the unpublished studies [3–5]. This introduces reporting bias that undermines the validity of systematic reviews. To address reporting bias in systematic reviews, it is necessary to use industry and regulatory trial registers and trial data—in particular, the drug manufacturers’ complete study programmes with their corresponding clinical study reports.

A clinical study report of over a thousand pages in length may be condensed into a ten-page journal publication, i.e., a compression factor of 100 [6, 7]. The criteria used to select the resulting fraction of available data in journal publications are unknown and journal publications are often misleading, especially in relation to the reporting of harms of drug interventions [7–14]. Consequently, some researchers now rely on study programmes and clinical study reports as their primary or only source of information for their systematic reviews [7, 13, 15]. The first systematic review of influenza antivirals showed the feasibility of this approach [15]. The review demonstrated the importance of using clinical study reports and the shortcomings of relying only on journal publication database searches (for example, in PubMed). Although the use of clinical study reports reduces the risk of reporting bias, clinical study reports themselves may still be subject to significant reporting bias when compared to their underlying data [6, 12, 16].

Currently, there is no easily accessible source to access study programmes. However, drug manufacturers’ common technical documents (CTDs) contain lists of the studies in a study programme that support marketing authorization applications (MAAs) to regulatory authorities (such as the European Medicines Agency, EMA, or the Food and Drug Administration, FDA). Since 2010 and 2015, it has been possible for researchers to obtain common technical documents and marketing authorization applications from the EMA via policies 0043 and 0070, respectively.

Nonetheless, accessing study programmes and unpublished studies and obtaining clinical study reports from the industry and regulators can prove difficult [13, 17–20]. Therefore, it is not surprising that only a minority of systematic reviews include unpublished studies (10–20%) [21, 22] or searches of trial registers (10–50%) [23–26]. Searches of trial registers may lead to the identification of additional eligible studies in about 20–60% of systematic reviews [23–26]. To identify unpublished studies and address reporting bias, we present here a method for indexing the study programmes of the human papillomavirus (HPV) vaccines as a basis for a systematic review of clinical study reports (see our protocol at PROSPERO: CRD42017056093 [27]).

## Methods

Our study involved indexing the HPV vaccine industry study programmes and non-industry funded clinical studies using a six-step process that focuses on identifying unpublished studies (see Additional file 1 for a detailed description of steps 1 to 6 and Additional file 2 for the e-mail correspondence with the HPV vaccine manufacturers in step 6).

In step 1, we corresponded with the EMA and obtained a list of their holdings of clinical study reports and Module 2.5 of the common technical document (CTD) for one HPV vaccine (Gardasil 9) that listed all studies in the Gardasil 9 study programme. This gave us a basic study list with industry study identifiers that usually consist of a prefix that identifies the HPV vaccine being tested (for example, HPV-xxx for Cervarix and V50x-xxx for Gardasil studies). In mid-2017, we were granted access to EMA’s holdings of Modules 2.5 of two other HPV vaccines (Cervarix and Gardasil), but we have not received the modules yet.

In step 2, we expanded the basic list by searching 45 trial registers: eight industry trial registers (where the manufacturers had been involved or possibly involved in one or more HPV vaccine studies), 32 international and regional trial registers chosen according to their level of impact (e.g., https://clinicaltrials.gov and http://apps.who.int/trialsearch/ are used globally and were considered high impact) or where one or more HPV vaccine studies had been conducted (e.g., we searched the Chinese Clinical Trial Registry: http://www.chictr.org.cn/index.aspx, since several HPV vaccine studies had been conducted in China) and five regulatory registers (where the regulators had been involved or possibly involved with the assessment or approval of one or more HPV vaccines, e.g., EMA and FDA).

In step 3, we searched the HPV vaccines’ regulatory drug approval packages (DAPs) from FDA and the HPV vaccines’ European Public Assessment Reports (EPARs) from EMA to identify studies possibly not listed in the 45 trial registers.

In step 4, we conducted searches of three journal publication databases (the Cochrane Collaboration’s Central Register of Controlled Trials, Google Scholar, and PubMed) to identify studies possibly not listed in the 45 trial registers. We also searched WikiLeaks for HPV vaccine studies (or related information). When possible, we matched indexed studies to their corresponding journal publications.

In step 5, we added studies listed in recent HPV vaccine regulatory and independent reviews to verify the studies’ existence and add any studies that we had not identified.

In step 6, we sent the assembled indexes to the corresponding HPV vaccine manufacturers and requested them to verify the indexed studies’ existence and add any studies that we had not identified. We gave the manufacturers a 1-month deadline to respond and sent a reminder if the manufacturer had not responded within 1 to 2 weeks.

We indexed interventional prospective preventive (not therapeutic) comparative (with two or more intervention arms) HPV vaccine clinical studies (and their follow-up studies) in humans. We classified studies by cross-verification as follows:

1. “Definitely exists” (cross-verification of a study’s existence from two or more sources)
2. “Probably exists” (verification of a study’s existence from one source)
3. “Probably does not exist” (no manufacturer or regulatory verification but with a passing reference identified from another source)

We indexed studies from the first two categories. No language restriction was applied, and Google Translate was used for non-familiar languages. By comparing all gathered study IDs, we deleted duplicate entries.

One author (LJ) conducted steps 1 to 6 of the indexing and extracted data. The steps were conducted from October 2016 to July 2017. A second author (TJ) checked the indexing and data extraction. Any disagreements were solved by discussion or by consulting the third author (PCG). One author (LJ) classified the studies according to the likelihood of their existence (e.g., “definitely exists”) and assessed the degree of manufacturer involvement (i.e., studies that were funded or partly funded by the manufacturers were classified as industry studies). A second author (TJ) checked the classifications.

For each study, one author (LJ) extracted the following study information: type, phase (I-IV), intervention type, completion status (completed or on-going), centre status (single or multicentre), participant characteristics (age, gender and number of participants), programme ID, manufacturer ID, trial register ID, results availability, and modes of identification.

## Results

**Fig. 1:**
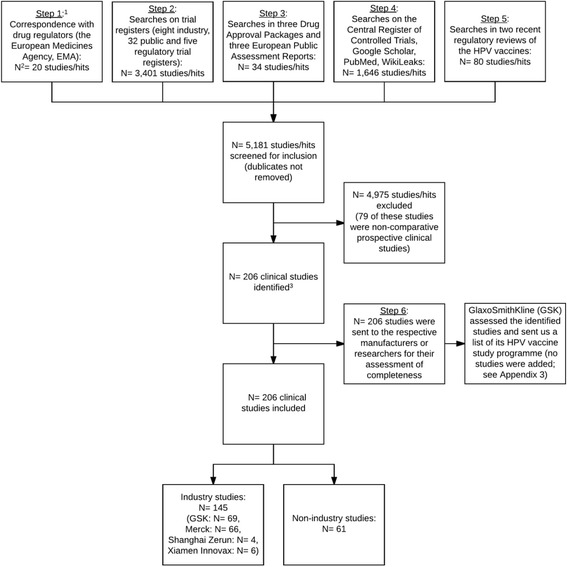
Flowchart of the identification of the HPV vaccines industry study programmes and non-industry funded clinical studies

We excluded 79 non-comparative prospective clinical studies and indexed 206 studies: 145 industry and 61 non-industry funded studies with a total of 623,005 participants. One of the four HPV vaccine manufacturers (GlaxoSmithKline) provided us with an index of 81 GlaxoSmithKline studies and four MedImmune studies (MedImmune and GlaxoSmithKline collaborated in the early development of GlaxoSmithKline’s Cervarix HPV vaccine) (see Fig. 1, Table 1 and Additional files 1, 2, 3; and our study’s PRISMA statement, Additional file 4).

**Table 1:** Characteristics of the HPV vaccine industry study programmes and non-industry funded clinical studies

Study characteristics	Industry HPV vaccine studies				Non-industry funded HPV vaccine studies				*P* value^k^
	Total: *N* = 145	GSK (Cervarix): *N* = 69	Merck (Gardasil and Gardasil 9): *N* = 66	Other^j^: *N* = 10	Total: *N* = 61	Cervarix: *N* = 6	Gardasil and Gardasil 9: *N* = 48	Other: *N* = 7	
Type of study									
Randomized clinical trial	96 (67%)	43 (62%)	45 (68%)	8 (80%)	40 (65%)	4 (67%)	33 (69%)	3 (43%)	1.00
- “Placebo”^a^ comparison	5 of 96 (5%)	0 of 43 (0%)	3 of 45 (7%)	2 of 8 (25%)	12 of 40 (30%)	0 of 4 (0%)	11 of 33 (33%)	1 of 3 (33%)	0.0002
- Adjuvant^b^ comparison	36 of 96 (39%)	15 of 43 (35%)	19 of 45 (42%)	2 of 8 (25%)	0 of 40 (0%)	0 of 4 (0%)	0 of 33 (0%)	0 of 3 (0%)	<0.0001
- Vaccine^c^ comparison	51 of 96 (51%)	28 of 43 (65%)	19 of 45 (42%)	4 of 8 (50%)	24 of 40 (60%)	3 of 4 (75%)	19 of 33 (58%)	2 of 3 (67%)	0.57
- No intervention in control arm	0 of 96 (0%)	0 of 43 (0%)	0 of 45 (0%)	0 of 8 (0%)	3 of 40 (8%)	1 of 4 (25%)	2 of 33 (6%)	0 of 3 (0%)	0.027
- Unclear	4 of 96 (5%)	0 of 43 (0%)	4 of 45 (9%)	0 of 8 (0%)	1 of 40 (2%)	0 of 4 (0%)	1 of 33 (3%)	0 of 3 (0%)	1.00
Follow-up to randomized clinical trial	23 (16%)	18 (26%)	5 (8%)	0 (0%)	3 (5%)	0 (0%)	0 (0%)	3 (43%)	0.037
Non-randomized	24 (16%)	8 (12%)	14 (21%)	2 (20%)	18 (30%)	2 (33%)	15 (31%)	1 (14%)	0.039
Unclear	2 (1%)	0 (0%)	2 (3%)	0 (0%)	0 (0%)	0 (0%)	0 (0%)	0 (0%)	1.00
Phase of study^d^									
I	8 (5%)	2 (3%)	4 (6%)	2 (20%)	6 (10%)	0 (0%)	2 (4%)	4 (57%)	0.36
II	32 (22%)	14 (20%)	14 (21%)	4 (40%)	7 (11%)	0 (0%)	6 (13%)	1 (14%)	0.081
III	79 (55%)	44 (64%)	31 (47%)	4 (40%)	10 (16%)	1 (17%)	9 (19%)	0 (0%)	<0.0001
IV	9 (6%)	4 (6%)	5 (8%)	0 (0%)	22 (37%)	5 (83%)	17 (35%)	0 (0%)	<0.0001
Unclear	17 (12%)	5 (7%)	12 (18%)	0 (0%)	16 (26%)	0 (0%)	14 (29%)	2 (29%)	0.013
Type of HPV vaccine used									
Monovalent	5 (3%)	0 (0%)	5 (8%)	0 (0%)	4 (7%)	0 (0%)	0 (0%)	4 (57%)	0.45
Bivalent (e.g., Cervarix)	76 (52%)	65 (94%)	2 (3%)	9 (90%)	7 (11%)	6 (100%)	0 (0%)	1 (14%)	<0.0001
Quadrivalent (e.g., Gardasil)	43 (30%)	4 (6%)	38 (57%)	1 (10%)	44 (72%)	0 (0%)	44 (92%)	0 (0%)	<0.0001
Octavalent	3 (2%)	0 (0%)	3 (5%)	0 (0%)	0 (0%)	0 (0%)	0 (0%)	0 (0%)	0.56
Ninevalent (e.g., Gardasil 9)	17 (12%)	0 (0%)	17 (25%)	0 (0%)	4 (7%)	0 (0%)	4 (8%)	0 (0%)	0.45
Unclear	1 (1%)	0 (0%)	1 (2%)	0 (0%)	2 (3%)	0 (0%)	0 (0%)	2 (29%)	0.21
Funding									
Industry funded study	128 (88%)	69 (100%)	49 (75%)	10 (100%)	0 (0%)	0 (0%)	0 (0%)	0 (0%)	<0.0001
Industry co-funded study	17 (12%)	0 (0%)	17 (25%)^i^	0 (0%)	0 (0%)	0 (0%)	0 (0%)	0 (0%)	0.004
Non-industry funded study	0 (0%)	0 (0%)	0 (0%)	0 (0%)	61 (100%)	6 (100%)	48 (100%)	7 (100%)	<0.0001
Study completion status									
Completed	110 (76%)	57 (83%)	49 (75%)	4 (40%)	39 (64%)	3 (50%)	29 (60%)	7 (100%)	0.090
- Mean study time in months^e^	36.9 [2; 140]	34.2 [2; 97]	42.1 [8; 140]	15.8 [8; 30]	42.2 [5; 143]	45 [12; 81]	32.6 [6; 66]	56 [5; 143]	NA^l^
On going	27 (19%)	7 (10%)	14 (20%)	6 (60%)	19 (31%)	2 (33%)	17 (36%)	0 (0%)	0.066
Terminated prematurely	5 (3%)	4 (6%)	1 (2%)	0 (0%)	2 (3%)	1 (17%)	1 (2%)	0 (0%)	1.00
Unclear	3 (2%)	1 (1%)	2 (3%)	0 (0%)	1 (2%)	0 (0%)	1 (2%)	0 (0%)	1.00
Study centre status^f^									
Single centre	27 of 106 (25%)	9 of 60 (15%)	11 of 37 (30%)	7 of 9 (78%)	40 of 53 (75%)	6 of 6 (100%)	28 of 41 (68%)	6 of 6 (100%)	<0.0001
Multicentre	79 of 106 (75%)	51 of 60 (85%)	26 of 37 (70%)	2 of 9 (22%)	13 of 53 (25%)	0 of 6 (0%)	13 of 41 (32%)	0 of 6 (0%)	<0.0001
- Mean centres per multicentre study	36.5 [2; 105]	35.3 [2; 135]	39.9 [2; 105]	6.0 [2; 10]	21.5 [2; 134]	NA	21.5 [2; 134]	NA	NA
Unclear	39 (27%)	9 (13%)	29 (44%)	1 (10%)	8 (13%)	0 (0%)	7 (15%)	1 (14%)	0.044
Participants									
Both females and males	22 (16%)	4 (5%)	16 (24%)	2 (20%)	19 (31%)	0 (0%)	14 (29%)	5 (71%)	0.013
Only females	113 (78%)	64 (93%)	41 (62%)	8 (80%)	38 (62%)	6 (100%)	30 (63%)	2 (29%)	0.025
Only males	5 (3%)	1 (2%)	4 (6%)	0 (0%)	4 (7%)	0 (0%)	4 (8%)	0 (0%)	0.45
Unclear	5 (3%)	0 (0%)	5 (8%)	0 (0%)	0 (0%)	0 (0%)	0 (0%)	0 (0%)	0.32
Total number of enrolled participants^g^	522,298	122,323	376,643	23,332	100,707	42,801	47,452	10,454	<0.0001
- Mean participants per study^g^	3602 [2; 189,629]	1773 [2; 34,206]	6726 [24; 189,629]	2592 [90; 12,000]	1767 [12; 24,000]	8560 [200; 24,000]	1054 [12; 20,000]	1493 [45; 10,000]	NA
- Mean participants per multicentre study^f,g^	2388 [20; 34,206]	2073 [20; 34,206]	2692 [67; 14,840]	6450 [900; 12,000]	2745 [75; 20,000]	NA	2745 [75; 20,000]	NA	NA
- Mean participants per centre in multicentre studies^f,g^	66 [2; 1513]	59 [2; 1513]	67 [10; 240]	1075 [450; 1200]	128 [5; 2222]	NA	128 [5; 2222]	NA	NA
Studies with > 1000 participants^g^	51 (35%)	22 (32%)	26 (39%)	3 (30%)	10 (16%)	2 (33%)	7 (15%)	1 (14%)	0.007
Studies with participants under age 18^h^	77 (53%)	23 (33%)	49 (75%)	5 (50%)	28 (46%)	4 (67%)	23 (48%)	1 (14%)	0.36

^a^If a study reported that it used a ‘placebo’ comparator, but the study did not describe the “placebo” (i.e., as saline), we noted the comparator as ‘placebo’

^b^Adjuvant comparisons contained, for example, the aluminum adjuvants used in Cervarix and Gardasil/Gardasil 9, i.e., aluminum hydroxide (Al[OH]_3_) and amorphous aluminum hydroxyphosphate sulfate (AAHS), respectively

^c^Vaccine comparisons included: Adacel, Boostrix, Cervarix (compared with Gardasil or Gardasil 9), Dengvaxia, Engerix, Gardasil (compared with Cervarix or Gardasil 9), Gardasil 9 (compared with Cervarix or Gardasil), Havrix, Infanrix, Menactra, Priorix, Repevax, and Twinrix

^d^If a study was both a phase I and a phase II study, we noted the study as the uppermost phase (i.e., phase II)

^e^105 of the 110 completed industry studies and 33 of the 39 completed non-industry studies had information for mean study time

^f^The exact number of study centers could be assessed in 106 of the 145 industry studies and 53 of the 61 non-industry studies

^g^The number of participants could be assessed in 140 of 145 industry studies and 57 of 61 non-industry studies (studies that were terminated prematurely were not included)

^h^The number of participants under age 18 could be assessed in 140 of the 145 industry studies and 59 of the 61 non-industry studies

^i^10 Merck studies were co-funded with universities, four with hospitals and three with governmental healthcare institutions

^j^Other HPV vaccine manufacturers were Shanghai Zerun Biotechnology Co., Ltd. and Xiamen Innovax Biotech Co., Ltd

^k^P values were calculated for total industry studies vs. total non-industry studies with Fisher’s exact test (http://www.langsrud.com/fisher.htm)

^l^Not applicable

Two thirds of the indexed studies were randomized clinical trials (136/206, 66%) and about half were phase III studies (89/206, 43%). The remaining studies were either follow-ups (23 industry and three non-industry), non-randomized (24 and 18), or of unclear design (two industry studies). Most randomized clinical trials had either another vaccine (for example, the hepatitis A vaccine, Havrix) or the HPV vaccine aluminum adjuvants as comparators (111/136, 82%); only 17 studies used a “placebo” (13%; note that if a study reported that it used a “placebo” comparator, but the study did not describe the “placebo”, i.e., as saline, we noted the study’s comparator as “placebo”). A third of the industry studies and no non-industry study used adjuvant comparisons (36/96 vs. 0/40, *P* < 0.0001; Fisher’s exact test). More industry studies were phase III (79/145 vs. 10/61, *P* < 0.0001) while more non-industry studies used a “placebo” comparison (12/40 vs. 5/96, *P* = 0.0002) and were phase IV studies (22/61 vs. 9/145, *P* < 0.0001) (see Table 1).

GlaxoSmithKline’s study programme primarily used its Cervarix HPV vaccine (65/69, 94%) and included more follow-up studies than the Merck Sharp & Dohme’s study programme (18/69 vs. 5/66, *P* = 0.006) that mainly used its Gardasil or Gardasil 9 HPV vaccines (55/66, 83%, see Table 1).

Most industry studies were solely industry funded (128/145, 88%), but Merck Sharp and Dohme co-funded more studies than GlaxoSmithKline (17/66 vs. 0/69, *P* < 0.0001; ten Merck studies were co-funded with universities, four with hospitals and three with governmental healthcare institutions) (see Table 1).

Most studies were completed (149/206, 72%). In comparison to industry studies, non-industry studies were on average of a longer duration (42.2 vs. 36.9 months), while industry studies were more often multicentre (79/106 vs. 13/53, *P* < 0.0001) (see Table 1).

Most studies included only females (151/206, 73%) and had participants younger than 18 years (105/206, 51%). More non-industry studies included both sexes (19/61 vs. 22/145, *P* = 0.013), but Merck Sharp and Dohme funded more studies on both sexes than GlaxoSmithKline (16/66 vs. 4/69, *P* = 0.003). Industry studies had on average twice as many participants enrolled compared to non-industry studies (3602 vs. 1767). For multicentre studies, the mean number of participants was similar for industry and non-industry studies (2388 vs. 2745), but non-industry studies enrolled twice as many participants per study centre compared to industry studies (128 vs. 66, see Table 1).

Most indexed studies were cross-verified from two or more sources (i.e., “definitely exists”, 160/206, 78%). Merck Sharp and Dohme’s Gardasil and Gardasil 9 programme had more studies with a single verification than GlaxoSmithKline’s Cervarix programme (i.e., “probably exists”, 16/66 vs. 0/69, *P* < 0.0001, see Table 2).

**Table 2:** Study classification, identification and results availability in the HPV vaccine industry study programmes and non-industry funded clinical studies

Study classification, identification and results availability	Industry HPV vaccine studies				Non-industry funded HPV vaccine studies				*P* value^h^
	Total: *N* = 145	GSK (Cervarix): *N* = 69	Merck (Gardasil and Gardasil 9): *N* = 66	Other^g^: *N* = 10	Total: *N* = 61	Cervarix: *N* = 6	Gardasil and Gardasil 9: *N* = 48	Other: *N* = 7	
Study classification^a^									
‘Definitely exists’	127 (88%)	69 (100%)	50 (76%)	8 (80%)	33 (54%)	5 (83%)	28 (58%)	0 (0%)	< 0.0001
‘Probably exists’	18 (12%)	0 (0%)	16 (24%)	2 (20%)	28 (46%)	1 (17%)	20 (42%)	7 (100%)	< 0.0001
Study identification (ID)									
Uses study programme specific^b^ ID	125 (86%)	66 (96%)	49 (74%)	10 (100%)	0 (0%)	0 (0%)	0 (0%)	0 (0%)	< 0.0001
Uses manufacturer specific^c^ ID	98 (68%)	68 (99%)	30 (45%)	0 (0%)	0 (0%)	0 (0%)	0 (0%)	0 (0%)	< 0.0001
Uses both study programme and manufacturer specific IDs	95 (66%)	65 (94%)	30 (45%)	0 (0%)	0 (0%)	0 (0%)	0 (0%)	0 (0%)	< 0.0001
Uses national clinical study (NCT) ID	132 (91%)	64 (93%)	58 (88%)	10 (100%)	44 (72%)	6 (100%)	33 (69%)	5 (71%)	0.0009
Uses study programme, manufacturer specific and NCT IDs	91 (63%)	61 (88%)	30 (45%)	0 (0%)	0 (0%)	0 (0%)	0 (0%)	0 (0%)	< 0.0001
Uses additional or other ID(s)	42 (29%)	26 (38%)	16 (24%)	0 (0%)	51 (84%)	5 (83%)	40 (83%)	6 (86%)	< 0.0001
Study results availability									
Listed in a register or database^d^	138 (95%)	69 (100%)	59 (89%)	10 (100%)	57 (93%)	6 (100%)	45 (94%)	6 (86%)	0.73
Listed on ClinicalTrials.gov	132 (90%)	64 (93%)	58 (88%)	10 (100%)	44 (72%)	6 (100%)	33 (69%)	5 (71%)	0.0009
- Results posted on ClinicalTrials.gov	65 of 132 (44%)	37 of 64 (58%)	28 of 58 (48%)	0 of 10 (0%)	6 of 44 (14%)	0 of 6 (0%)	6 of 33 (18%)	0 of 5 (0%)	0.0002
- Results posted on ClinicalTrials.gov for completed studies	65 of 110 (58%)	37 of 57 (65%)	28 of 49 (57%)	0 of 4 (0%)	6 of 37 (16%)	0 of 3 (0%)	6 of 29 (21%)	0 of 5 (0%)	< 0.0001
Published in a biomedical journal^e^	76 of 110 (69%)	42 of 57 (74%)	34 of 49 (69%)	0 of 4 (0%)	16 of 39 (41%)	3 of 3 (100%)	12 of 29 (41%)	1 of 7 (14%)	0.004
Probably not published in a biomedical journal^e,f^	34 of 110 (31%)	15 of 57 (26%)	15 of 49 (31%)	4 of 4 (100%)	23 of 39 (59%)	0 of 3 (0%)	17 of 29 (59%)	6 of 7 (86%)	0.004

^a^For “definitely exists” studies we demanded cross-verification of a studies existence from two or more sources. For ‘probably exists’ studies we demanded verification of a studies existence from one source

^b^The HPV vaccine manufacturers usually identified their HPV vaccine study programmes with specific identifiers, for example, “HPV-xxx” for Cervarix and “V50x-xxx” Gardasil and Gardasil 9

^c^The HPV vaccine manufacturers usually identified their HPV vaccine studies with manufacturer specific identifiers, for example, GlaxoSmithKline used a six-digit identifier (e.g., 104,896) and Merck Sharp & Dohme used a seven-digit identifier (e.g., 2004_081)

^d^See Additional file 1 for a complete list of the trial registers and databases that we searched

^e^110 of the 145 industry studies and 39 of the 61 non-industry studies were completed and assessed for publication status

^f^”Probably not published” studies were categorized as such if they were not identified as journal publications in the searches we performed (see Methods and Additional files 1 and 3)

^g^Other HPV vaccine manufacturers were Shanghai Zerun Biotechnology Co., Ltd. and Xiamen Innovax Biotech Co., Ltd

^h^P values were calculated for total industry studies vs. total non-industry studies with Fisher’s exact test (http://www.langsrud.com/fisher.htm)

Most industry studies had a study programme specific ID (125/145, 86%), manufacturer specific ID (98/145, 68%) and the US national clinical trial (NCT) ID, which was also used by most non-industry studies (132/145 vs. 44/61, *P* = 0.0009). About two thirds of the industry studies used all three ID types (91/145, 63%, see Table 2).

Most of the included studies were listed in industry, public or regulatory registers or databases (195/206, 95%)—in particular, on ClinicalTrials.gov (176/195, 90%). However, study results were only posted for about half of the completed studies on ClinicalTrials.gov (71/147, 48%), but more completed industry studies posted results on ClinicalTrials.gov compared to non-industry studies (65/110 vs. 6/37, *P* < 0.0001, see Table 2).

We reconciled the index with journal publications, but did not run all-inclusive journal publication database searches as recommended for systematic reviews, since clinical study reports (not journal publications) were our focus. Journal publications were available for two thirds of the completed studies (92/149, 62%, see Table 2).

## Discussion

Our index showed serious deficiencies and variability in the availability of HPV vaccine studies and data. For example, only half of the completed studies listed on ClinicalTrials.gov posted their results. The clinical study reports we obtained via our index process confirmed that the amount of information and data are vastly greater than that in journal publications. For example, the journal publication for one GlaxoSmithKline Cervarix study (HPV-008) is 14 pages long [28] while its publicly available corresponding clinical study report is more than 7000 pages long [29], even though it is a shortened interim report.

Identification of some studies involved a considerable amount of work and a fifth of the index could not be cross-verified. We indexed studies that we would not have been able to index if we only relied on the journal publication databases. For example, one Cervarix randomized clinical trial (HPV-002) was only listed on GlaxoSmithKline’s trial register. The index that GlaxoSmithKline provided us with contained three Cervarix meta-analyses that were only identifiable by their GSK ID (i.e., 205206, 207644, and 205639), and the index did not include three randomized clinical trials (HPV-009 and HPV-016 and the prematurely terminated HPV-078) that were listed on ClinicalTrials.gov. One Cervarix trial (HPV-049) and three Gardasil studies (V501–001, V501–002, and V501–004) were only identified via regulatory registers or correspondence. One Gardasil follow-up study (for V501–005) only had a journal publication listed in PubMed without any manufacturer-specific ID or registration. Four non-industry studies were published in journal publications but were not registered in any of the 45 trial registers. Three non-industry studies were only listed on regional trial registers (Australia: https://www.anzctr.org.au/; Germany: https://www.drks.de/; and India: http://ctri.nic.in/) (see Additional files 2 and 3).

We also indexed more studies than those listed in the holdings of major regulators. For example, EMA conducted a review (of the relation between HPV vaccination and two syndromes: postural orthostatic tachycardia syndrome, POTS and complex regional pain syndrome, CRPS) [30] that EMA believed was based on the manufacturers’ complete HPV vaccine study programmes (“GSK [GlaxoSmithKline] has conducted a review of all available data from clinical studies…with Cervarix”; and “The MAH [Market Authorisation Holder, i.e., Merck Sharp and Dohme] has reviewed data from all clinical studies of the qHPV vaccine [Gardasil]” (30,31)). However, when we compared the manufacturers’ study programmes (submitted to EMA, see Additional file 1) with our index (see Table 1 and Additional file 3), we found that only half (38/79, 48%) of the manufacturers’ randomized clinical trials and follow-ups of Cervarix and Gardasil completed before the submission dates in July 2015 were included (EMA’s review did not assess Gardasil 9) [30, 31]. Similarly, the FDA’s Drug Approval Packages (DAPs) only mentioned half (32/60, 53%) of the randomized clinical trials and follow-ups that were completed before the vaccines’ date of the Drug Approval Packages (Cervarix: 17/36, 47%; Gardasil: 6/11, 54%; and Gardasil 9: 9/13, 69%). We find this very disturbing.

To our knowledge, our study is the first with the aim of indexing a complete study programme. We do not know if the considerable reporting bias we found is generalizable to all drugs and vaccines, but similar industry examples exist for oseltamivir [15], rofecoxib [32], and rosiglitazone where 83% (35/42) of the study programme was unpublished [33, 34]. Indexing is important when there is high risk of reporting bias, which often is the case for industry funded drug trials. Our approach should therefore be considered for systematic reviews of drugs and vaccines. Steps 2 and 4 contributed quantitatively the most to the identification and cross-verification of studies—in particular, searches on ClinicalTrials.gov and the HPV vaccine manufacturers’ trial registers. Searches of regulatory registers and journal publication databases contributed to a lesser extent. Steps 1, 3, 5 and 6 contributed mainly to the verification of some studies (see Additional files 1 and 3).

Our six-step process is reproducible, the step sequence is interchangeable and most steps could be performed simultaneously. For example, we started by corresponding with EMA, since we are familiar with EMA’s handling of study programmes and clinical study reports [27]. This correspondence helped us get started (but EMA response times may prove very slow and EMA often denies data requests (18)). The index took approximately 3 months to assemble. Researchers may save time if they perform the steps simultaneously and focus on steps 2 and 6. For example, researchers could start requesting the drug manufacturers’ study programmes and subsequently make an independent index and compare the two. However, correspondence with manufacturers may prove challenging and slow. Only one of the four HPV vaccine manufacturers (GlaxoSmithKline) provided us with study programme information, which we received 9 months after our initial request. Merck Sharp and Dohme responded to our enquiry, but did not provide study programme information. Shanghai Zerun Biotechnology and Xiamen Innovax Biotech did not respond to our inquiries (Additional files 1, 2, 3, and 5).

Compared to industry studies, non-industry funded studies were registered less often (for example, on ClinicalTrials.gov) and posted less study results. Non-industry researchers are not legally required to register their studies, adhere to industry reporting guidelines (the International Conference on Harmonization of Technical Requirements for Registration of Pharmaceuticals for Human Use, ICH: http://www.ich.org/) or produce clinical study reports unless their results are used to support a drug’s marketing authorization application. This involves a high risk of reporting bias. Therefore, we recommend that non-industry funders require researchers to register their studies and commit to reporting guidelines similar to the ICH.

Finally, although study programme and clinical study report access from the industry and regulators have improved since 2010 [17], access is often slow and inefficient [18]. In May 2014, one of us (TJ) requested the HPV vaccine clinical study reports from EMA. The request was initially declined by EMA because, “disclosure would undermine the protection of commercial interests”. TJ successfully appealed, but EMA has only released 18 incomplete clinical study reports more than 3 years after the initial request (as of 1 July 2017), which is only half of the clinical study reports included in the EMA review (18/38) [30] and a fifth of our indexed randomized clinical industry trials (18/96).

## Conclusion

Authors of systematic reviews may recognize and reduce reporting bias if they adopt our index process of study programmes. We believe we came close to indexing complete HPV vaccine study programmes, but only one of the four HPV vaccine manufacturers provided information for our index and a fifth of the index could not be cross-verified. However, we indexed larger study programmes than those listed by major regulators (i.e., the EMA and FDA that based their HPV vaccine approvals on only half of the available trials). To reduce reporting bias in systematic reviews, we advocate the registration and publication of all studies and data in the public domain and that non-industry studies register and adhere to reporting guidelines similar to the ICH.

## Additional files

Additional file 1: Index of the HPV vaccines clinical studies: Search strategy for identifying the HPV vaccines industry study programmes and non-industry funded clinical studies. (DOC 1294 kb)

Additional file 2: Index of the HPV vaccines clinical studies: Correspondence with the HPV vaccine manufacturers for the assessment of the accuracy of our indexed industry study programmes. (DOC 151 kb)

Additional file 3: Index of the HPV vaccines clinical studies: Indexes of the identified industry study programmes and non-industry funded clinical studies and a list of the identified corresponding journal publications. (DOC 659 kb)

Additional file 4: Prisma 2009 checklist. (DOCX 147 kb)

Additional file 5: Data sharing agreement with GlaxoSmithKline.(PDF 301 kb)

## Abbreviations

CTD: Common technical document
DAP: Drug approval package
EMA: European medicines agency
EPAR: European public assessment report
FDA: Food and Drug Administration
GSK: GlaxoSmithKline plc
HPV: Human papillomavirus
ICH: International conference on harmonization of technical requirements for registration of pharmaceuticals for human use
MAA: Marketing authorization application
MAH: Market authorization holder
Merck: Merck Sharp & Dohme (or Merck & Co., Inc.)
NCT: National clinical trial
