# Supplementary material for: Index of the human papillomavirus (HPV) vaccine industry clinical study programmes and non-industry funded studies: a necessary basis to address reporting bias in a systematic review
Source: Syst Rev. 2018 Jan 18;7:8. doi: 10.1186/s13643-018-0675-z (PMC5774129; doi:10.1186/s13643-018-0675-z)
Supplement: Supplementary file 1 — Index of the HPV vaccines clinical studies: Search strategy for identifying the HPV vaccines industry study programmes and non-industry funded clinical studies. (DOC 1294 kb) [file 13643_2018_675_MOESM1_ESM.doc]

**Additional file 1: Index of the HPV vaccines clinical studies: Search strategy for identifying the HPV vaccine industry study programmes and non-industry funded clinical studies**

The search strategy for the HPV vaccines studies was developed iteratively in six steps. Steps 2 and 4 contributed quantitatively the most to the identification of studies—in particular, searches of ClinicalTrials.gov and the HPV vaccine manufacturers trial registers. Searches of regulatory registers and journal publication databases contributed to a lesser extent. Steps 1, 3, 5 and 6 contributed mainly in the verification of some studies (see Appendix 3).

*- Summary of identified studies/hits with each of the six search steps:*

| **Step** | **Source** | **Number of studies/hits** |
| --- | --- | --- |
| 1 | Correspondence with EMA and inclusion of the HPV vaccines Market Authorisation Applications | 20 |
| 2 | Searches of industry, public and regulatory trial registers | 3,401 |
| 3 | Inclusion of Drug Approval Packages and European Public Assessment Reports from FDA and EMA | 34 |
| 4 | Searches on the Central Register of Controlled Trials (CENTRAL), Google Scholar, PubMed and WikiLeaks | 1,646 |
| 5 | Inclusion of HPV vaccine studies listed on recent regulatory reviews | 80 |
| **Total** |  | **5,181** |
| *6* | *The HPV vaccine manufacturers assessment of the study indexes accuracy:*  *N= studies in the Cervarix index that GlaxoSmithKline provided us with* | *85* |

Step 1: Correspondence with EMA and inclusion of the HPV vaccines’ Market Authorisation Applications

We included studies listed in correspondence with EMA and studies listed on the HPV vaccines Market Authorisation Applications.

*- Summary of identified studies/hits in s*tep 1:

| **Step** | **Source** | **Number of studies/hits** |
| --- | --- | --- |
| 1a | Correspondence with EMA | 13 |
| 1b | The HPV vaccines Market Authorisation Applications | 7 |
| **Total hits** |  | **20** |

*Step 1a: Correspondence with EMA*

From May 2014 we corresponded with and obtained HPV vaccine clinical study reports from EMA. For example, we requested EMA for a list of the completed HPV vaccine studies listed on EMA’s pharmacovigilance plan. The following is an email from EMA dated 18 October 2016 (some of the studies that EMA listed had very opaque titles, e.g., ‘Protocol using the PGRx’ and ‘System Pregnancy Registry’):

“Dear Dr Tom Jefferson, The list of completed studies from the pharmacovigilance plan with the final report submitted for regulatory review is as follows: Cervarix:
Study HPV-008: A phase III, double-blind, randomized, controlled, multicentre study to evaluate the efficacy of GlaxoSmithKline Biologicals’ HPV-16/18 VLP/AS04 vaccine compared to hepatitis A vaccine as control in prevention of persistent HPV-16 or HPV-18 cervical infection and cervical neoplasia, administered intramuscularly according to a 0, 1, 6 month schedule in healthy females 15-25 years of age.
Study HPV-009: A double-blind, controlled, randomized, phase III study of the efficacy of an HPV-16/18 VLP vaccine in the prevention of advanced cervical intraepithelial neoplasia (CIN2, CIN3, adenocarcinoma in situ [AIS] and invasive cervical cancer) associated with HPV-16 or HPV-18 cervical infection in healthy young adult women in Costa Rica
Study HPV-020: A phase I/II, partially-blind, randomized, controlled study to assess the safety and immunogenicity of GlaxoSmithKline Biologicals’ HPV-16/18 L1 VLP AS04 vaccine administered intramuscularly according to a three-dose schedule (0, 1, 6-month) in human immunodeficiency virus (HIV)-infected female subjects aged 18-25 years (South Africa)
EPI-HPV-020: An observational cohort study assessing the risk of spontaneous abortions during the first 23 weeks of gestation in women aged 15 to 25 years exposed to Cervarix, residing in the United Kingdom and reporting their last menstrual period between 30 days before and 45 days after any dose of Cervarix
EPI-HPV-018 (PASS): An observational cohort study assessing the risk of spontaneous abortions during the first 23 weeks of gestation in women aged 15 to 25 years exposed to Cervarix, residing in the United Kingdom and reporting their last menstrual period between 30 days before and 45 days after any dose of Cervarix
EPI-HPV-015 : A post-marketing observational safety study of autoimmune diseases following GlaxoSmithKline (GSK) Biologicals’ HPV-16/18 L1 VLP AS04 vaccine (Cervarix®) vaccination in females aged 9-25 years enrolled in United States health plans 
EPI-HPV-040 (PASS study): (replacing study HPV-015). An observational cohort study to assess the risk of autoimmune diseases in adolescent and young adult women aged 9 to 25 years exposed to Cervarix® in the United Kingdom (using the CPRD GOLD data source in the UK)
Study HPV-015: A phase III, double-blind, randomized, controlled study to evaluate the safety, immunogenicity and efficacy of GlaxoSmithKline Biologicals’ HPV-16/18 L1/AS04 vaccine administered intramuscularly according to a three-dose schedule (0, 1, 6 month) in healthy adult female subjects aged 26 years and above
Study HPV-040: A phase III/IV, community-randomized, controlled study to evaluate the effectiveness of two vaccination strategies using GlaxoSmithKline Biologicals’ HPV-16/18 L1 VLP AS04 vaccine in reducing the prevalence of HPV-16/18 infection when administered intramuscularly according to a 0, 1, 6-month schedule in healthy female and male study participants aged 12 – 15 years.

Gardasil
Long term follow-up study in adolescents (P018-11)
Post-Licensure Safety study in Females (P031)
Protocol using the PGRx System 
Pregnancy Registry
Vaccination Impact in Population Study (P033)

Gardasil 9
No studies from the pharmacovigilance plan have been completed yet. No interim results from studied in the pharmacovigilance plan have been submitted for regulatory review."

*Step 1b: Inclusion of the HPV vaccines Market Authorisation Applications*

We requested the EMA for the Market Authorisation Applications (MAAs) for the HPV vaccines. We obtained the Market Authorisation Applications for Gardasil 9, but we did not obtain the Market Authorisation Applications for Cervarix or Gardasil from EMA. Module 2.5 of the Common Technical Document (CTD) of Gardasil 9 (EMA reference: GARDASIL 9, EMA ASK 21232, first batch release) was sent to us by the EMA on 10 November 2016. The following screenshot is a list from Module 2.5 with the studies that contributed to the safety dataset of Gardasil 9 studies:


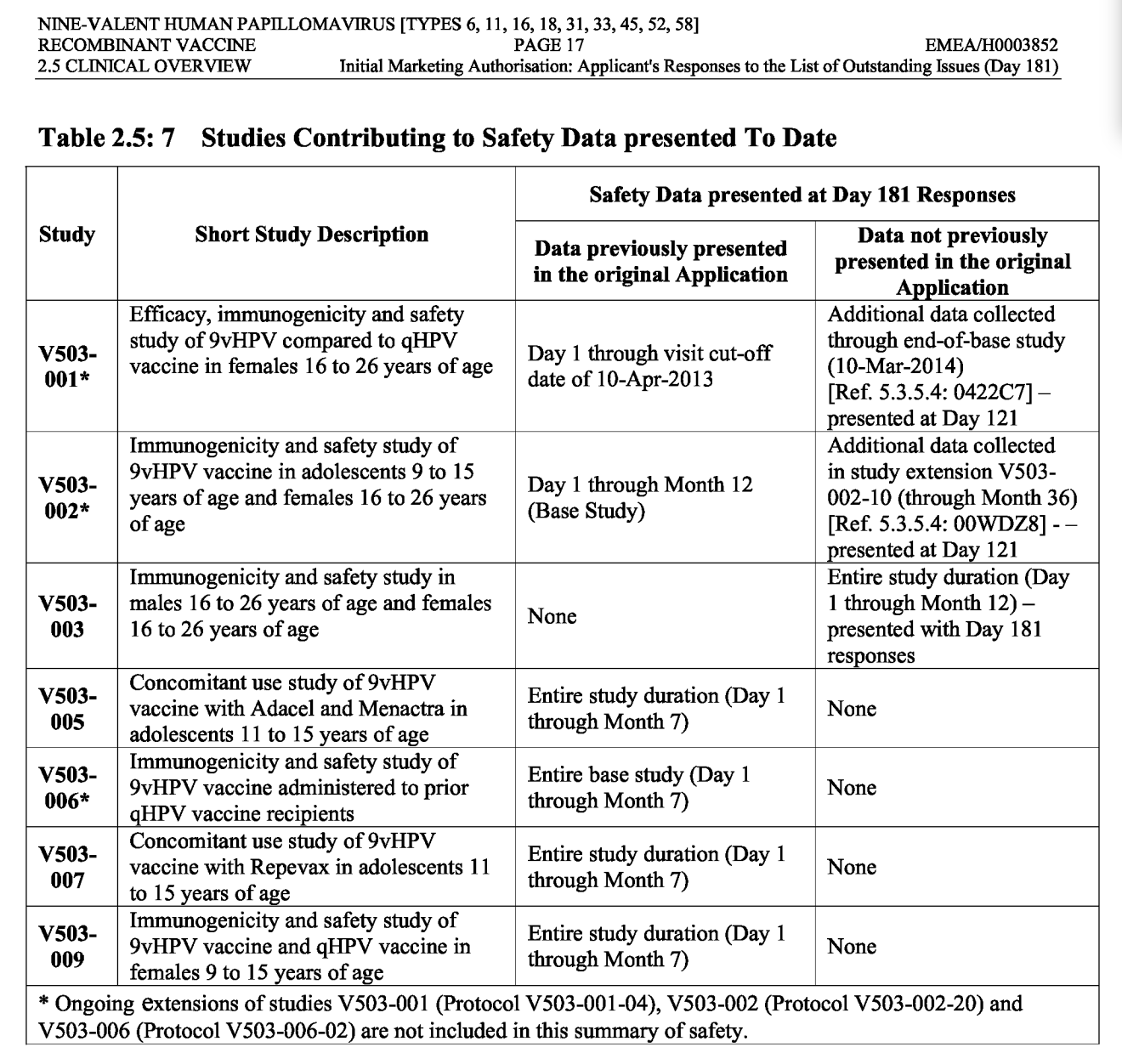


Step 2: Searches on industry, public and regulatory trial registers

We searched industry, public and regulatory trial registers for HPV vaccine studies.

*- Summary of identified studies/hits in s*tep 2:

| **Step** | **Source** | **Number of studies/hits** |
| --- | --- | --- |
| 2a | Industry trial registers | 404 |
| 2b | Public trial registers | 1,672 |
| 2c | Regulatory registers | 1,325 |
| **Total hits** |  | **3,401** |

*Step 2a: Searches on industry trial registers*

We searched an industry trial register if the manufacturer had been involved (or possibly involved) in one or more HPV vaccine studies:

| **Industry register searched** | **Search phrase or term used** | **Date of search** | **Number of hits** |
| --- | --- | --- | --- |
| CSDR: https://clinicalstudydatarequest.com/ | “Human Papillomavirus Types 16 and 18 Vaccine” [this was the only eligible search option] | 7 July 2017 | 57 |
| GlaxoSmithKline: https://gsk-clinicalstudyregister.com/ | "Human papillomavirus vaccine", “HPV vaccine”, “Cervarix”, “Gardasil” | 7 July 2017 | 227 |
| Inovio Pharmaceuticals: <http://www.inovio.com/> | "Human papillomavirus vaccine", “HPV vaccine” | 7 July 2017 | 56 |
| Merck Sharp & Dohme: <http://www.merck.com/clinical-trials/> | "Human papillomavirus vaccine", “HPV vaccine”, “Cervarix”, “Gardasil” | 7 July 2017 | 64 |
| Sanofi Pasteur: <http://www.sanofipasteur.com/> | "Human papillomavirus vaccine", “HPV vaccine”, “Cervarix”, “Gardasil” | 7 July 2017 | 0 |
| Serum Institute of India Pvt. Ltd: <http://www.seruminstitute.com/> | Not applicable [no search option on this website. We did not contact the manufacturer as no identified HPV vaccine study was funded by the manufacturer] | 7 July 2017 | Unknown |
| Shanghai Zerun Biotechnology Co., Ltd: <http://www.zerunbio.com/> | Not applicable [no search option on this website. We contacted this manufacturer as the manufacturer had funded several HPV vaccine studies] | 7 July 2017 | Unknown |
| Xiamen Innovax Biotech Co., Ltd: <http://www.innovax.cn/> | "Human papillomavirus vaccine", “HPV vaccine” | 7 July 2017 | 0 |
| **Total** |  |  | **404** |

*Step 2b: Searches on public trial registers*

We searched the following 32 international and regional trial registers for HPV vaccine studies chosen according to their level of impact (e.g., [https://clinicaltrials.gov](https://clinicaltrials.gov/) and <http://apps.who.int/trialsearch/> are used internationally and were considered high impact) and where one or more HPV vaccine studies had been conducted (for example, we searched the Chinese Clinical Trial Registry: <http://www.chictr.org.cn/index.aspx>, since several HPV vaccine studies had been conducted in China):

| **International trial registers** | | | |
| --- | --- | --- | --- |
| **Trial register** | **Search phrase or term used** | **Date of search** | **Number of hits** |
| CenterWatch: <http://www.centerwatch.com/> | Human Papilloma Virus (HPV) Clinical Trials [this was the only eligible search option] | 9 July 2017 | 5 |
| Clinical Trial Facts: <http://www.clinicaltrialfacts.com/> | "Human papillomavirus vaccine", “HPV vaccine”, “Cervarix”, “Gardasil” | 9 July 2017 | 87 |
| Clinical Trials Feeds: <http://clinicaltrialsfeeds.org/> | Not applicable [no eligible search option] | 9 July 2017 | 0 |
| ClinicalTrials.gov: <https://clinicaltrials.gov/home> | “Bivalent HPV OR quadrivalent HPV OR HPV vaccine OR human papillomavirus vaccine OR Cervarix OR Gardasil” | 9 July 2017 | 421 |
| NHS Choices: <http://www.nhs.uk/Conditions/Clinical-trials/Pages/clinical-trial.aspx> | "HPV vaccination" [only eligible search option] | 9 July 2017 | 102 |
| Patients Like Me: <https://www.patientslikeme.com/clinical_trials> | "Condition: Papillomavirus [intervention type: biologic]” | 9 July 2017 | 118 |
| The ISRCTN Registry: <http://www.isrctn.com/> | "Human papillomavirus vaccine", “HPV vaccine”, “Cervarix”, “Gardasil” | 9 July 2017 | 18 |
| Trials Central: <http://www.trialscentral.org/> | Not applicable [no search option on this website] | 9 July 2017 | 0 |
| VirtualTrials.com: <https://virtualtrials.com/clinical_trials_finder.cfm> | Not applicable [no eligible search option] | 9 July 2017 | 0 |
| WHO International Clinical Trials Registry Platform (ICTRP): <http://apps.who.int/trialsearch/> | Advanced search: Intervention: “Human papillomavirus vaccine OR HPV vaccine OR Cervarix OR Gardasil” | 9 July 2017 | 531 |
| **African trial registers** | | | |
| **Trial register** | **Search phrase or term used** | **Date of search** | **Number of hits** |
| Pan African Clinical Trials Registry: <http://www.pactr.org/> | "Human papillomavirus vaccine", “HPV vaccine”, “Cervarix”, “Gardasil” | 11 July 2017 | 6 |
| South African National Clinical Trial Register: <http://www.sanctr.gov.za/> | Not applicable [no eligible search options] | 11 July 2017 | 0 |
| **Asian trial registers** | | | |
| **Trial register** | **Search phrase or term used** | **Date of search** | **Number of hits** |
| Chinese Clinical Trial Registry: <http://www.chictr.org.cn/index.aspx> | Not applicable [this website did not respond to any searches] | 11 July 2017 | Unknown |
| India’s Clinical Trials Registry: <http://ctri.nic.in/Clinicaltrials/login.php> | “Cervarix” OR “Gardasil” OR “HPV vaccine” AND "Intervention and comparator agent" | 11 July 2017 | 8 |
| Japanese Clinical Trial Registry: <http://rctportal.niph.go.jp/en/> | "Human papillomavirus vaccine", “HPV vaccine”, “Cervarix”, “Gardasil” | 11 July 2017 | 7 |
| Korean National Clinical Research Coordination Centre: <http://ncc.re.kr/> | Not applicable [this website is not accessible from a non-Korean server] | 11 July 2017 | Unknown |
| Sri Lankan Clinical Trials Registry: <http://www.slctr.lk/> | "Human papillomavirus vaccine", “HPV vaccine”, “Cervarix”, “Gardasil” | 11 July 2017 | 0 |
| **European and Middle Eastern trial registers** | | | |
| **Trial register** | **Search phrase or term used** | **Date of search** | **Number of hits** |
| European Clinical Trials Register: <https://www.clinicaltrialsregister.eu/index.html> | "Human papillomavirus vaccine", “HPV vaccine”, “Cervarix”, “Gardasil” | 11 July 2017 | 148 |
| German Clinical Trials Register: <https://www.drks.de/drks_web/> | "Human papillomavirus vaccine", “HPV vaccine”, “Cervarix”, “Gardasil” | 11 July 2017 | 169 |
| Iranian Registry of Clinical Trials: <http://www.irct.ir/searchen.php> | "Human papillomavirus vaccine", “HPV vaccine”, “Cervarix”, “Gardasil” | 11 July 2017 | 0 |
| Nederlands Trial Register: <http://www.trialregister.nl/trialreg/index.asp> | "Human papillomavirus vaccine", “HPV vaccine”, “Cervarix”, “Gardasil” | 11 July 2017 | 0 |
| United Kingdom Cancer Research: <http://www.cancerresearchuk.org/about-cancer/find-a-clinical-trial> | "Human papillomavirus vaccine", “HPV vaccine”, “Cervarix”, “Gardasil” | 11 July 2017 | 0 |
| United Kingdom’s Clinical Trials Gateway: <https://www.ukctg.nihr.ac.uk/> | "The system will search for clinical trials from two sources: the ClinicalTrials.gov register; and the ISRCTN." [ClinicalTrials and ISRCTN were already included] | 11 July 2017 | Not applicable |
| **North American trial registers** | | | |
| **Trial register** | **Search phrase or term used** | **Date of search** | **Number of hits** |
| ClinicalTrials.com: <http://www.clinicaltrials.com/> | "Human papillomavirus vaccine", “HPV vaccine”, “Cervarix”, “Gardasil” | 11 July 2017 | 0 |
| Health Canada: <https://www.canada.ca/en/health-canada/> | "Human papillomavirus vaccine", “HPV vaccine”, “Cervarix”, “Gardasil” | 11 July 2017 | 3 |
| Trial X: <http://trialx.com/> | "Human papillomavirus vaccine", “HPV vaccine”, “Cervarix”, “Gardasil” | 11 July 2017 | 13 |
| **Oceania trial registers** | | | |
| **Trial register** | **Search phrase or term used** | **Date of search** | **Number of hits** |
| Australian Clinical Trials: <https://www.australianclinicaltrials.gov.au/> | "Human papillomavirus vaccine", “HPV vaccine”, “Cervarix”, “Gardasil” | 11 July 2017 | 4 |
| Australian and New Zealand Clinical Trials Registry: <http://www.anzctr.org.au/default.aspx> | "Human papillomavirus vaccine", “HPV vaccine”, “Cervarix”, “Gardasil” | 11 July 2017 | 32 |
| New Zealand Clinical Trials Portal: <http://clinicaltrials.health.nz/> | "Human papillomavirus vaccine", “HPV vaccine”, “Cervarix”, “Gardasil” | 11 July 2017 | 0 |
| **South American trial registers** | | | |
| **Trial register** | **Search phrase or term used** | **Date of search** | **Number of hits** |
| Brazilian Clinical Trials Registry: <http://www.ensaiosclinicos.gov.br/> | "Human papillomavirus vaccine", “HPV vaccine”, “Cervarix”, “Gardasil” | 11 July 2017 | 0 |
| Cuban Public Registry of Clinical Trials: <http://registroclinico.sld.cu/en/home> | "Human papillomavirus vaccine", “HPV vaccine”, “Cervarix”, “Gardasil” | 11 July 2017 | 0 |
| South American (SciELO) Public Health Trial Register: <http://www.scielosp.org/scielo.php?lng=en> | "Human papillomavirus vaccine", “HPV vaccine”, “Cervarix”, “Gardasil” | 11 July 2017 | 0 |
| **Total hits** |  |  | **1,672** |

*Step 2c: Searches on regulatory registers*

We searched a regulatory register if the regulator had been involved (or possibly involved) in the assessment or approval of one or more of the HPV vaccines:

| **Register searched** | **Search phrase or term used** | **Date of search** | **Number of hits** |
| --- | --- | --- | --- |
| Chinese Food and Drug Administration: <http://eng.sfda.gov.cn/WS03/CL0755/> | "Human papillomavirus vaccine", “HPV vaccine”, “Cervarix”, “Gardasil” | 13 July 2017 | 52 |
| European Medicines Agency: <http://www.ema.europa.eu/ema/> | “HPV vaccine OR Cervarix OR Gardasil AND study OR trial" | 13 July 2017 | 408 |
| India’s Central Drugs Control Organization: <http://cdsco.nic.in/forms/Default.aspx> | "Human papillomavirus vaccine", “HPV vaccine”, “Cervarix”, “Gardasil” | 13 July 2017 | 1 |
| United Kingdom Medicines & Healthcare products Regulatory Agency: <https://www.gov.uk/government/organisations/medicines-and-healthcare-products-regulatory-agency> | "Human papillomavirus vaccine OR HPV vaccine OR Cervarix OR Gardasil” | 13 July 2017 | 274 |
| United States Food and Drug Administration: <http://www.fda.gov/> | "Human papillomavirus vaccine", “HPV vaccine”, “Cervarix”, “Gardasil” | 13 July 2017 | 590 |
| **Total** |  |  | **1,325** |

Step 3: Drug Approval Packages and European Public Assessment Reports from FDA and EMA

We searched the HPV vaccines’ Drug Approval Packages and European Public Assessment Reports from FDA and EMA for HPV vaccine studies:

| **The FDA’s Drug Approval Packages** | | | |
| --- | --- | --- | --- |
| **HPV vaccine** | **Source** | **Date of search** | **Number of studies** |
| Cervarix | https://www.fda.gov/BiologicsBloodVaccines/Vaccines/ApprovedProducts/ucm186957.htm | 14 July 2017 | 17 |
| Gardasil | <https://www.fda.gov/biologicsbloodvaccines/vaccines/approvedproducts/ucm094042> | 14 July 2017 | 7 |
| Gardasil 9 | <https://www.fda.gov/biologicsbloodvaccines/vaccines/approvedproducts/ucm426445.htm> | 14 July 2017 | 10 |
| **The EMA’s European Public Assessment Reports** | | | |
| **HPV vaccine** | **Source** | **Date of search** | **Number of studies** |
| Cervarix | <http://www.ema.europa.eu/docs/en_GB/document_library/EPAR_-_Summary_for_the_public/human/000721/WC500024634.pdf> | 14 July 2017 | Unknown* |
| Gardasil | <http://www.ema.europa.eu/docs/en_GB/document_library/EPAR_-_Summary_for_the_public/human/000703/WC500021146.pdf> | 14 July 2017 | Unknown* |
| Gardasil 9 | <http://www.ema.europa.eu/docs/en_GB/document_library/EPAR_-_Summary_for_the_public/human/003852/WC500189114.pdf> | 14 July 2017 | Unknown* |
| **Total studies** |  |  | **34** |

*On EMA’s website (<http://www.ema.europa.eu/ema/>) there was only access to “public” European Public Assessment Report summaries, although EMA states that, "The full EPAR [European Public Assessment Report] and risk management plan summary...can be found on the agency’s [EMA’s] website." ([http://www.ema.europa.eu/docs/en_GB/document_library/EPAR_Summary_for_the_public/human/003852/WC500189114.pdf](http://www.ema.europa.eu/docs/en_GB/document_library/EPAR_-_Summary_for_the_public/human/003852/WC500189114.pdf)). We have requested the “full” EPARs from EMA (reference ASK-32128), but we have not received any “full” EPARs yet.

Step 4: Searches on the Central Register of Controlled Trials, Google Scholar and PubMed and WikiLeaks

We searched the following databases to identify HPV vaccine studies not listed on industry, public and regulatory trial registers:

| **Database searched** | **Search phrase or term used** | **Date of search** | **Number of hits** |
| --- | --- | --- | --- |
| The Central Register of Controlled Trials: <http://onlinelibrary.wiley.com/cochranelibrary/> | "Papillomavirus Vaccines"[Mesh] AND “Trial”[Mesh] | 15 July 2017 | 241 |
| Google Scholar: [https://scholar.google.com](https://scholar.google.com/) | “Cervarix” OR “Gardasil” OR “HPV vaccine” AND “Clinical Trial” | 15 July 2017 | 1,000* [7,310] |
| PubMed: <https://www.ncbi.nlm.nih.gov/pubmed> | "Papillomavirus Vaccines"[Mesh] AND "Clinical Trial" [Publication Type] | 15 July 2017 | 302 |
| WikiLeaks: <https://wikileaks.org/> | "Human papillomavirus vaccine OR HPV vaccine OR Cervarix OR Gardasil” [We accepted: "Include external sources: Associated Twitter accounts, Snowden + Hammond Documents, Cryptome Documents, ICWatch, This Day in WikiLeaks Blog and WikiLeaks Press, WL Central"] | 15 July 2017 | 103 |
| **Total hits** |  |  | **1,646** |

*Google Scholar only shows the first 1,000 hits of any search. Therefore, we used the “sorted by relevance” function in Google Scholar and assessed the first 1,000 of 7,310 hits.

Step 5: Inclusion of studies listed on recent regulatory reviews and crosschecking of study IDs

We included HPV vaccine studies from two recent regulatory reviews and summarised steps 1 to 5 by crosschecking the identified study programme IDs.

*- Summary of identified studies/hits in step 5:*

| **Step** | **Source** | **Number of studies** |
| --- | --- | --- |
| 5a | EMAs review* of postural orthostatic tachycardia syndrome (POTS) and complex regional pain syndrome (CRPS) | 39 |
| 5b | Independent review** with studies that the EMA used in their review of postural orthostatic tachycardia syndrome (POTS) and complex regional pain syndrome (CRPS) | 41 |
| 5c | Crosschecking of study programme IDs | Not applicable |
| **Total studies** |  | **80** |

*“Review under Article 20 of Regulation (EC) No 726/2004 Human papillomavirus (HPV) vaccines.” Available from: <http://www.ema.europa.eu/docs/en_GB/document_library/Referrals_document/HPV_vaccines_20/Opinion_provided_by_Committee_for_Medicinal_Products_for_Human_Use/WC500197129.pdf>

**Jefferson T, Jørgensen L. Human papillomavirus vaccines, complex regional pain syndrome, postural orthostatic tachycardia syndrome, and autonomic dysfunction - a review of the regulatory evidence from the European Medicines Agency. Indian J Med Ethics Publ Online Oct 17 2016. Available from: <http://ijme.in/articles/human-papillomavirus-vaccines-complex-regional-pain-syndrome-postural-orthostatic-tachycardia-syndrome-and-autonomic-dysfunction-a-review-of-the-regulatory-evidence-from-the-european-medi/?galley=html>

*Step 5a:*

In 2015, GlaxoSmithKline provided EMA with a study list of Cervarix studies to be included in the EMA review of postural orthostatic tachycardia syndrome (POTS) and complex regional pain syndrome (CRPS):


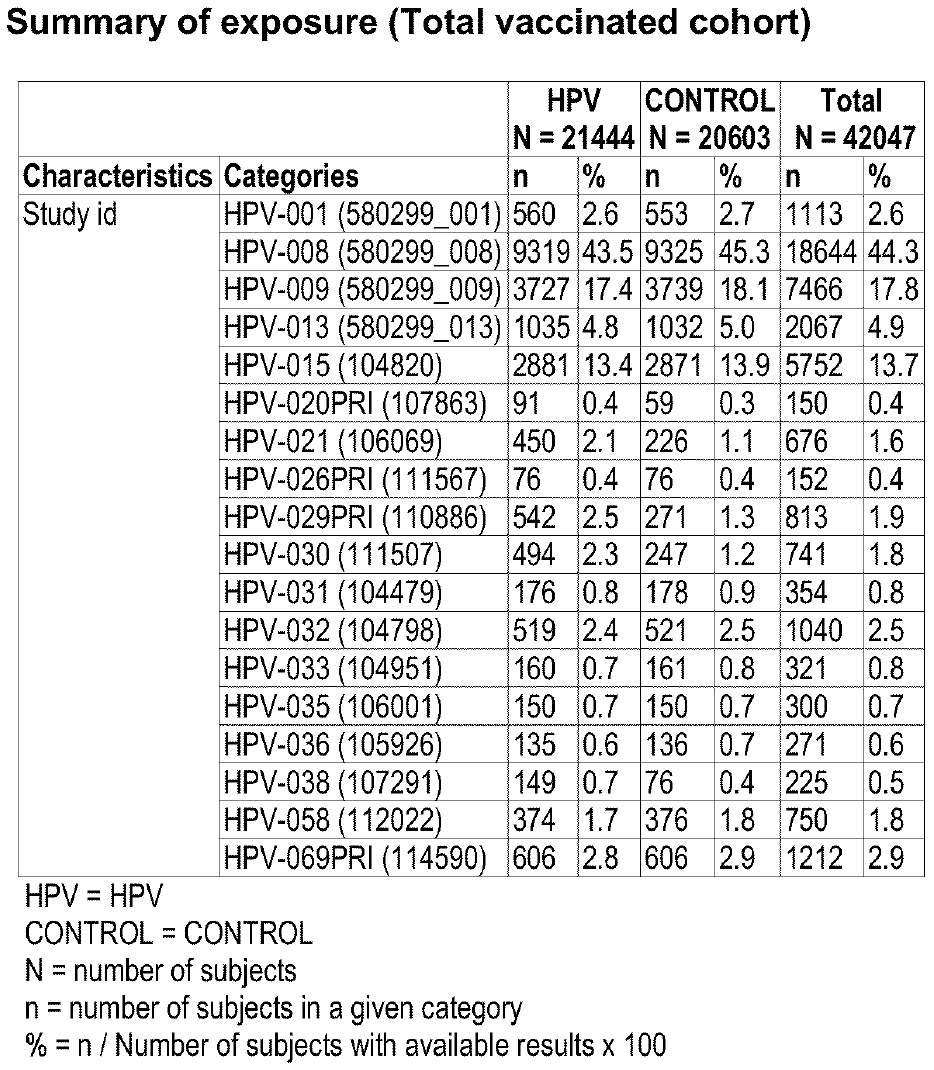


In 2015, Merck Sharp & Dohme provided the EMA with a study list of Gardasil studies to be included in the EMA review of postural orthostatic tachycardia syndrome (POTS) and complex regional pain syndrome (CRPS):


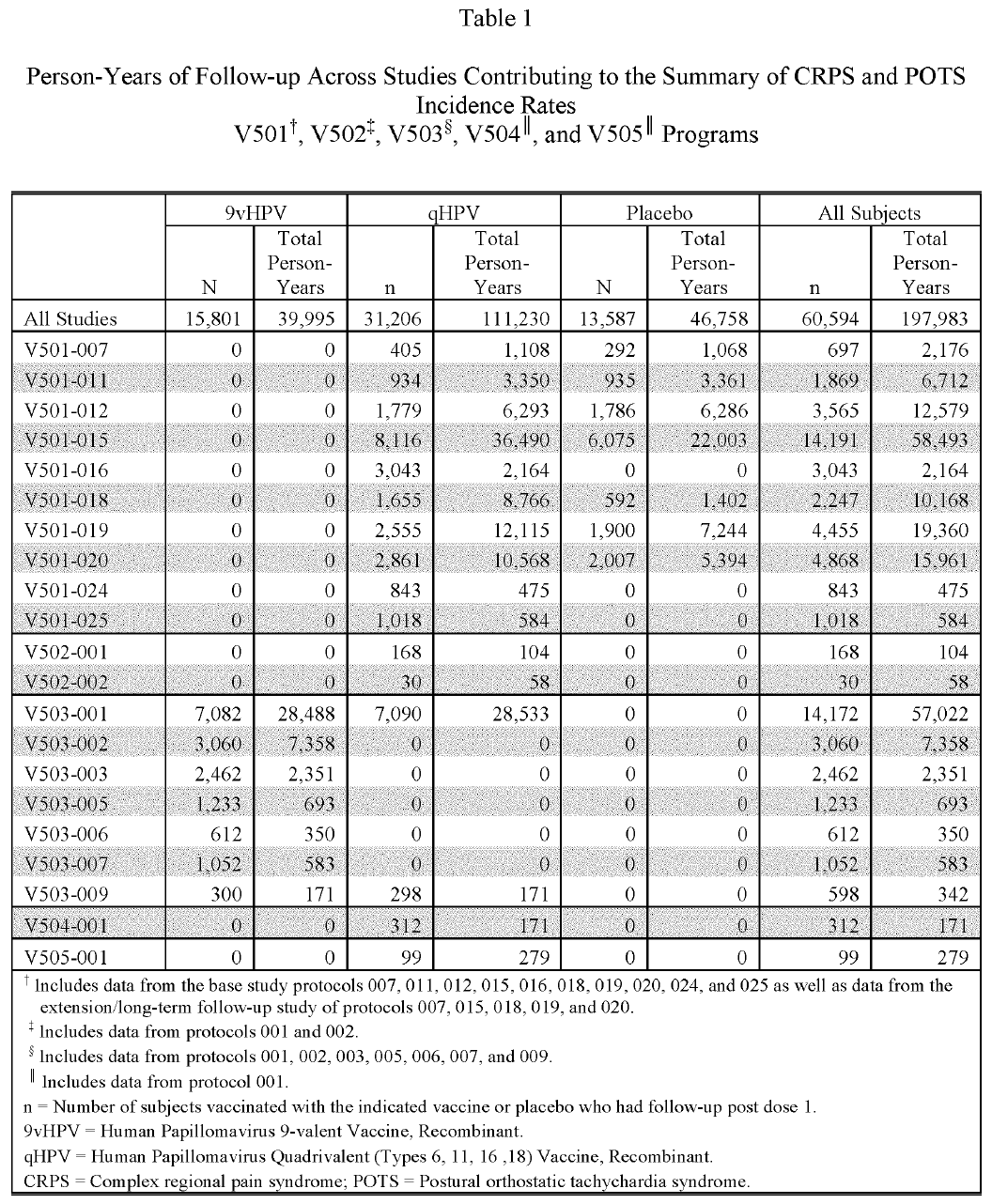


*Step 5b: Recent reviews on The HPV vaccines*

We identified studies listed in recent reviews of the HPV vaccines. In 2016, an independent review included a list of the Cervarix studies that EMA and GlaxoSmithKline used in their review of postural orthostatic tachycardia syndrome (POTS) and complex regional pain syndrome (CRPS):


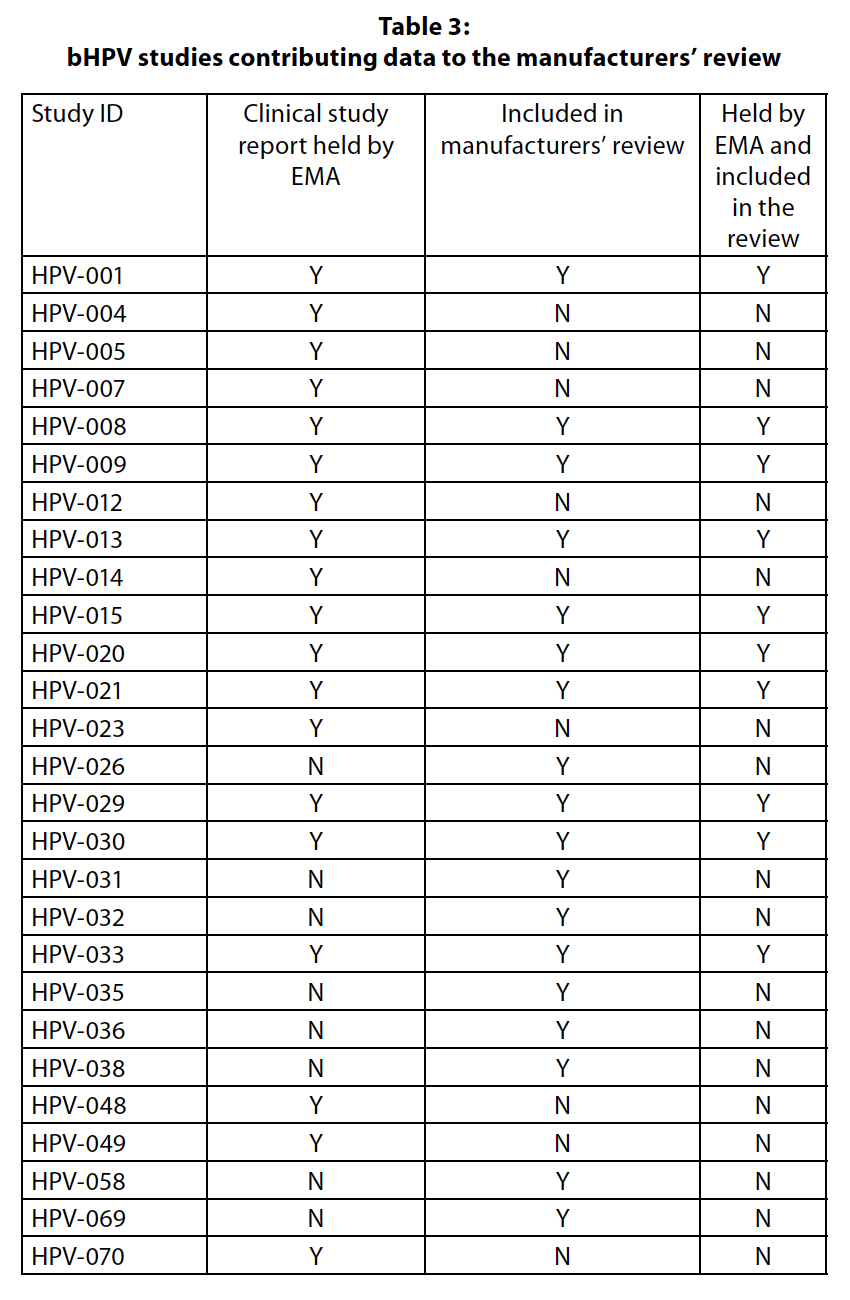


In 2016, an independent review included a list of the Gardasil studies that EMA and Merck Sharp & Dohme used in their review of postural orthostatic tachycardia syndrome (POTS) and complex regional pain syndrome (CRPS):


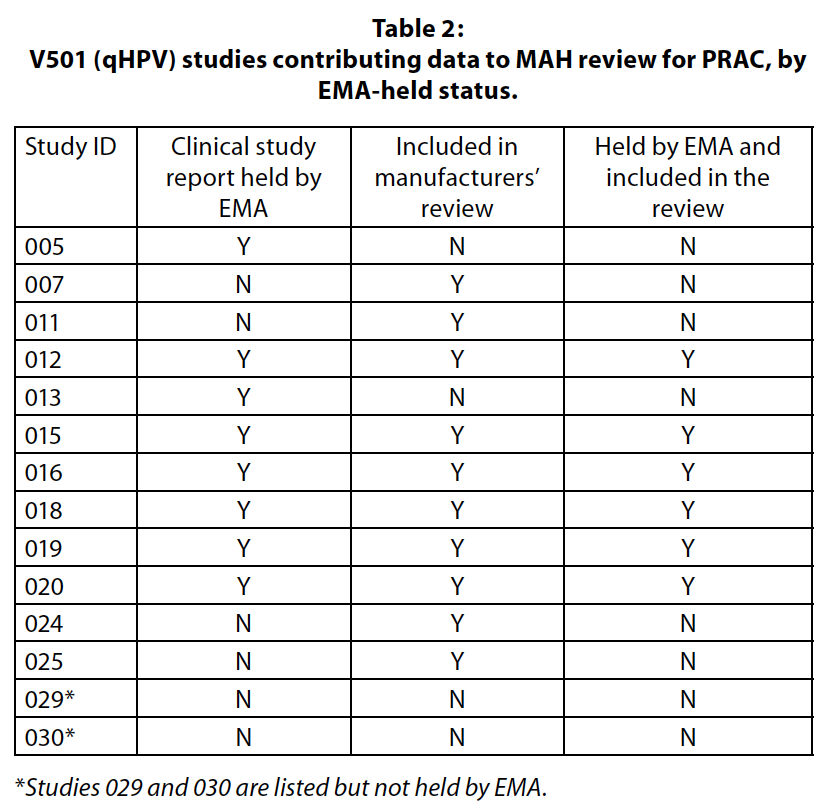


*Step 5c: Crosschecking of study identifiers*

To summarize steps 1 to 5 we crosschecked the identified industry study programme IDs. We reasoned that these IDs would be allocated in a progressive chronological way, since the main industry HPV vaccine studies are indexed as: “311-HPV-xxxx,” “HPV-xxx,” “HPV-PRO-xxx” and “V50x-xxx.” Thus, we searched [https://www.google.com](https://www.google.com/) for the following IDs:

1. GlaxoSmithKline: “HPV-001,” “HPV-002,” […], and “HPV-200.”
2. Merck Sharp & Dohme: “V501-001,” “V501-002,” […], and “V501-200.”
3. Merck Sharp & Dohme: “V502-001,” “V502-002,” […], and “V502-050.”
4. Merck Sharp & Dohme: “V503-001,” “V503-002,” […], and “V503-050.”
5. Merck Sharp & Dohme: “V504-001,” “V504-002,” […], and “V504-050.”
6. Merck Sharp & Dohme: “V505-001,” “V505-002,” […], and “V505-050.”
7. Shanghai Zerun Biotechnology Co., Ltd.: “311-HPV-1001,” “311-HPV-1002,” […] “311-HPV-1050.”
8. Xiamen Innovax Biotech Co., Ltd.: “HPV-PRO-001,” “HPV-PRO-002,” […] “HPV-PRO-050.”

We did not note the number of hits in this step, since this was merely a checking of the studies identified and the studies that could not be identified in a chronologic order, for example, we identified study “HPV-005” and study “HPV-007,” but we did not identify study “HPV-006.”

Step 6: The HPV vaccine manufacturers assessment of the study indexes accuracy

We sent the indexes of the HPV vaccines studies (gathered in the search steps 1 to 5) to their respective manufacturers for their assessment of the indexes accuracy and requested the manufacturers to confirm studies that we indexed and add any missing studies (see Appendix 2):

| **Manufacturer** | **HPV vaccine** | **Number of studies/hits**  **sent to us by the**  **manufacturers** | **Number of indexed studies confirmed by the manufacturers** | **Number of studies added to the index by the manufacturers** |
| --- | --- | --- | --- | --- |
| GlaxoSmithKline | Cervarix (bivalent) | 0 | 66 of 69 studies | 0 |
| Merck Sharp & Dohme | Gardasil (mono-, quadri-, octa- and ninevalent) | 0 | 0 of 66 studies | 0 |
| Shanghai Zerun Biotechnology | HPV vaccine (bi- and quadrivalent) | 0 | 0 of 4 studies | 0 |
| Xiamen Innovax Biotech | HPV vaccine (bivalent) | 0 | 0 of 6 studies | 0 |
| **Total** |  | **0** | **66 of 145 studies** | **0** |
