# Supplementary material for: Index of the human papillomavirus (HPV) vaccine industry clinical study programmes and non-industry funded studies: a necessary basis to address reporting bias in a systematic review
Source: Syst Rev. 2018 Jan 18;7:8. doi: 10.1186/s13643-018-0675-z (PMC5774129; doi:10.1186/s13643-018-0675-z)
Supplement: Supplementary file 2 — Index of the HPV vaccines clinical studies: Correspondence with the HPV vaccine manufacturers for the assessment of the accuracy of our indexed industry study programmes. (DOC 151 kb) [file 13643_2018_675_MOESM2_ESM.doc]

**Additional file 2: Index of the HPV vaccines clinical studies: Correspondence with the HPV vaccine manufacturers for the assessment of the accuracy of our indexed industry study programmes**

The GlaxoSmithKline* Cervarix study programme

*All GlaxoSmithKline employees’ names have been changed to ‘GSK’ and all employee email addresses have been deleted or changed to [GSK@gsk.com](mailto:GSK@gsk.com) from the correspondence.

| **GSK**<GSK@gsk.com> | 9 November 2016 at 13:44 |
| --- | --- |
| To: "lj@cochrane.dk" <lj@cochrane.dk> | |
| | Dear Dr. Jørgensen,    I have received your request below and thank you for your interest in our studies. However, in order to best address your question could you please provide some more clarity what you expect from us, especially for which parts in the Excel sheet that you shared you require our input?    Information about the GSK trials is published on [https://www.gsk-clinicalstudyregister.com](https://www.gsk-clinicalstudyregister.com/) and <https://clinicaltrials.gov/>. The information on these websites is updated regularly and should be the main source for your information and quality checks.    Would you be able to provide some more detail about the scope of the exploratory review of regulatory documents that you mention in your mail?  Thanks in advance.  Best regards,  GSK    **GSK**  **Global Medical Affairs HPV**  Vaccine Value & Health Science  GSK Vaccines    **Expéditeur:** <[lj@cochrane.dk](mailto:lj@cochrane.dk)> **Date:** 8 novembre 2016 à 11:51:55 UTC+1 **Destinataire:** <[GSK@gsk.com](mailto:soizic.m.courcier@gsk.com)> **Cc:** <[jefferson.tom@gmail.com](mailto:jefferson.tom@gmail.com)> **Objet:** **Cervarix trial programme**  Dear GSK,  We are writing to you to ask for your help in creating an accurate up-to- date list of the clinical trials (and their follow-ups) for GlaxoSmithKlines' human papillomavirus (HPV) vaccine: Cervarix™.  We are at present working on a protocol for an exploratory review of regulatory documents (mainly clinical study reports) of HPV vaccines. As a preliminary to the review and to size the task, we are making a list of all known HPV vaccine trials.  We kindly ask you to assess the attached list of currently identified Cervarix™ trials performed by GlaxoSmithKline and suggest any corrections and/or edits. (The trials marked in yellow are conjectural, for which we could find little information.)  Your help will be highly appreciated and acknowledged in any publication.  We would be grateful to receive our assessments before 9 December 2016.  Thank you for your time, We look forward to your answer.  Best wishes,  PhD student Lars Jørgensen, MD  Honorary research fellow Tom Jefferson, MD  Centre director Peter C. Gøtzsche, Professor  PS: If you are not the right person to deal with this request we would appreciate if you would pass the request on to a colleague appropriate for the task.   | | [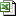](https://mail.google.com/mail/u/0/?ui=2&ik=9726daa82f&view=att&th=158491eba3b2ad65&attid=0.1&disp=attd&safe=1&zw) |  | **Cervarix trial programme.xlsx** 103K | | --- | --- | --- | | | --- | --- | --- | --- | | | --- | --- | --- | --- | --- | | |

| **Lars Jørgensen**<larsjorgensens@gmail.com> | 9 November 2016 at 17:58 |
| --- | --- |
| To: GSK <GSK@gsk.com>  Cc: lj@cochrane.dk | |
| | Dear GSK,  Thank you for your quick response and enquiry.  We have searched both [https://www.gsk-clinicalstudyregister.com](https://www.gsk-clinicalstudyregister.com/) and <https://clinicaltrials.gov/> for available Cervarix trials and follow up studies.  To clarify, we hope that you-:  1) can confirm that all the trials (and follow up studies) in our list exist.  2) can add any trials to the list that we have missed in our inclusion.  3) can add further details to listed trials (e.g. if stated NA=not available/applicable in the list) and to any missing trials.  The scope of the exploratory review of regulatory documents is to outline the resources required to carry out a systematic review of comparative evidence of the effects of HPV vaccines.  We look forward to hear from you again.  Best wishes,  Lars Jørgensen  Tom Jefferson  Peter C. Gøtzsche | | --- | | |

| **GSK** <GSK@gsk.com> | 10 November 2016 at 15:54 |
| --- | --- |
| To: Lars Jørgensen <larsjorgensens@gmail.com>  Cc: "lj@cochrane.dk" <lj@cochrane.dk> | |
| | Dear Dr. Jørgensen,    Thanks for the clarification.  As your questions concerns information that is not in the public domain, you may need to submit an official clinical study data request. However, I need to check with the responsible person how to qualify your request and I will get back to you early next week.    Best regards,    GSK    **From:** Lars Jørgensen [mailto:[larsjorgensens@gmail.com](mailto:larsjorgensens@gmail.com)]  **Sent:** mercredi 9 novembre 2016 17:58 **To:** GSK **Cc:** [lj@cochrane.dk](mailto:lj@cochrane.dk) **Subject:** Re: Your request to GSK | | --- | | |

| **Lars Jørgensen**<larsjorgensens@gmail.com> | 10 November 2016 at 17:36 |
| --- | --- |
| To: GSK <GSK@gsk.com> | |
| | Dear GSK,  Much obliged. Looking forward to hear from you.  Best wishes,  Lars | | --- | | |

| **Lars Jørgensen**<larsjorgensens@gmail.com> | 18 November 2016 at 09:15 |
| --- | --- |
| To: GSK <GSK@gsk.com> | |
| | Dear GSK,  We wonder whether you have had time to consider our request for fact checking on the list you received the 9th of November.  With best wishes,  Tom and Lars | | --- | | |

| **GSK**<GSK@gsk.com> | 18 November 2016 at 14:15 |
| --- | --- |
| To: "lj@cochrane.dk" <lj@cochrane.dk> | |
| | Dear Dr.  Jørgensen,    Thank you again for your interest in our data.    GSK is committed to sharing research data with independent researchers and organisations through our SHARE initiative. As part of this initiative, we require that all requests from independent researchers for information on GSK trials be submitted through the SHARE website (<https://clinicalstudydatarequest.com/>).    Your request will be reviewed by an Independent review panel. The Independent Review Panel comprises external experts. It accepts or rejects proposals based on the scientific rationale and relevance to medical science or patient care. Following approval of your proposal and subsequent signing of a Data sharing agreement, we can share information on our research with you including the availability of data from studies that are not listed on <https://clinicaltrials.gov/> or [https://www.gsk-clinicalstudyregister.com](https://www.gsk-clinicalstudyregister.com/). Furthermore, you can have access to patient level data as well, if needed in the course of the research project.    Additional details on the process are available on <https://clinicalstudydatarequest.com/Step-By-Step.aspx> and on <https://clinicalstudydatarequest.com/Study-Sponsors-GSK-Details.aspx>.    May I propose that you submit your request through the above process? Please, do not hesitate to come back to us with any questions concerning the submission. | | --- | | |

| **Lars Jørgensen**<larsjorgensens@gmail.com> | 22 November 2016 at 10:31 |
| --- | --- |
| To: GSK <GSK@gsk.com> | |
| | Dear GSK.  Thank you for your response.  Your help is highly appreciated.  Our primary request is for you to simply confirm that all GSKs Cervarix studies are represented by a row in our spreadsheet (the list is mainly constructed via [clinicaltrials.gov](http://clinicaltrials.gov/) and [gsk.com](http://gsk.com/)).  We do not request any publications or data. But we would like to know if the SHARE agreement includes restrictions on publications.  Best wishes,  Lars | | --- | | |

| **GSK** <GSK@gsk.com> | 23 November 2016 at 09:34 |
| --- | --- |
| To: "lj@cochrane.dk" <lj@cochrane.dk> | |
| | Dear Dr.  Jørgensen,    As I mentioned in my mail there are studies that are not listed on the websites. Information about these studies (including study design elements, sample size etc….) would be considered information that needs to be requested via SHARE.    You can just submit your enquiry according to the process below. This ensures that your request is correctly channelled.    The SHARE agreement does not include any restriction on publications. On the contrary, upon completion researchers are expected to publish their research in a peer-reviewed journal. GSK requests a copy of the publication after it has been submitted to a scientific congress or journal to assess whether there are any patent implications.  Let me know if you have further questions.    Best regards,    GSK  **From:** Lars Jørgensen [mailto:[larsjorgensens@gmail.com](mailto:larsjorgensens@gmail.com)]  **Sent:** mardi 22 novembre 2016 10:31 **To:** GSK | | --- | | |

| **Lars Jørgensen**<larsjorgensens@gmail.com> | 28 November 2016 at 14:05 |
| --- | --- |
| To: GSK <GSK@gsk.com> | |
| | Dear GSK,  We have read the agreement (attached) and are not 100% comfortable with it.  We understand that the agreement requirement is due to point 2) of our previous request:  1) can confirm that all the trials (and follow up studies) in our list exist.  2) can add any trials to the list that we have missed in our inclusion.  3) can add further details to listed trials (e.g. if stated NA=not available/applicable in the list) and to any missing trials.  We therefore kindly ask you only to assess point 1) and 3) for the attached list of currently identified Cervarix™ trials and suggest any corrections and/or edits:  1) can confirm that all the trials (and follow up studies) in our list exist.  3) can add further details to listed trials (e.g. if stated NA=not available/applicable in the list) and to any missing trials.  We hope this is acceptable and look forward to your response.  Best wishes,  Lars   | | [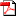](https://mail.google.com/mail/u/0/?ui=2&ik=9726daa82f&view=att&th=158ab0a42551228b&attid=0.1&disp=attd&realattid=f_iw22p91i0&safe=1&zw) |  | **DATA-SHARING-AGREEMENT.pdf** 302K | | --- | --- | --- | | | --- | --- | --- | --- | | | --- | --- | --- | --- | --- | | |

| **GSK**<GSK@gsk.com> | 29 November 2016 at 18:17 |
| --- | --- |
| To: "lj@cochrane.dk" <lj@cochrane.dk> | |
| | Dear Dr.  Jørgensen,    The SHARE platform is the central entry point for requests concerning the clinical trials. As I mentioned you can submit any request according to the process that I have laid out and it does not matter if it is your question 1, 2 or 3.  This will guarantee a correct review by our experts and the necessity to set up an agreement will be determined based on the review.    If you have any further questions, we can also offer to set up a teleconference to discuss any concerns related to the process or agreement.    Best regards,    GSK    **Global Medical Affairs HPV**  Vaccine Value & Health Science  GSK Vaccines    **From:** Lars Jørgensen [mailto:[larsjorgensens@gmail.com](mailto:larsjorgensens@gmail.com)]  **Sent:** lundi 28 novembre 2016 14:05 | | --- | | |

| **Lars Jørgensen**<larsjorgensens@gmail.com> | 17 July 2017 at 16:52 |
| --- | --- |
| To: GSK@gsk.com, GSK <GSK@gsk.com>  Cc: Tom Jefferson <jefferson.tom@gmail.com> | |
| | Dear GSK,  We are writing to you to ask for your help in creating an accurate up-to-date list of the clinical trials (and their follow-ups) for GlaxoSmithKline's human papillomavirus (HPV) vaccine: Cervarix.  We are at present working on a protocol for an exploratory review of regulatory documents (mainly clinical study reports) of HPV vaccines.  As a preliminary to the review and to size the task, we are making a list of all known interventional HPV vaccine trials that are prospective, preventive and comparative.  We wrote to GlaxoSmithKline in November 2016 with a similar request, but we did not receive any conclusive or explicit feedback on our index of Cervarix studies.  We kindly ask you to reconsider to assess the attached list of currently identified Cervarix trials and suggest any corrections and/or edits.  Your help will be highly appreciated and acknowledged in any publication.  Thank you for your time,  We look forward to your answer.  Best wishes,  PhD student Lars Jørgensen, MD  Honorary research fellow Tom Jefferson, MD  Centre director Peter C. Gøtzsche, Professor   | | [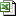](https://mail.google.com/mail/u/0/?ui=2&ik=9726daa82f&view=att&th=15d510957a9f7ff3&attid=0.1&disp=attd&realattid=f_j589prlo0&safe=1&zw) |  | **HPV vaccine trials - GlaxoSmithKline - 17 July.xlsx** 88K | | --- | --- | --- | | | --- | --- | --- | --- | | | --- | --- | --- | --- | --- | | |

| **GSK**<GSK@gsk.com> | 20 July 2017 at 10:51 |
| --- | --- |
| To: Lars Jørgensen <larsjorgensens@gmail.com>  Cc: Tom Jefferson <jefferson.tom@gmail.com> | |
| | Dear Dr. Jørgensen,    Thank you very much for your interest in our HPV vaccine clinical trial program.    We are now reviewing your new request in the context of the responses that we are preparing to the previous set of questions that have been sent by the Nordic Cochrane Center to us in June.    We will come back to you shortly on both requests.    Best regards,    GSK | | --- | | |

| **GSK**<GSK@gsk.com> | 28 July 2017 at 16:51 |
| --- | --- |
| To: Lars Jørgensen <larsjorgensens@gmail.com>  Cc: Tom Jefferson <jefferson.tom@gmail.com> | |
| | Dear Dr. Jørgensen,    I refer to your request from July 17 to review a list of clinical trials that evaluated Cervarix.    You have just been copied in our communication to Dr. Jefferson to provide responses to the six questions that were sent by Dr. Jefferson in June. We have included an overview of all GSK sponsored and Medimmune initiated clinical trials that have ever evaluated Cervarix (see again attached). This list provides you with a tool to validate the completeness of the clinical trial list that you have sent to us on July 17. Information for all trials is available on our clinical trial register website (see also our answer to question 2 in our response to Dr. Jefferson for more details). We recommend to use the GSK ID provided in the first column to search for individual trials and to navigate on our website.    In case you cannot retrieve required information for specific trials from our website, we invite you to send us a list of the trials concerned and to indicate for each trial what type of information is missing. It may require a request via the <https://www.clinicalstudydatarequest.com/> website to obtain information that is not in the public domain (eg. patient level data).    Best regards,    **GSK**  **Global Medical Affairs HPV**  Vaccine Value & Health Science     | | [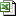](https://mail.google.com/mail/u/0/?ui=2&ik=9726daa82f&view=att&th=15d89ae88163ffc1&attid=0.1&disp=attd&safe=1&zw) |  | **Cervarix sponsored studies - Disclosure Status  July 2017.xlsx** 1502K | | --- | --- | --- | | | --- | --- | --- | --- | | | --- | --- | --- | --- | --- | | |

The Merck Sharp & Dohme Gardasil and Gardasil 9 study programme

*All Merck Sharp & Dohme employees’ names have been changed to ‘Merck’ and all employee email addresses have been changed to [Merck@merck.com](mailto:Merck@merck.com) or deleted from the correspondence.

| **lj@cochrane.dk**<lj@cochrane.dk> | 8 November 2016 at 11:06 |
| --- | --- |
| To: Merck@merck.com  Cc: larsjorgensens@gmail.com | |
| | Dear Merck,  We are writing to you to ask for your help in creating an accurate up-to-date list of the clinical trials (and their follow-ups) for Mercks' human papillomavirus (HPV) vaccine: Gardasil™.  We are at present working on a protocol for an exploratory review of regulatory documents (mainly clinical study reports) of HPV vaccines. As a preliminary to the review and to size the task, we are making a list of all known HPV vaccine trials.  We kindly ask you to assess the attached list of currently identified Gardasil™ trials performed by or in collaboration with Merck and suggest any corrections and/or edits. (The trials marked in blue are performed in collaboration with Merck, and trials marked in yellow are conjectural, for which we could find little information.)  Your help will be highly appreciated and acknowledged in any publication.  We would be grateful to receive our assessments before 8 December 2016.  Thank you for your time, We look forward to your answer.  Best wishes,  PhD student Lars Jørgensen, MD  Honorary research fellow Tom Jefferson, MD  Centre director Peter C. Gøtzsche, Professor  PS: If you are not the right person to deal with this request we would appreciate if you would pass the request on to a colleague appropriate for the task.     | | [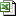](https://mail.google.com/mail/u/0/?ui=2&ik=9726daa82f&view=att&th=1584367b4ca710b1&attid=0.1&disp=attd&safe=1&zw) |  | **Gardasil trial programme.xlsx** 44K | | --- | --- | --- | | | --- | --- | --- | --- | | | --- | --- | --- | --- | --- | | |

| **Merck**<Merck@merck.com> | 8 November 2016 at 12:59 |
| --- | --- |
| Cc: "lj@cochrane.dk" <lj@cochrane.dk>, "larsjorgensens@gmail.com" <larsjorgensens@gmail.com> | |
| | Dear Merck,  I received the following query for MRL from colleagues at the Nordic Cochrane Center.  I have attached the referenced document for your review.  Best regards,  Merck     | **2 attachments** | | | --- | --- | | | [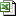](https://mail.google.com/mail/u/0/?ui=2&ik=9726daa82f&view=att&th=15843ceef5811a10&attid=0.1&disp=attd&safe=1&zw) |  | **Gardasil trial programme.xlsx** 44K | | --- | --- | --- | |  | | | [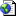](https://mail.google.com/mail/u/0/?ui=2&ik=9726daa82f&view=att&th=15843ceef5811a10&attid=0.2&disp=inline&safe=1&zw) |  | **ATT00001.htm** 1K | | --- | --- | --- | |  | | | --- | --- | --- | --- | --- | --- | --- | --- | --- | --- | --- | --- | --- | | |

| **Merck** <Merck@merck.com> | 8 November 2016 at 21:08 |
| --- | --- |
| To: "lj@cochrane.dk" <lj@cochrane.dk>  Cc: "jefferson.tom@gmail.com" <jefferson.tom@gmail.com> | |
| | Hello, Dr. Jørgensen.  You've reached the right place.  We will be happy to support. We are scoping out how much work is involved and will come back to you shortly of an estimate of when the requested information may be available.  Cheers, Merck  Merck  Global Health & Medical Affairs Merck Vaccines | | --- | | |

| **Tom Jefferson**<jefferson.tom@gmail.com> | 9 November 2016 at 08:14 |
| --- | --- |
| To: Merck <Merck@merck.com>  Cc: "lj@cochrane.dk" <lj@cochrane.dk> | |
| | Dear Merck, thank you so much for your quick and helpful answer.  We are grateful for GSK’s [Merck’s] support in our quest for factual accuracy of our work. It will be acknowledged.  Best wishes,  Tom.  Dr Tom Jefferson  Honorary Research Fellow  Centre for Evidence Based Medicine  Oxford OX2 6GG | | --- | | |

| **Lars Jørgensen**<larsjorgensens@gmail.com> | 18 November 2016 at 09:32 |
| --- | --- |
| To: Merck <Merck@merck.com> | |
| | Dear Merck,  Have you had the time to consider our request for fact checking on the list we sent you on the 8th of November?  With best wishes,  Lars Jørgensen  On 8 November 2016 at 21:08, Merck <[Merck@merck.com](mailto:john_grabenstein@merck.com)> wrote: | | --- | | |

| **Merck**<Merck@merck.com> | 21 November 2016 at 22:59 |
| --- | --- |
| To: Lars Jørgensen <larsjorgensens@gmail.com> | |
| | Lars, can you help us understand better what your request encompasses?  That will help us scope out how much work in involved.    Regarding timing, our staff is dispersing as people go out on our US harvest festival, Thanksgiving – not as large as the Chinese New Year traveling in China, nor as long perhaps as August in France, but distracting nonetheless.    Are you asking us simply to confirm that all our studies are represented by a row in your spreadsheet?  [presumably you have already consulted ClinicalTrials.gov, which we have methodically populated]    If you are asking us to fact-check each cell, this would add to the time required.    I am copying my colleague Merck, who leads our Medical Affairs efforts globally for Gardasil and Gardasil 9.    Merck | | --- | | |

| **Lars Jørgensen**<larsjorgensens@gmail.com> | 22 November 2016 at 10:09 |
| --- | --- |
| To: Merck <Merck@merck.com> | |
| | Dear Merck,  Thank you for your response and inviting Merck to the conversation.  Your help is highly appreciated.  *"Are you asking us simply to confirm that all our studies are represented by a row in your spread-sheet?"*  Yes, that is our primary request. We would appreciate if you would prioritise this. The list is mainly constructed via [clinicaltrials.gov](http://clinicaltrials.gov/) and [merck.com](http://merck.com/). (If you find the time later on to confirm/assess other aspects of the spread-sheet, we would appreciate this as well.)  Hope to hear from you soon.  Best wishes,  Lars | | --- | | |

| **Lars Jørgensen**<larsjorgensens@gmail.com> | 28 November 2016 at 16:38 |
| --- | --- |
| To: Merck@merck.com | |
| | Dear Merck,  Unfortunately our group soon has to move on in the process we are in.  We therefore hope that you in the near future will have the time to confirm  that all Merck's Gardasil studies are represented by a row in the spread-sheet  we sent on the 8th of November, 2016.  We hope you can assess this by the 8th of December 2016.  We wish to reiterate that your help will be highly appreciated and acknowledged in any publication.  Thank you for your time,  Best wishes,  Lars Jørgensen | | --- | | |

| **Merck**<Merck@merck.com> | 29 November 2016 at 23:08 |
| --- | --- |
| To: Lars Jørgensen <larsjorgensens@gmail.com> | |
| | Hello Lars,  Thanks for your note.  As Merck mentioned, some, but not all of us, have just returned from the Thanksgiving holiday.  We will do our best to get this information to you by Dec 8, but need to have the input of some members of our team who are still out on vacation this week.    Can you please tell me how these data will be used?  I am not sure I understand what is meant by a “protocol for an exploratory review of regulatory documents” mentioned in your earlier note.  Thanks for your patience,  Merck | | --- | | |

| **Merck** < Merck@merck.com> | 1 December 2016 at 18:16 |
| --- | --- |
| To: Lars Jørgensen <larsjorgensens@gmail.com> | |
| | Lars,  One more question for you, please.  Are you asking us to verify all the data in the entire document?  If so, that will take considerably more time.  Merck  **From:** Merck **Sent:** Tuesday, November 29, 2016 5:08 PM **To:** 'Lars Jørgensen' **Subject:** RE: Gardasil trial programme | | --- | | |

| **Lars Jørgensen**<larsjorgensens@gmail.com> | 1 December 2016 at 18:47 |
| --- | --- |
| To: Merck < Merck@merck.com> | |
| | Dear Merck,  Thank you for your email.  We are primarily requesting a verification of the existence of the studies that we included in the spread-sheet.  We would also like to know if we have missed any studies and what they are called (i.e. V50X-XXX).  We do not request you to verify all the data in the document, but we would appreciate a notice if you find some obvious mistakes present in the spread-sheet.  We are grateful for your help,  Best wishes,  Lars | | --- | | |

| **Merck** <Merck@merck.com> | 5 December 2016 at 06:17 |
| --- | --- |
| To: Lars Jørgensen <larsjorgensens@gmail.com> | |
| | Thanks Lars.  Can you please tell me how the data will be used (as requested in my email below of Nov 29)?  Merck | | --- | | |

| **Lars Jørgensen**<larsjorgensens@gmail.com> | 5 December 2016 at 10:26 |
| --- | --- |
| To: Merck <Merck@merck.com> | |
| | Dear Merck,  Sorry, I did not see this email: "Can you please tell me how these data will be used?  I am not sure I understand what is meant by a “protocol for an exploratory review of regulatory documents” mentioned in your earlier note."  We want to:  1) Reconstruct the complete study program of Gardasil.  2) Make a protocol for a review of the study program.  3) Analyse the data that the regulators have of the study program of Gardasil.  Best wishes,  Lars | | --- | | |

| **Lars Jørgensen**<larsjorgensens@gmail.com> | 8 December 2016 at 18:33 |
| --- | --- |
| To: Merck <Merck@merck.com> | |
| | Dear Merck,  Is there any way we may assist you in the assessment of the study list?  Sincerely,  Lars | | --- | | |

| **Lars Jørgensen**<larsjorgensens@gmail.com> | 12 December 2016 at 09:01 |
| --- | --- |
| To: Merck <Merck@merck.com> | |
| | Dear Merck,  Have you had the time to assess the study list?  Best wishes,  Lars | | --- | | |
| **Lars Jørgensen**<larsjorgensens@gmail.com> | 17 July 2017 at 16:52 |
| To: Merck@merck.com  Cc: Tom Jefferson <jefferson.tom@gmail.com> | |
| | Dear Merck,  We are writing to you to ask for your help in creating an accurate up-to-date list of the clinical trials (and their follow-ups) for Merck Sharp & Dohme's human papillomavirus (HPV) vaccines: Gardasil and Gardasil 9.  We are at present working on a protocol for an exploratory review of regulatory documents (mainly clinical study reports) of HPV vaccines.  As a preliminary to the review and to size the task, we are making a list of all known interventional HPV vaccine trials that are prospective, preventive and comparative.  We wrote to Merck Sharp & Dohme in November 2016 with a similar request, but we did not receive any conclusive or explicit feedback on our index of the Gardasil and Gardasil 9 studies.  We kindly ask you to reconsider to assess the attached list of currently identified Gardasil and Gardasil 9 trials and suggest any corrections and/or edits.  Your help will be highly appreciated and acknowledged in any publication.  Thank you for your time,  We look forward to your answer.  Best wishes,  PhD student Lars Jørgensen, MD  Honorary research fellow Tom Jefferson, MD  Centre director Peter C. Gøtzsche, Professor   | | [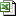](https://mail.google.com/mail/u/0/?ui=2&ik=9726daa82f&view=att&th=15d51097e6d1d1c6&attid=0.1&disp=attd&realattid=f_j589pyc30&safe=1&zw) |  | **HPV vaccine trials - Merck Sharp & Dohme - 17 July.xlsx** 82K | | --- | --- | --- | | | --- | --- | --- | --- | | | --- | --- | --- | --- | --- | | |

The Shanghai Zerun Biotechnology HPV vaccine study programme

| **lj@cochrane.dk**<lj@cochrane.dk> | 17 July 2017 at 10:30 |
| --- | --- |
| To: zerunbd@walvax.com  Cc: larsjorgensens@gmail.com | |
| | Dear Shanghai Zerun Biotechnology Co., Ltd.,  We are writing to you to ask for your help in creating an accurate up-to- date list of the clinical trials (and their follow-ups) for Shanghai Zerun Biotechnology Co., Ltd's human papillomavirus (HPV) vaccines.  We are at present working on a protocol for an exploratory review of regulatory documents (mainly clinical study reports) of HPV vaccines. As a preliminary to the review and to size the task, we are making a list of all known HPV vaccine trials.  We kindly ask you to assess the attached list of currently identified Shanghai Zerun Biotechnology Co., Ltd. HPV vaccine trials and suggest any corrections and/or edits.  Your help will be highly appreciated and acknowledged in any publication.  We would appreciate your response before 17 August.  Thank you for your time, We look forward to your answer.  Best wishes,  PhD student Lars Jørgensen, MD  Honorary research fellow Tom Jefferson, MD  Centre director Peter C. Gøtzsche, Professor  The Nordic Cochrane Centre Rigshospitalet dept. 7811 Blegdamsvej 9 2100 Copenhagen Denmark | | --- | | |

| **lj@cochrane.dk**<lj@cochrane.dk> | 27 July 2017 at 8:17 |
| --- | --- |
| To: zerunbd@walvax.com  Cc: larsjorgensens@gmail.com | |
| | Dear Shanghai Zerun Biotechnology Co., Ltd.,  We hope that you have received our previous email about your HPV vaccine studies.  If you have any questions, please do not hesitate to contact us.  Thank you for your time, We look forward to your answer.  Best wishes, Lars Jørgensen, MD Tom Jefferson, MD Peter C. Gøtzsche, Professor | | --- | | |

The Xiamen Innovax Biotech HPV vaccine study programme

| **lj@cochrane.dk**<lj@cochrane.dk> | 15 July 2017 at 13:09 |
| --- | --- |
| To: 400-001-8508@innovax.cn  Cc: larsjorgensens@gmail.com, jefferson.tom@gmail.com | |
| | Dear Xiamen Innovax Biotech Co., Ltd.,  We are writing to you to ask for your help in creating an accurate up-to-date list of the clinical trials (and their follow-ups) for Xiamen Innovax Biotech Co., Ltd's bivalent human papillomavirus (HPV) vaccine.  We are at present working on a protocol for an exploratory review of regulatory documents (mainly clinical study reports) of HPV vaccines. As a preliminary to the review and to size the task, we are making a list of all known HPV vaccine trials.  We kindly ask you to assess the attached list of currently identified Xiamen Innovax Biotech Co., Ltd HPV vaccine trials and suggest any corrections and/or edits.  Your help will be highly appreciated and acknowledged in any publication.  We would appreciate your response before 15 August.  Thank you for your time, We look forward to your answer.  Best wishes,  PhD student Lars Jørgensen, MD  Honorary research fellow Tom Jefferson, MD  Centre director Peter C. Gøtzsche, Professor  The Nordic Cochrane Centre Rigshospitalet dept. 7811 Blegdamsvej 9 2100 Copenhagen Denmark | | --- | | |

| **lj@cochrane.dk**<lj@cochrane.dk> | 27 July 2017 at 8:23 |
| --- | --- |
| To: 400-001-8508@innovax.cn  Cc: larsjorgensens@gmail.com, jefferson.tom@gmail.com | |
| | Dear Xiamen Innovax Biotech Co., Ltd.,  We hope that you have received our previous email about your HPV vaccine studies.  If you have any questions, please do not hesitate to contact us.  Thank you for your time, We look forward to your answer.  Best wishes, Lars Jørgensen, MD Tom Jefferson, MD Peter C. Gøtzsche, Professor | | --- | | |
