# Supplementary material for: Index of the human papillomavirus (HPV) vaccine industry clinical study programmes and non-industry funded studies: a necessary basis to address reporting bias in a systematic review
Source: Syst Rev. 2018 Jan 18;7:8. doi: 10.1186/s13643-018-0675-z (PMC5774129; doi:10.1186/s13643-018-0675-z)
Supplement: Supplementary file 3 — Index of the HPV vaccines clinical studies: Indexes of the identified industry study programmes and non-industry funded clinical studies and a list of the identified corresponding journal publications. (DOC 659 kb) [file 13643_2018_675_MOESM3_ESM.doc]

**Additional file 3: Index of the HPV vaccines clinical studies: Indexes of the identified industry study programmes and non-industry clinical studies and a list of the identified corresponding publications**

Index of identified HPV vaccine industry study programmes

The following indexes are the manufacturers study programmes listed in a chronological fashion according to their study programme IDs:

Index of the GlaxoSmithKline Cervarix study programme:

| **No.** | **Study classification** | **Study programme specific ID** | **Manufacturer specific ID** | **National Clinical Trial (NCT) ID** | **Verification from 1^st^ source** | **Verification from 2^nd^ source** |
| --- | --- | --- | --- | --- | --- | --- |
| 1 | ‘Definitely exists’ | HPV-001 | 580299/001 | NCT00689741 | <https://clinicaltrials.gov/show/NCT00689741> | <https://www.gsk-clinicalstudyregister.com/study/580299/001> |
| 2 | ‘Definitely exists’ | HPV-001-NG-PRI | 109836 | NCT00478621 | <https://clinicaltrials.gov/show/NCT00478621> | [https://www.gsk-clinicalstudyregister.com/study/109836](https://www.gsk-clinicalstudyregister.com/study/109836?search=compound&compound=human-papillomavirus-types-16-and-18-vaccine#ps) |
| 3 | ‘Definitely exists’ | HPV-002 | 580299/002 | Not identified | <https://www.gsk-clinicalstudyregister.com/study/580299/002> | GlaxoSmithKline’s study list |
| 4 | ‘Definitely exists’ | HPV-003 | 580299/003 | Not identified | <https://www.fda.gov/downloads/BiologicsBloodVaccines/Vaccines/ApprovedProducts/UCM237976.pdf> | <http://www.tandfonline.com/doi/abs/10.4161/hv.5.5.7211> |
| 5 | ‘Definitely exists’ | HPV-004 | 580299/004 | NCT00693615 | <https://clinicaltrials.gov/show/NCT00693615> | <https://www.gsk-clinicalstudyregister.com/study/580299/004> |
| 6 | ‘Definitely exists’ | HPV-005 | 580299/005 | NCT00693966 | <https://clinicaltrials.gov/show/NCT00693966> | <https://www.gsk-clinicalstudyregister.com/study/580299/005> |
| 7 | ‘Definitely exists’ | HPV-007 (HPV-001 follow-up) | 580299/007 | NCT00120848 | <https://clinicaltrials.gov/show/NCT00120848> | <https://www.gsk-clinicalstudyregister.com/study/580299/007> |
| 8 | ‘Definitely exists’ | HPV-008 | 580299/008 | NCT00122681 | <https://clinicaltrials.gov/show/NCT00122681> | <https://www.gsk-clinicalstudyregister.com/study/580299/008> |
| 9 | ‘Definitely exists’ | HPV-009 | 580299/009 | NCT00128661 | <https://clinicaltrials.gov/show/NCT00128661> | <http://www.sciencedirect.com/science/article/pii/S0002937816003094> |
| 10 | ‘Definitely exists’ | HPV-010 | 108933 | NCT00423046 | <https://clinicaltrials.gov/show/NCT00423046> | <https://www.gsk-clinicalstudyregister.com/study/108933> |
| 11 | ‘Definitely exists’ | HPV-011 | 580299/011 | NCT00309166 | <https://clinicaltrials.gov/show/NCT00309166> | <https://www.gsk-clinicalstudyregister.com/study/580299/011> |
| 12 | ‘Definitely exists’ | HPV-011-EPI  VS FR PMS | 112677 | NCT01498627 | [https://clinicaltrials.gov/show/NCT01498627](https://clinicaltrials.gov/ct2/show/NCT01498627?term=bivalent+HPV+OR+quadrivalent+HPV+OR+HPV+vaccine+OR+human+papillomavirus+vaccine+OR+Cervarix+OR+Gardasil+OR+Gardasil+4+OR+Gardasil+9&rank=141&submit_fld_opt=) | [https://www.gsk-clinicalstudyregister.com/study/112677](https://www.gsk-clinicalstudyregister.com/study/112677?search=compound&compound=human-papillomavirus-types-16-and-18-vaccine#ps) |
| 13 | ‘Definitely exists’ | HPV-012 | 580299/012 107476 107477 107479 107481 | NCT00169494 NCT00337818 | <https://clinicaltrials.gov/show/NCT00337818> | <https://www.gsk-clinicalstudyregister.com/study/580299/012> |
| 14 | ‘Definitely exists’ | HPV-013 | 580299/013 104904 | NCT00196924 | <https://clinicaltrials.gov/show/NCT00196924> | <https://www.gsk-clinicalstudyregister.com/study/580299/013> |
| 15 | ‘Definitely exists’ | HPV-013 follow-up | 104802 104904 104918 104996 | NCT00316706 | <https://clinicaltrials.gov/show/NCT00316706> | <https://www.gsk-clinicalstudyregister.com/study/104896> |
| 16 | ‘Definitely exists’ | HPV-014 | 103514 104902 104904 104918 | NCT00196937 | <https://clinicaltrials.gov/show/NCT00196937> | <https://www.gsk-clinicalstudyregister.com/study/103514> |
| 17 | ‘Definitely exists’ | HPV-015 | 104820 | NCT00294047 | <https://clinicaltrials.gov/show/NCT00294047> | <https://www.gsk-clinicalstudyregister.com/study/104820> |
| 18 | ‘Definitely exists’ | HPV-015-EPI  VS US DB | 113522 | NCT01153906 | [https://clinicaltrials.gov/show/NCT01153906](https://clinicaltrials.gov/ct2/show/NCT01153906?term=bivalent+HPV+OR+quadrivalent+HPV+OR+HPV+vaccine+OR+human+papillomavirus+vaccine+OR+Cervarix+OR+Gardasil+OR+Gardasil+4+OR+Gardasil+9&rank=168) | <https://www.gsk-clinicalstudyregister.com/study/113522> |
| 19 | ‘Definitely exists’ | HPV-016 | 104772 | NCT00250276 | <https://clinicaltrials.gov/show/NCT00250276> | <https://www.gsk-clinicalstudyregister.com/study/104772> |
| 20 | ‘Definitely exists’ | HPV-018 | 107682 | NCT00369824 | <https://clinicaltrials.gov/show/NCT00369824> | <https://www.gsk-clinicalstudyregister.com/study/107682> |
| 21 | ‘Definitely exists’ | HPV-018-EPI VS UK DB | 114101 | NCT01905462 | <https://clinicaltrials.gov/show/NCT01905462> | <https://www.gsk-clinicalstudyregister.com/study/114101> |
| 22 | ‘Definitely exists’ | HPV-019-PRI | 109823 | NCT01031069 | <https://clinicaltrials.gov/show/NCT01031069> | <https://www.gsk-clinicalstudyregister.com/study/109823> |
| 23 | ‘Definitely exists’ | HPV-020-EPI  VS US | 114176 | NCT01290393 | <https://clinicaltrials.gov/show/NCT01290393> | <http://www.gsk-clinicalstudyregister.com/files2/114176> |
| 24 | ‘Definitely exists’ | HPV-020-PRI | 107863 | NCT00586339 | <https://clinicaltrials.gov/show/NCT00586339> | <https://www.gsk-clinicalstudyregister.com/study/107863> |
| 25 | ‘Definitely exists’ | HPV-021 | 106069 | NCT00481767 | <https://clinicaltrials.gov/show/NCT00481767> | <https://www.gsk-clinicalstudyregister.com/study/106069> |
| 26 | ‘Definitely exists’ | HPV-023 (HPV-001 follow-up) | 109616 109624 109625 | NCT00518336 | <https://clinicaltrials.gov/show/NCT00518336> | <https://www.gsk-clinicalstudyregister.com/study/109624> |
| 27 | ‘Definitely exists’ | HPV-023-PRI (HPV-001 follow-up) | 109616 | NCT00518336 | <https://clinicaltrials.gov/show/NCT00518336> | <https://www.gsk-clinicalstudyregister.com/study/109616> |
| 28 | ‘Definitely exists’ | HPV-024 (HPV-001 follow-up) | 109628 | NCT00546078 | <https://clinicaltrials.gov/show/NCT00546078> | <https://www.gsk-clinicalstudyregister.com/study/109628> |
| 29 | ‘Definitely exists’ | HPV-025 (HPV-013 follow-up) | 111375 | NCT00877877 | <https://clinicaltrials.gov/show/NCT00877877> | <http://gsk-clinicalstudyregister.com/files2/baee5139-abc9-4887-be66-eb8e2ad444af> |
| 30 | ‘Definitely exists’ | HPV-026-PRI | 111567 | NCT00637195 | <https://clinicaltrials.gov/show/NCT00637195> | <https://www.ncbi.nlm.nih.gov/pmc/articles/PMC3165228/> |
| 31 | ‘Definitely exists’ | HPV-027 (HPV-008 follow-up) | 115006 | NCT01393470 | <https://clinicaltrials.gov/show/NCT01393470> | <http://www.clinicaltrialfacts.com/Uterine-Cervical-Cancer/Evaluation-of-Long-term-HPV-Vaccine-Effi-110268> |
| 32 | ‘Definitely exists’ | HPV-028 (HPV-015 follow-up) | 109801 | NCT00456807 | <https://clinicaltrials.gov/show/NCT00456807> | <https://www.gsk-clinicalstudyregister.com/study/109801> |
| 33 | ‘Definitely exists’ | HPV-029-PRI | 110886 | NCT00578227 | <https://clinicaltrials.gov/show/NCT00578227> | <https://www.gsk-clinicalstudyregister.com/study/110886> |
| 34 | ‘Definitely exists’ | HPV-030 | 111507 | NCT00652938 | <https://clinicaltrials.gov/show/NCT00652938> | <https://www.gsk-clinicalstudyregister.com/study/111507> |
| 35 | ‘Definitely exists’ | HPV-031 | 104479 | NCT00344032 | <https://clinicaltrials.gov/show/NCT00344032> | <http://www.gsk-clinicalstudyregister.com/files2/20003.pdf> |
| 36 | ‘Definitely exists’ | HPV-032 | 104798 | NCT00316693 | <https://clinicaltrials.gov/show/NCT00316693> | <https://www.gsk-clinicalstudyregister.com/study/104798> |
| 37 | ‘Definitely exists’ | HPV-033 | 104951 | NCT00290277 | <https://clinicaltrials.gov/show/NCT00290277> | <https://www.gsk-clinicalstudyregister.com/study/104951> |
| 38 | ‘Definitely exists’ | HPV-035 | 106001 | NCT00306241 | <https://clinicaltrials.gov/show/NCT00306241> | <https://www.gsk-clinicalstudyregister.com/study/106001> |
| 39 | ‘Definitely exists’ | HPV-036 | 105926 | NCT00345878 | <https://clinicaltrials.gov/show/NCT00345878> | <https://www.gsk-clinicalstudyregister.com/study/105926> |
| 40 | ‘Definitely exists’ | HPV-038 | 107291 | NCT00485732 | <https://clinicaltrials.gov/show/NCT00485732> | <https://www.gsk-clinicalstudyregister.com/study/107291> |
| 41 | ‘Definitely exists’ | HPV-039 | 107638 | NCT00779766 | <https://clinicaltrials.gov/show/NCT00779766> | <https://www.gsk-clinicalstudyregister.com/study/107638> |
| 42 | ‘Definitely exists’ | HPV-040-EPI VS UK (HPV-015 follow-up) | 116239 | NCT01953822 | <https://clinicaltrials.gov/show/NCT01953822> | <https://www.gsk-clinicalstudyregister.com/study/116239> |
| 43 | ‘Definitely exists’ | HPV-040-PRI | 106636 | NCT00534638 | <https://clinicaltrials.gov/show/NCT00534638> | <http://www.ema.europa.eu/docs/en_GB/document_library/EPAR_-_Assessment_Report_-_Variation/human/000721/WC500212077.pdf> |
| 44 | ‘Definitely exists’ | HPV-042 | 108464 | NCT00426361 | <https://clinicaltrials.gov/show/NCT00426361> | <https://www.gsk-clinicalstudyregister.com/study/108464> |
| 45 | ‘Definitely exists’ | HPV-044-PRI | 109179 | NCT00552279 | <https://clinicaltrials.gov/show/NCT00552279> | <https://www.gsk-clinicalstudyregister.com/study/109179> |
| 46 | ‘Definitely exists’ | HPV-048-PRI | 110659 | NCT00541970 | <https://clinicaltrials.gov/show/NCT00541970> | <https://www.gsk-clinicalstudyregister.com/study/110659> |
| 47 | ‘Definitely exists’ | HPV-049 | Not identified | Not identified | <http://www.ema.europa.eu/docs/en_GB/document_library/EPAR_-_Assessment_Report_-_Variation/human/000721/WC500121597.pdf> | EMA email from 18 October 2016 |
| 48 | ‘Definitely exists’ | HPV-051-TETRA | 102115 | NCT00231413 | <https://clinicaltrials.gov/show/NCT00231413> | <http://www.sciencedirect.com/science/article/pii/S0264410X14004095> |
| 49 | ‘Definitely exists’ | HPV-051 follow-up | 107918 107919 107921 108052 | NCT00359619 | <https://clinicaltrials.gov/show/NCT00359619> | <https://www.gsk-clinicalstudyregister.com/study/108052> |
| 50 | ‘Definitely exists’ | HPV-052 (HPV-008 follow-up) | 112024 | NCT00937950 | <https://clinicaltrials.gov/show/NCT00937950> | <https://www.gsk-clinicalstudyregister.com/study/112024> |
| 51 | ‘Definitely exists’ | HPV-055 (HPV-008 follow-up) | 111758 | NCT00849381 | <https://clinicaltrials.gov/show/NCT00849381> | <https://www.gsk-clinicalstudyregister.com/study/111758> |
| 52 | ‘Definitely exists’ | HPV-056 (HPV-035 follow-up) | 111712 | NCT00811798 | <https://clinicaltrials.gov/show/NCT00811798> | <https://clinicalstudydatarequest.com/Posting.aspx?PostingID=212> |
| 53 | ‘Definitely exists’ | HPV-057 (HPV-008 follow-up) | 111955 | NCT00799825 | <https://clinicaltrials.gov/show/NCT00799825> | <https://www.gsk-clinicalstudyregister.com/study/111955> |
| 54 | ‘Definitely exists’ | HPV-058 (HPV-069 co-study) | 112022 | NCT00996125 | <https://clinicaltrials.gov/show/NCT00996125> | <https://www.gsk-clinicalstudyregister.com/study/112022> |
| 55 | ‘Definitely exists’ | HPV-060 (HPV-013 follow-up) | 112772 | NCT00947115 | <https://clinicaltrials.gov/show/NCT00947115> | <https://www.gsk-clinicalstudyregister.com/study/112772> |
| 56 | ‘Definitely exists’ | HPV-062 (HPV-015 follow-up) | 113617 | NCT01190176 | <https://clinicaltrials.gov/show/NCT01190176> | <https://www.gsk-clinicalstudyregister.com/study/113617> |
| 57 | ‘Definitely exists’ | HPV-063 (HPV-032 follow-up) | 112949 | NCT00929526 | <https://clinicaltrials.gov/show/NCT00929526> | <https://www.gsk-clinicalstudyregister.com/study/112949> |
| 58 | ‘Definitely exists’ | HPV-066 (HPV-015 follow-up) | 113618 | NCT01249365 | <https://clinicaltrials.gov/show/NCT01249365> | <https://www.gsk-clinicalstudyregister.com/study/113618> |
| 59 | ‘Definitely exists’ | HPV-067 (HPV-015 follow-up) | 113621 | NCT01190189 | <https://clinicaltrials.gov/show/NCT01190189> | <https://www.gsk-clinicalstudyregister.com/study/113621> |
| 60 | ‘Definitely exists’ | HPV-068 (HPV-023 follow-up) | 114379 | NCT01418937 | <https://clinicaltrials.gov/show/NCT01418937> | <https://www.gsk-clinicalstudyregister.com/study/114379> |
| 61 | ‘Definitely exists’ | HPV-069-PRI (HPV-058 co-study) | 114590 | NCT01277042 | <https://clinicaltrials.gov/show/NCT01277042> | <https://www.gsk-clinicalstudyregister.com/study/114590> |
| 62 | ‘Definitely exists’ | HPV-070 | 114700 | NCT01381575 | <https://clinicaltrials.gov/show/NCT01381575> | <https://www.gsk-clinicalstudyregister.com/study/114700> |
| 63 | ‘Definitely exists’ | HPV-071-PRI | 115411 | NCT01462357 | <https://clinicaltrials.gov/show/NCT01462357> | <https://www.gsk-clinicalstudyregister.com/study/115411> |
| 64 | ‘Definitely exists’ | HPV-073 | 115887 | NCT01627561 | [https://clinicaltrials.gov/show/NCT01627561](https://clinicaltrials.gov/ct2/show/NCT01627561?term=bivalent+HPV+OR+quadrivalent+HPV+OR+HPV+vaccine+OR+human+papillomavirus+vaccine+OR+Cervarix+OR+Gardasil+OR+Gardasil+4+OR+Gardasil+9&rank=181&submit_fld_opt=) | [https://www.gsk-clinicalstudyregister.com/study/115887](https://www.gsk-clinicalstudyregister.com/study/115887?search=compound&compound=human-papillomavirus-types-16-and-18-vaccine#ps) |
| 65 | ‘Definitely exists’ | HPV-078 | 117099 | NCT02082639 | <https://clinicaltrials.gov/show/NCT02082639> | <https://www.gsk-clinicalstudyregister.com/study/117099> |
| 66 | ‘Definitely exists’ | HPV-081  (HPV-048 follow-up) | 200255 | Not identified | <http://apps.who.int/trialsearch/Trial2.aspx?TrialID=EUCTR2014-000060-17-DE> | <https://www.clinicaltrialsregister.eu/ctr-search/trial/2014-000060-17/DE> |
| 67 | ‘Definitely exists’ | HPV-111103-EPI | 111103 | Not identified | <http://apps.who.int/trialsearch/Trial2.aspx?TrialID=EUCTR2007-006651-39-GB> | <https://www.clinicaltrialsregister.eu/ctr-search/trial/2007-006651-39/GB> |
| 68 | ‘Definitely exists’ | MENACWY-TT-054 | 113823 | NCT01755689 | [https://clinicaltrials.gov/show/NCT01755689](https://clinicaltrials.gov/ct2/show/NCT01755689?term=bivalent+HPV+OR+quadrivalent+HPV+OR+HPV+vaccine+OR+human+papillomavirus+vaccine+OR+Cervarix+OR+Gardasil+OR+Gardasil+4+OR+Gardasil+9&rank=148&submit_fld_opt=) | <https://www.gsk-clinicalstudyregister.com/study/113823> |
| 69 | ‘Definitely exists’ | Not identified | 113763 | NCT01551537 | [https://clinicaltrials.gov/show/NCT01551537](https://clinicaltrials.gov/ct2/show/NCT01551537?term=bivalent+HPV+OR+quadrivalent+HPV+OR+HPV+vaccine+OR+human+papillomavirus+vaccine+OR+Cervarix+OR+Gardasil+OR+Gardasil+4+OR+Gardasil+9&rank=122) | [https://www.gsk-clinicalstudyregister.com/study/113763](https://www.gsk-clinicalstudyregister.com/study/113763?search=compound&compound=human-papillomavirus-types-16-and-18-vaccine#ps) |

Index of the Merck Sharp & Dohme Gardasil and Gardasil 9 study programmes:

| **No.** | **Study classification** | **Study programme specific ID** | **Manufacturer specific ID** | **National Clinical Trial (NCT) ID** | **Verification from 1^st^ source** | **Verification from 2^nd^ source** |
| --- | --- | --- | --- | --- | --- | --- |
| 1 | ‘Probably exists’ | V501-001 | Not identified | Not identified | <http://www.fda.gov/ohrms/dockets/ac/06/slides/2006-4222S-2_files/frame.htm> | Not identified |
| 2 | ‘Probably exists’ | V501-002 | Not identified | Not identified | <http://www.fda.gov/ohrms/dockets/ac/06/slides/2006-4222S-2_files/frame.htm> | Not identified |
| 3 | ‘Probably exists’ | V501-004 | Not identified | Not identified | <http://www.fda.gov/ohrms/dockets/ac/06/slides/2006-4222S-2_files/frame.htm> | Not identified |
| 4 | ‘Definitely exists’ | V501-005 | 2006_515 | NCT00365378 | [https://clinicaltrials.gov/show/NCT00365378](https://clinicaltrials.gov/ct2/show/NCT00365378?term=V501-005&rank=1) | <http://www.merck.com/clinical-trials/study.html?id=V501-005&kw=gardasil> |
| 5 | ‘Probably exists’ | V501-005 follow-up | Not identified | Not identified | [https://www.ncbi.nlm.nih.gov/pmc/articles/PMC2749988/#R10](https://www.ncbi.nlm.nih.gov/pmc/articles/PMC2749988/#R10) | Not identified |
| 6 | ‘Definitely exists’ | V501-007 | 2006_516 | NCT00365716 | [https://clinicaltrials.gov/show/NCT00365716](https://clinicaltrials.gov/ct2/show/NCT00365716?term=V501-007&rank=1) | <http://www.merck.com/clinical-trials/study.html?id=V501-007&kw=gardasil> |
| 7 | ‘Definitely exists’ | V501-011 | 2007_576 | NCT00517309 | [https://clinicaltrials.gov/show/NCT00517309](https://clinicaltrials.gov/ct2/show/NCT00517309?term=NCT00517309&rank=1) | <https://www.ncbi.nlm.nih.gov/pubmed/18164106?dopt=Abstract> |
| 8 | ‘Definitely exists’ | V501-012 | 2004_080 | NCT00092482 | [https://clinicaltrials.gov/show/NCT00092482](https://clinicaltrials.gov/ct2/show/NCT00092482?term=NCT00092482&rank=1) | <https://www.ncbi.nlm.nih.gov/pmc/articles/PMC1951095/> |
| 9 | ‘Definitely exists’ | V501-013 | 2004_081 | NCT00092521 | <https://clinicaltrials.gov/show/NCT00092521> | <http://www.merck.com/clinical-trials/study.html?id=V501-013&kw=gardasil> |
| 10 | ‘Definitely exists’ | V501-015 | 2004_082 | NCT00092534 | [https://clinicaltrials.gov/show/NCT00092534](https://clinicaltrials.gov/ct2/show/NCT00092534?term=NCT00092534&rank=1) | <http://www.merck.com/clinical-trials/study.html?id=V501-015&kw=gardasil> |
| 11 | ‘Definitely exists’ | V501-016 | 2004_083 | NCT00092495 | [https://clinicaltrials.gov/show/NCT00092495](https://clinicaltrials.gov/ct2/show/NCT00092495?term=V501-016&rank=1) | <http://www.merck.com/clinical-trials/study.html?id=V501-016&kw=gardasil> |
| 12 | ‘Definitely exists’ | V501-018 | 2004_084 | NCT00092547 | [https://clinicaltrials.gov/show/NCT00092547](https://clinicaltrials.gov/ct2/show/NCT00092547?term=V501-018&rank=1) | <http://www.merck.com/clinical-trials/study.html?id=V501-018&kw=gardasil> |
| 13 | ‘Definitely exists’ | V501-019 | 2004_013 | NCT00090220 | [https://clinicaltrials.gov/show/NCT00090220](https://clinicaltrials.gov/ct2/show/NCT00090220) | <http://www.merck.com/clinical-trials/study.html?id=V501-019&kw=gardasil> |
| 14 | ‘Definitely exists’ | V501-020 | 2004_103 | NCT00090285 | [https://clinicaltrials.gov/show/NCT00090285](https://clinicaltrials.gov/ct2/show/NCT00090285?term=V501-020&rank=1) | <http://www.merck.com/clinical-trials/study.html?id=V501-020&kw=gardasil> |
| 15 | ‘Definitely exists’ | V501-023 | 2005_066 | NCT00157950 | [https://clinicaltrials.gov/show/NCT00157950](https://clinicaltrials.gov/ct2/show/NCT00157950?term=gardasil&rank=3) | <https://www.ncbi.nlm.nih.gov/pubmed/17986242?dopt=Abstract> |
| 16 | ‘Definitely exists’ | V501-024 | 2005_093 | NCT00337428 | <https://clinicaltrials.gov/show/NCT00337428> | <http://www.clinicaltrialfacts.com/Neoplasms-Glandular-and-Epithelial/Concomitant-Use-of-Gardasil-V501-Human-29955> |
| 17 | ‘Definitely exists’ | V501-025 | 2005_092 | NCT00325130 | [https://clinicaltrials.gov/show/NCT00325130](https://clinicaltrials.gov/ct2/show/NCT00325130?term=V501-025&rank=1) | <http://www.clinicaltrialfacts.com/Neoplasms-Glandular-and-Epithelial/Concomitant-Use-of-Gardasil-V501-Huma-29044> |
| 18 | ‘Definitely exists’ | V501-027 | 2006_032 | NCT00378560 | [https://clinicaltrials.gov/show/NCT00378560](https://clinicaltrials.gov/ct2/show/NCT00378560?term=NCT00378560&rank=1) | <http://www.merck.com/clinical-trials/study.html?id=V501-027&kw=gardasil> |
| 19 | ‘Definitely exists’ | V501-028 | 2006_052 | NCT00411749 | [https://clinicaltrials.gov/show/NCT00411749](https://clinicaltrials.gov/ct2/show/NCT00411749?term=V501-028&rank=1) | <http://www.merck.com/clinical-trials/study.html?id=V501-028&kw=gardasil> |
| 20 | ‘Definitely exists’ | V501-030 | 2007_021 | NCT00496626 | [https://clinicaltrials.gov/show/NCT00496626](https://clinicaltrials.gov/ct2/show/NCT00496626?term=NCT00496626&rank=1) | <http://www.merck.com/clinical-trials/study.html?id=V501-030&kw=gardasil> |
| 21 | ‘Definitely exists’ | V501-030 follow-up | Not identified | NCT01427777 | [https://clinicaltrials.gov/show/NCT01427777](https://clinicaltrials.gov/ct2/show/NCT01427777?term=bivalent+HPV+OR+quadrivalent+HPV+OR+HPV+vaccine+OR+human+papillomavirus+vaccine+OR+Cervarix+OR+Gardasil+OR+Gardasil+4+OR+Gardasil+9&rank=60&submit_fld_opt=) | <http://adisinsight.springer.com/trials/700025686> |
| 22 | ‘Definitely exists’ | V501-031 | 2010_019 | NCT01078220 | [https://clinicaltrials.gov/show/NCT01078220](https://clinicaltrials.gov/ct2/show/NCT01078220?term=bivalent+HPV+OR+quadrivalent+HPV+OR+HPV+vaccine+OR+human+papillomavirus+vaccine+OR+Cervarix+OR+Gardasil+OR+Gardasil+4+OR+Gardasil+9&rank=190&submit_fld_opt=) | <http://www.merck.com/clinical-trials/study.html?id=V501-031&kw=gardasil> |
| 23 | ‘Definitely exists’ | V501-033 | 2010_018 | NCT01077856 | [https://clinicaltrials.gov/show/NCT01077856](https://clinicaltrials.gov/ct2/show/NCT01077856?term=bivalent+HPV+OR+quadrivalent+HPV+OR+HPV+vaccine+OR+human+papillomavirus+vaccine+OR+Cervarix+OR+Gardasil+OR+Gardasil+4+OR+Gardasil+9&rank=160&submit_fld_opt=) | <http://www.merck.com/clinical-trials/study.html?id=V501-033&kw=gardasil> |
| 24 | ‘Definitely exists’ | V501-041 | 2009_532 | NCT00834106 | [https://clinicaltrials.gov/show/NCT00834106](https://clinicaltrials.gov/ct2/show/NCT00834106?term=V501-041&rank=1) | <http://www.merck.com/clinical-trials/study.html?id=V501-041&kw=gardasil> |
| 25 | ‘Definitely exists’ | V501-046 | Not identified | NCT01245764 | [https://clinicaltrials.gov/show/NCT01245764](https://clinicaltrials.gov/ct2/show/NCT01245764?term=V501-046&rank=1) | <http://www.merck.com/clinical-trials/study.html?id=V501-046&kw=gardasil> |
| 26 | ‘Definitely exists’ | V501-070 | Not identified | NCT01567813 | [https://clinicaltrials.gov/show/NCT01567813](https://clinicaltrials.gov/ct2/show/NCT01567813) | <http://www.merck.com/clinical-trials/study.html?id=V501-070&kw=gardasil> |
| 27 | ‘Definitely exists’ | V501-122 | Not identified | NCT01862874 | [https://clinicaltrials.gov/show/NCT01862874](https://clinicaltrials.gov/ct2/show/NCT01862874?term=bivalent+HPV+OR+quadrivalent+HPV+OR+HPV+vaccine+OR+human+papillomavirus+vaccine+OR+Cervarix+OR+Gardasil+OR+Gardasil+4+OR+Gardasil+9&rank=112&submit_fld_opt=) | <http://www.merck.com/clinical-trials/study.html?id=V501-122&kw=gardasil> |
| 28 | ‘Definitely exists’ | V502-001 | 2005_086 | NCT00260039 | [https://clinicaltrials.gov/show/NCT00260039](https://clinicaltrials.gov/ct2/show/NCT00260039) | <http://www.merck.com/clinical-trials/study.html?id=V502-001&kw=gardasil> |
| 29 | ‘Definitely exists’ | V502-002 | 2009_552 | NCT00851643 | [https://clinicaltrials.gov/show/NCT00851643](https://clinicaltrials.gov/ct2/show/NCT00851643?term=bivalent+HPV+OR+quadrivalent+HPV+OR+HPV+vaccine+OR+human+papillomavirus+vaccine+OR+Cervarix+OR+Gardasil+OR+Gardasil+4+OR+Gardasil+9&rank=54&submit_fld_opt=) | <http://www.merck.com/clinical-trials/study.html?id=V502-002&kw=gardasil> |
| 30 | ‘Definitely exists’ | V502-003 | 2006_503 | NCT00365443 | [https://clinicaltrials.gov/show/NCT00365443](https://clinicaltrials.gov/ct2/show/NCT00365443?term=bivalent+HPV+OR+quadrivalent+HPV+OR+HPV+vaccine+OR+human+papillomavirus+vaccine+OR+Cervarix+OR+Gardasil+OR+Gardasil+4+OR+Gardasil+9&rank=252) | <https://www.clinicaltrialsregister.eu/ctr-search/trial/2006-004933-14/DK> |
| 31 | ‘Definitely exists’ | V503-001 | 2007_538 | NCT00543543 | [https://clinicaltrials.gov/show/NCT00543543](https://clinicaltrials.gov/ct2/show/NCT00543543?term=V503-001&rank=2) | <http://www.merck.com/clinical-trials/study.html?id=V503-001&kw=gardasil> |
| 32 | ‘Definitely exists’ | V503-001 follow-up | Not identified | Not identified | <http://www.ins.gob.pe/ensayosclinicos/rpec/recuperarECPBNuevoEN.asp?numec=090-13> | <http://apps.who.int/trialsearch/Trial2.aspx?TrialID=PER-090-13> |
| 33 | ‘Definitely exists’ | V503-002 (immunobridging study) | Not identified | NCT00943722 | <https://clinicaltrials.gov/show/NCT00943722> | <https://www.ncbi.nlm.nih.gov/pmc/articles/PMC4514432/> |
| 34 | ‘Definitely exists’ | V503-002 (lot consistency study) | 2009_611 | NCT00943722 | <https://clinicaltrials.gov/show/NCT00943722> | <https://www.ncbi.nlm.nih.gov/pmc/articles/PMC4514432/> |
| 35 | ‘Definitely exists’ | V503-002 follow-up | Not identified | Not identified | <http://www.ins.gob.pe/ensayosclinicos/rpec/recuperarECPBNuevoEN.asp?numec=120-12> | <http://apps.who.int/trialsearch/Trial2.aspx?TrialID=PER-120-12> |
| 36 | ‘Definitely exists’ | V503-003 | Not identified | NCT01651949 | [https://clinicaltrials.gov/show/NCT01651949](https://clinicaltrials.gov/ct2/show/NCT01651949?term=V503-003&rank=1) | <http://apps.who.int/trialsearch/Trial2.aspx?TrialID=NCT01651949> |
| 37 | ‘Definitely exists’ | V503-004 | Not identified | NCT03158220 | <https://clinicaltrials.gov/show/NCT03158220> | <http://www.merck.com/clinical-trials/study.html?id=V503-004&kw=Gardasil> |
| 38 | ‘Definitely exists’ | V503-005 | Not identified | NCT00988884 | [https://clinicaltrials.gov/show/NCT00988884](https://clinicaltrials.gov/ct2/show/NCT00988884) | <http://pediatrics.aappublications.org/content/136/3/e563.long> |
| 39 | ‘Definitely exists’ | V503-006 | 2010_504 | NCT01047345 | [https://clinicaltrials.gov/show/NCT01047345](https://clinicaltrials.gov/ct2/show/NCT01047345?term=V503-006&rank=1) | <http://www.merck.com/clinical-trials/study.html?id=V503-006&kw=gardasil> |
| 40 | ‘Definitely exists’ | V503-007 | 2010_512 | NCT01073293 | [https://clinicaltrials.gov/show/NCT01073293](https://clinicaltrials.gov/ct2/show/NCT01073293?term=V503-007&rank=1) | <https://www.ncbi.nlm.nih.gov/pubmed/25831420?dopt=Abstract> |
| 41 | ‘Definitely exists’ | V503-008  (V503-002 follow-up) | Not identified | NCT01254643 | [https://clinicaltrials.gov/show/NCT01254643](https://clinicaltrials.gov/ct2/show/NCT01254643?term=V503-008&rank=1) | <http://www.merck.com/clinical-trials/study.html?id=V503-008&kw=vaccines&tab=eligibility> |
| 42 | ‘Definitely exists’ | V503-009 | GDS01C | NCT01304498 | <https://clinicaltrials.gov/show/NCT01304498> | <http://www.merck.com/product/usa/pi_circulars/g/gardasil_9/gardasil_9_pi.pdf> |
| 43 | ‘Definitely exists’ | V503-010 | Not identified | NCT01984697 | [https://clinicaltrials.gov/show/NCT01984697](https://clinicaltrials.gov/ct2/show/NCT01984697?term=V503-010&rank=1) | <https://www.fda.gov/downloads/BiologicsBloodVaccines/Vaccines/ApprovedProducts/UCM524629.pdf> |
| 44 | ‘Probably exists’ | V503-018 | Not identified | Not identified | <http://www.fda.gov/downloads/BiologicsBloodVaccines/Vaccines/ApprovedProducts/UCM190977.pdf> | Not identified |
| 45 | ‘Probably exists’ | V503-019 | Not identified | Not identified | <http://www.fda.gov/downloads/BiologicsBloodVaccines/Vaccines/ApprovedProducts/UCM251763.pdf> | Not identified |
| 46 | ‘Definitely exists’ | V503-020 | GDS07C | NCT02114385 | [https://clinicaltrials.gov/show/NCT02114385](https://clinicaltrials.gov/ct2/show/NCT02114385) | <http://apps.who.int/trialsearch/Trial2.aspx?TrialID=EUCTR2013-003399-10-DE> |
| 47 | ‘Probably exists’ | V503-021  (V503-001 follow-up) | Not identified | NCT02653118 | [https://clinicaltrials.gov/show/NCT02653118](https://clinicaltrials.gov/ct2/show/NCT02653118?term=V503-021&rank=1) | Not identified |
| 48 | ‘Definitely exists’ | V504-001 | 2007_566 | NCT00551187 | [https://clinicaltrials.gov/show/NCT00551187](https://clinicaltrials.gov/ct2/show/NCT00551187?term=bivalent+HPV+OR+quadrivalent+HPV+OR+HPV+vaccine+OR+human+papillomavirus+vaccine+OR+Cervarix+OR+Gardasil+OR+Gardasil+4+OR+Gardasil+9&rank=228) | <http://www.merck.com/clinical-trials/study.html?id=V504-001&kw=gardasil> |
| 49 | ‘Definitely exists’ | V505-001 | 2007_567 | NCT00520598 | [https://clinicaltrials.gov/show/NCT00520598](https://clinicaltrials.gov/ct2/show/NCT00520598?term=bivalent+HPV+OR+quadrivalent+HPV+OR+HPV+vaccine+OR+human+papillomavirus+vaccine+OR+Cervarix+OR+Gardasil+OR+Gardasil+4+OR+Gardasil+9&rank=259) | <http://www.merck.com/clinical-trials/study.html?id=V505-001&kw=gardasil> |
| 50 | ‘Definitely exists’ | Not identified | Not identified | NCT00572832 | [https://clinicaltrials.gov/show/NCT00572832](https://clinicaltrials.gov/ct2/show/NCT00572832?term=bivalent+HPV+OR+quadrivalent+HPV+OR+HPV+vaccine+OR+human+papillomavirus+vaccine+OR+Cervarix+OR+Gardasil+OR+Gardasil+4+OR+Gardasil+9&rank=83) | <https://www.ncbi.nlm.nih.gov/pubmed/20629576?dopt=Abstract> |
| 51 | ‘Definitely exists’ | Not identified | Not identified | NCT00573651 | <https://clinicaltrials.gov/show/NCT00573651> | <http://www.clinicaltrialfacts.com/Arthritis/Safety-and-Efficacy-of-Gardasil-in-Femal-47769> |
| 52 | ‘Definitely exists’ | Not identified | Not identified | NCT00727636 | <https://clinicaltrials.gov/show/NCT00727636> | <http://www.clinicaltrialfacts.com/Inflammatory-Bowel-Disease/Immunogenicity-to-Human-Papillomavirus-V-59478> |
| 53 | ‘Definitely exists’ | Not identified | Not identified | NCT01489527 | [https://clinicaltrials.gov/show/NCT01489527](https://clinicaltrials.gov/ct2/show/NCT01489527?term=NCT01489527&rank=1) | <https://www.ncbi.nlm.nih.gov/pmc/articles/PMC4378717/> |
| 54 | ‘Definitely exists’ | Not identified | Not identified | NCT01924754 | <https://clinicaltrials.gov/show/NCT01924754> | <http://www.clinicaltrialfacts.com/HPV-Seroconversion/Gardasil-Immunogenicity-With-Needle-Free-141969> |
| 55 | ‘Definitely exists’ | Not identified | Not identified | NCT02199691 | [https://clinicaltrials.gov/show/NCT02199691](https://clinicaltrials.gov/ct2/show/NCT02199691) | <https://www.clinicaltrialsregister.eu/ctr-search/trial/2016-001963-35/3rd> |
| 56 | ‘Definitely exists’ | Not identified | Not identified | NCT02968420 | [https://clinicaltrials.gov/show/NCT02968420](https://clinicaltrials.gov/ct2/show/NCT02968420?term=bivalent+HPV+OR+quadrivalent+HPV+OR+HPV+vaccine+OR+human+papillomavirus+vaccine+OR+Cervarix+OR+Gardasil&type=Intr&fund=0123&rcv_s=01%2F01%2F2016&lup_s=01%2F01%2F2016&draw=1&rank=1) | <http://apps.who.int/trialsearch/Trial2.aspx?TrialID=NCT02968420> |
| 57 | ‘Definitely exists’ | Not identified | Not identified | Not identified | <http://apps.who.int/trialsearch/Trial2.aspx?TrialID=KCT0000604> | <http://cris.nih.go.kr/cris/en/search/search_result_st01.jsp?seq=2624> |
| 58 | ‘Probably exists’ | Not identified | Not identified | NCT00767897 | [https://clinicaltrials.gov/show/NCT00767897](https://clinicaltrials.gov/ct2/show/NCT00767897?term=bivalent+HPV+OR+quadrivalent+HPV+OR+HPV+vaccine+OR+human+papillomavirus+vaccine+OR+Cervarix+OR+Gardasil+OR+Gardasil+4+OR+Gardasil+9&rank=260) | Not identified |
| 59 | ‘Probably exists’ | Not identified | Not identified | NCT00806676 | [https://clinicaltrials.gov/show/NCT00806676](https://clinicaltrials.gov/ct2/show/NCT00806676?term=bivalent+HPV+OR+quadrivalent+HPV+OR+HPV+vaccine+OR+human+papillomavirus+vaccine+OR+Cervarix+OR+Gardasil+OR+Gardasil+4+OR+Gardasil+9&rank=131) | Not identified |
| 60 | ‘Probably exists’ | Not identified | Not identified | NCT00925288 | [https://clinicaltrials.gov/show/NCT00925288](https://clinicaltrials.gov/ct2/show/NCT00925288?term=bivalent+HPV+OR+quadrivalent+HPV+OR+HPV+vaccine+OR+human+papillomavirus+vaccine+OR+Cervarix+OR+Gardasil+OR+Gardasil+4+OR+Gardasil+9&rank=30&submit_fld_opt=) | Not identified |
| 61 | ‘Probably exists’ | Not identified | Not identified | NCT00944879 | [https://clinicaltrials.gov/show/NCT00944879](https://clinicaltrials.gov/ct2/show/NCT00944879?term=bivalent+HPV+OR+quadrivalent+HPV+OR+HPV+vaccine+OR+human+papillomavirus+vaccine+OR+Cervarix+OR+Gardasil+OR+Gardasil+4+OR+Gardasil+9&rank=119&submit_fld_opt=) | Not identified |
| 62 | ‘Probably exists’ | Not identified | Not identified | NCT01928225 | [https://clinicaltrials.gov/show/NCT01928225](https://clinicaltrials.gov/ct2/show/NCT01928225?term=bivalent+HPV+OR+quadrivalent+HPV+OR+HPV+vaccine+OR+human+papillomavirus+vaccine+OR+Cervarix+OR+Gardasil+OR+Gardasil+4+OR+Gardasil+9&rank=50&submit_fld_opt=) | Not identified |
| 63 | ‘Probably exists’ | Not identified | Not identified | NCT02382900 | [https://clinicaltrials.gov/show/NCT02382900](https://clinicaltrials.gov/ct2/show/NCT02382900?term=bivalent+HPV+OR+quadrivalent+HPV+OR+HPV+vaccine+OR+human+papillomavirus+vaccine+OR+Cervarix+OR+Gardasil+OR+Gardasil+4+OR+Gardasil+9&rank=142&submit_fld_opt=) | Not identified |
| 64 | ‘Probably exists’ | Not identified | Not identified | NCT02624349 | [https://clinicaltrials.gov/show/NCT02624349](https://clinicaltrials.gov/ct2/show/NCT02624349?term=bivalent+HPV+OR+quadrivalent+HPV+OR+HPV+vaccine+OR+human+papillomavirus+vaccine+OR+Cervarix+OR+Gardasil+OR+Gardasil+4+OR+Gardasil+9&rank=51&submit_fld_opt=) | Not identified |
| 65 | ‘Probably exists’ | Not identified | Not identified | NCT02979535 | <https://clinicaltrials.gov/show/NCT02979535> | Not identified |
| 66 | ‘Probably exists’ | Not identified | Not identified | NCT02993757 | <https://clinicaltrials.gov/show/NCT02993757> | Not identified |

Index of other industry HPV vaccine study programmes:

| **No.** | **Study classification** | **Study programme specific ID** | **Manufacturer specific ID** | **National Clinical Trial (NCT) ID** | **Verification from 1^st^ source** | **Verification from 2^nd^ source** |
| --- | --- | --- | --- | --- | --- | --- |
| **Shanghai Zerun Biotechnology Co., Ltd.** | | | | | | |
| 1 | ‘Definitely exists’ | 311-HPV-1001 | Not identified | NCT03085381 | <https://clinicaltrials.gov/show/NCT03085381> | <http://apps.who.int/trialsearch/Trial2.aspx?TrialID=NCT03085381> |
| 2 | ‘Definitely exists’ | 311-HPV-1002 | Not identified | NCT02740790 | <https://clinicaltrials.gov/show/NCT02740790> | <http://apps.who.int/trialsearch/Trial2.aspx?TrialID=NCT02740790> |
| 3 | ‘Definitely exists’ | 311-HPV-1003 | Not identified | NCT02733068 | <https://clinicaltrials.gov/show/NCT02733068> | <http://apps.who.int/trialsearch/Trial2.aspx?TrialID=NCT02733068> |
| 4 | ‘Definitely exists’ | 311-HPV-1004 | Not identified | NCT02740777 | <https://clinicaltrials.gov/show/NCT02740777> | <http://apps.who.int/trialsearch/Trial2.aspx?TrialID=NCT02740777> |
| **Xiamen Innovax Biotech Co., Ltd.** | | | | | | |
| 5 | ‘Definitely exists’ | HPV-PRO-002 | Not identified | NCT01356823 | [https://clinicaltrials.gov/show/NCT01356823](https://clinicaltrials.gov/ct2/show/NCT01356823) | <https://www.ncbi.nlm.nih.gov/pubmed/26100924?dopt=Abstract> |
| 6 | ‘Definitely exists’ | HPV-PRO-003 | Not identified | NCT01735006 | <https://clinicaltrials.gov/show/NCT01735006> | <http://apps.who.int/trialsearch/Trial2.aspx?TrialID=NCT01735006> |
| 7 | ‘Probably exists’ | HPV-PRO-004 | Not identified | NCT02405520 | [https://clinicaltrials.gov/show/NCT02405520](https://clinicaltrials.gov/ct2/show/NCT02405520?term=HPV-PRO&rank=3) | Not identified |
| 8 | ‘Definitely exists’ | HPV-PRO-005 | Not identified | NCT02710851 | <https://clinicaltrials.gov/show/NCT02710851> | <http://apps.who.int/trialsearch/Trial2.aspx?TrialID=NCT02710851> |
| 9 | ‘Probably exists’ | HPV-PRO-006 | Not identified | NCT02562508 | [https://clinicaltrials.gov/show/NCT02562508](https://clinicaltrials.gov/ct2/show/NCT02562508?term=HPV-PRO&rank=2) | Not identified |
| 10 | ‘Definitely exists’ | HPV-PRO-006 follow-up | Not identified | NCT03206255 | <https://clinicaltrials.gov/show/NCT03206255> | <http://apps.who.int/trialsearch/Trial2.aspx?TrialID=NCT03206255> |

Index of identified HPV vaccine non-industry funded studies

The following is an index of the identified non-industry funded studies:

| **No.** | **Study classification** | **Study ID** | **National Clinical Trial (NCT) ID** | **Verification from 1^st^ source** | **Verification from 2^nd^ source** |
| --- | --- | --- | --- | --- | --- |
| **Non-industry funded Cervarix studies** | | | | | |
| 1 | ‘Definitely exists’ | NL26.113.000.08 | NCT00815282 | [https://clinicaltrials.gov/show/NCT00815282](https://clinicaltrials.gov/ct2/show/NCT00815282?term=bivalent+HPV+OR+quadrivalent+HPV+OR+HPV+vaccine+OR+human+papillomavirus+vaccine+OR+Cervarix+OR+Gardasil+OR+Gardasil+4+OR+Gardasil+9&rank=201&submit_fld_opt=) | <http://ard.bmj.com/content/73/8/1500.long> |
| 2 | ‘Probably exists’ | LIS144 | NCT01082861 | [https://clinicaltrials.gov/show/NCT01082861](https://clinicaltrials.gov/ct2/show/NCT01082861?term=bivalent+HPV+OR+quadrivalent+HPV+OR+HPV+vaccine+OR+human+papillomavirus+vaccine+OR+Cervarix+OR+Gardasil+OR+Gardasil+4+OR+Gardasil+9&rank=264&submit_fld_opt=) | Not identified |
| 3 | ‘Definitely exists’ | Not identified | NCT02276521 | [https://clinicaltrials.gov/show/NCT02276521](https://clinicaltrials.gov/ct2/show/NCT02276521?term=bivalent+HPV+OR+quadrivalent+HPV+OR+HPV+vaccine+OR+human+papillomavirus+vaccine+OR+Cervarix+OR+Gardasil+OR+Gardasil+4+OR+Gardasil+9&rank=98) | <https://academic.oup.com/cid/article/doi/10.1093/cid/ciw865/2747464/Sustained-Antibody-Responses-6-Years-Following-1-2> |
| 4 | ‘Definitely exists’ | 342/2009 | NCT02296255 | [https://clinicaltrials.gov/show/NCT02296255](https://clinicaltrials.gov/ct2/show/NCT02296255?term=bivalent+HPV+OR+quadrivalent+HPV+OR+HPV+vaccine+OR+human+papillomavirus+vaccine+OR+Cervarix+OR+Gardasil+OR+Gardasil+4+OR+Gardasil+9&rank=118&submit_fld_opt=) | <http://apps.who.int/trialsearch/Trial2.aspx?TrialID=NCT02296255> |
| 5 | ‘Definitely exists’ | FASTER-Tialpan Study | NCT03105856 | <https://clinicaltrials.gov/show/NCT03105856> | <http://apps.who.int/trialsearch/Trial2.aspx?TrialID=NCT03105856> |
| 6 | ‘Definitely exists’ | ESCUDDO | NCT03180034 | <https://clinicaltrials.gov/show/NCT03180034> | <http://apps.who.int/trialsearch/Trial2.aspx?TrialID=NCT03180034> |
| **Non-industry funded Gardasil and Gardasil 9 studies** | | | | | |
| 7 | ‘Definitely exists’ | P1047 | NCT00339040 | [https://clinicaltrials.gov/show/NCT00339040](https://clinicaltrials.gov/ct2/show/NCT00339040?term=bivalent+HPV+OR+quadrivalent+HPV+OR+HPV+vaccine+OR+human+papillomavirus+vaccine+OR+Cervarix+OR+Gardasil+OR+Gardasil+4+OR+Gardasil+9&rank=205&submit_fld_opt=) | <https://www.ncbi.nlm.nih.gov/pmc/articles/PMC3033215/> |
| 8 | ‘Definitely exists’ | H07-00928 | NCT00501137 | [https://clinicaltrials.gov/show/NCT00501137](https://clinicaltrials.gov/ct2/show/NCT00501137?term=bivalent+HPV+OR+quadrivalent+HPV+OR+HPV+vaccine+OR+human+papillomavirus+vaccine+OR+Cervarix+OR+Gardasil+OR+Gardasil+4+OR+Gardasil+9&rank=29&submit_fld_opt=) | <http://jamanetwork.com/journals/jama/fullarticle/1682939> |
| 9 | ‘Definitely exists’ | DDEAMC-07-43X | NCT00501189 | [https://clinicaltrials.gov/show/NCT00501189](https://clinicaltrials.gov/ct2/show/NCT00501189?term=bivalent+HPV+OR+quadrivalent+HPV+OR+HPV+vaccine+OR+human+papillomavirus+vaccine+OR+Cervarix+OR+Gardasil+OR+Gardasil+4+OR+Gardasil+9&rank=87) | <http://www.clinicaltrialfacts.com/Papillomavirus-Infections/Gardasil-Vaccination-as-Therapy-in-Low-G-42300> |
| 10 | ‘Definitely exists’ | HPV01 | NCT00524745 | [https://clinicaltrials.gov/show/NCT00524745](https://clinicaltrials.gov/ct2/show/NCT00524745?term=bivalent+HPV+OR+quadrivalent+HPV+OR+HPV+vaccine+OR+human+papillomavirus+vaccine+OR+Cervarix+OR+Gardasil+OR+Gardasil+4+OR+Gardasil+9&rank=134) | <http://jid.oxfordjournals.org/content/208/8/1325.long> |
| 11 | ‘Definitely exists’ | A5240 | NCT00604175 | [https://clinicaltrials.gov/show/NCT00604175](https://clinicaltrials.gov/ct2/show/NCT00604175?term=bivalent+HPV+OR+quadrivalent+HPV+OR+HPV+vaccine+OR+human+papillomavirus+vaccine+OR+Cervarix+OR+Gardasil+OR+Gardasil+4+OR+Gardasil+9&rank=26&submit_fld_opt=) | <https://www.ncbi.nlm.nih.gov/pmc/articles/PMC4305143/> |
| 12 | ‘Probably exists’ | ATN064 | NCT00710593 | [https://clinicaltrials.gov/show/NCT00710593](https://clinicaltrials.gov/ct2/show/NCT00710593?term=bivalent+HPV+OR+quadrivalent+HPV+OR+HPV+vaccine+OR+human+papillomavirus+vaccine+OR+Cervarix+OR+Gardasil+OR+Gardasil+4+OR+Gardasil+9&rank=126) | Not identified |
| 13 | ‘Probably exists’ | 090024 | NCT00798265 | [https://clinicaltrials.gov/show/NCT00798265](https://clinicaltrials.gov/ct2/show/NCT00798265?term=bivalent+HPV+OR+quadrivalent+HPV+OR+HPV+vaccine+OR+human+papillomavirus+vaccine+OR+Cervarix+OR+Gardasil+OR+Gardasil+4+OR+Gardasil+9&rank=267&submit_fld_opt=) | Not identified |
| 14 | ‘Definitely exists’ | Pro00014388 | NCT00862810 | [https://clinicaltrials.gov/show/NCT00862810](https://clinicaltrials.gov/ct2/show/NCT00862810?term=bivalent+HPV+OR+quadrivalent+HPV+OR+HPV+vaccine+OR+human+papillomavirus+vaccine+OR+Cervarix+OR+Gardasil+OR+Gardasil+4+OR+Gardasil+9&rank=28&submit_fld_opt=) | <http://jamanetwork.com/journals/jama/fullarticle/1682939> |
| 15 | ‘Definitely exists’ | Not identified | NCT00923702 | [https://clinicaltrials.gov/show/NCT00923702](https://clinicaltrials.gov/ct2/show/NCT00923702?term=bivalent+HPV+OR+quadrivalent+HPV+OR+HPV+vaccine+OR+human+papillomavirus+vaccine+OR+Cervarix+OR+Gardasil+OR+Gardasil+4+OR+Gardasil+9&rank=137) | [https://www.isrctn.com/ISRCTN98283094](https://www.isrctn.com/ISRCTN98283094?q=Gardasil&filters=&sort=&offset=3&totalResults=3&page=1&pageSize=10&searchType=basic-search) |
| 16 | ‘Definitely exists’ | HRPO-07-0648 | NCT00941889 | [https://clinicaltrials.gov/show/NCT00941889](https://clinicaltrials.gov/ct2/show/NCT00941889?term=bivalent+HPV+OR+quadrivalent+HPV+OR+HPV+vaccine+OR+human+papillomavirus+vaccine+OR+Cervarix+OR+Gardasil+OR+Gardasil+4+OR+Gardasil+9&rank=92) | <http://www.clinicaltrialfacts.com/HIV-Positive/The-Effect-of-HPV-Vaccination-on-Recurre-75760> |
| 17 | ‘Definitely exists’ | SG09-EN01 | NCT00949572 | [https://clinicaltrials.gov/show/NCT00949572](https://clinicaltrials.gov/ct2/show/NCT00949572?term=bivalent+HPV+OR+quadrivalent+HPV+OR+HPV+vaccine+OR+human+papillomavirus+vaccine+OR+Cervarix+OR+Gardasil+OR+Gardasil+4+OR+Gardasil+9&rank=236) | <https://www.ncbi.nlm.nih.gov/pmc/articles/PMC3306286/> |
| 18 | ‘Definitely exists’ | HPV CSP01 | NCT00956553 | [https://clinicaltrials.gov/show/NCT00956553](https://clinicaltrials.gov/ct2/show/NCT00956553?term=bivalent+HPV+OR+quadrivalent+HPV+OR+HPV+vaccine+OR+human+papillomavirus+vaccine+OR+Cervarix+OR+Gardasil+OR+Gardasil+4+OR+Gardasil+9&rank=37&submit_fld_opt=) | <http://www.clinicaltrialfacts.com/HPV-Infections/Reactogenicity-Study-of-Cervarix-and-Gar-76885> |
| 19 | ‘Probably exists’ | 08-0012-N01AI80006C | NCT01030562 | [https://clinicaltrials.gov/show/NCT01030562](https://clinicaltrials.gov/ct2/show/NCT01030562?term=bivalent+HPV+OR+quadrivalent+HPV+OR+HPV+vaccine+OR+human+papillomavirus+vaccine+OR+Cervarix+OR+Gardasil+OR+Gardasil+4+OR+Gardasil+9&rank=207&submit_fld_opt=) | Not identified |
| 20 | ‘Definitely exists’ | MITU-001 | NCT01173900 | [https://clinicaltrials.gov/show/NCT01173900](https://clinicaltrials.gov/ct2/show/NCT01173900?term=bivalent+HPV+OR+quadrivalent+HPV+OR+HPV+vaccine+OR+human+papillomavirus+vaccine+OR+Cervarix+OR+Gardasil+OR+Gardasil+4+OR+Gardasil+9&rank=185&submit_fld_opt=) | <https://www.ncbi.nlm.nih.gov/pubmed/22711908> |
| 21 | ‘Definitely exists’ | LTN0001 | NCT01386164 | [https://clinicaltrials.gov/show/NCT01386164](https://clinicaltrials.gov/ct2/show/NCT01386164?term=bivalent+HPV+OR+quadrivalent+HPV+OR+HPV+vaccine+OR+human+papillomavirus+vaccine+OR+Cervarix+OR+Gardasil+OR+Gardasil+4+OR+Gardasil+9&rank=24&submit_fld_opt=) | <https://www.ncbi.nlm.nih.gov/pmc/articles/PMC4896591/> |
| 22 | ‘Definitely exists’ | 9427-L1802/1-21C | NCT01456715 | [https://clinicaltrials.gov/show/NCT01456715](https://clinicaltrials.gov/ct2/show/NCT01456715?term=bivalent+HPV+OR+quadrivalent+HPV+OR+HPV+vaccine+OR+human+papillomavirus+vaccine+OR+Cervarix+OR+Gardasil+OR+Gardasil+4+OR+Gardasil+9&rank=4&submit_fld_opt=) | <http://www.clinicaltrialfacts.com/Human-Papillomavirus/Immunogenicity-of-Gardasil-and-Twinrix-a-115114> |
| 23 | ‘Definitely exists’ | A5298 | NCT01461096 | [https://clinicaltrials.gov/show/NCT01461096](https://clinicaltrials.gov/ct2/show/NCT01461096?term=bivalent+HPV+OR+quadrivalent+HPV+OR+HPV+vaccine+OR+human+papillomavirus+vaccine+OR+Cervarix+OR+Gardasil+OR+Gardasil+4+OR+Gardasil+9&rank=9&submit_fld_opt=) | <http://www.clinicaltrialfacts.com/HIV-Infections/Evaluating-the-Effectiveness-of-the-Quad-115451> |
| 24 | ‘Probably exists’ | IRB00046117 | NCT01505049 | [https://clinicaltrials.gov/show/NCT01505049](https://clinicaltrials.gov/ct2/show/NCT01505049?term=bivalent+HPV+OR+quadrivalent+HPV+OR+HPV+vaccine+OR+human+papillomavirus+vaccine+OR+Cervarix+OR+Gardasil+OR+Gardasil+4+OR+Gardasil+9&rank=14&submit_fld_opt=) | Not identified |
| 25 | ‘Definitely exists’ | HLS04/2011 | NCT01512784 | [https://clinicaltrials.gov/show/NCT01512784](https://clinicaltrials.gov/ct2/show/NCT01512784?term=bivalent+HPV+OR+quadrivalent+HPV+OR+HPV+vaccine+OR+human+papillomavirus+vaccine+OR+Cervarix+OR+Gardasil+OR+Gardasil+4+OR+Gardasil+9&rank=6&submit_fld_opt=) | <http://www.clinicaltrialfacts.com/HPV/Long-Term-Immunogenicity-of-Quadrivalent-119444> |
| 26 | ‘Definitely exists’ | 883 | NCT01717118 | <https://clinicaltrials.gov/show/NCT01717118> | <http://www.tandfonline.com/doi/full/10.1080/21645515.2015.1058458?scroll=top&needAccess=true> |
| 27 | ‘Probably exists’ | CIHR-MOP-125949 | NCT01824537 | <https://clinicaltrials.gov/show/NCT01824537> | Not identified |
| 28 | ‘Probably exists’ | HPV girls 5 year follow up | NCT01896986 | [https://clinicaltrials.gov/show/NCT01896986](https://clinicaltrials.gov/ct2/show/NCT01896986?term=bivalent+HPV+OR+quadrivalent+HPV+OR+HPV+vaccine+OR+human+papillomavirus+vaccine+OR+Cervarix+OR+Gardasil+OR+Gardasil+4+OR+Gardasil+9&rank=164) | Not identified |
| 29 | ‘Probably exists’ | 2013/422 | NCT01914367 | [https://clinicaltrials.gov/show/NCT01914367](https://clinicaltrials.gov/ct2/show/NCT01914367?term=bivalent+HPV+OR+quadrivalent+HPV+OR+HPV+vaccine+OR+human+papillomavirus+vaccine+OR+Cervarix+OR+Gardasil+OR+Gardasil+4+OR+Gardasil+9&rank=3&submit_fld_opt=) | Not identified |
| 30 | ‘Probably exists’ | ICI-VPH-1 | NCT02009800 | [https://clinicaltrials.gov/show/NCT02009800](https://clinicaltrials.gov/ct2/show/NCT02009800?term=bivalent+HPV+OR+quadrivalent+HPV+OR+HPV+vaccine+OR+human+papillomavirus+vaccine+OR+Cervarix+OR+Gardasil+OR+Gardasil+4+OR+Gardasil+9&rank=44&submit_fld_opt=) | Not identified |
| 31 | ‘Definitely exists’ | NL45200.018.13 | NCT02087384 | [https://clinicaltrials.gov/show/NCT02087384](https://clinicaltrials.gov/ct2/show/NCT02087384?term=bivalent+HPV+OR+quadrivalent+HPV+OR+HPV+vaccine+OR+human+papillomavirus+vaccine+OR+Cervarix+OR+Gardasil+OR+Gardasil+4+OR+Gardasil+9&rank=21&submit_fld_opt=) | <http://www.clinicaltrialfacts.com/AIN/HPV-Human-Papilloma-Virus-Vaccination--147051> |
| 32 | ‘Probably exists’ | Pro00014388_1 | NCT02280642 | [https://clinicaltrials.gov/show/NCT02280642](https://clinicaltrials.gov/ct2/show/NCT02280642?term=bivalent+HPV+OR+quadrivalent+HPV+OR+HPV+vaccine+OR+human+papillomavirus+vaccine+OR+Cervarix+OR+Gardasil+OR+Gardasil+4+OR+Gardasil+9&rank=129) | Not identified |
| 33 | ‘Probably exists’ | GINI Study | NCT02363660 | [https://clinicaltrials.gov/show/NCT02363660](https://clinicaltrials.gov/ct2/show/NCT02363660?term=bivalent+HPV+OR+quadrivalent+HPV+OR+HPV+vaccine+OR+human+papillomavirus+vaccine+OR+Cervarix+OR+Gardasil+OR+Gardasil+4+OR+Gardasil+9&rank=262&submit_fld_opt=) | Not identified |
| 34 | ‘Probably exists’ | NTWC/CREC/15035 | NCT02477254 | [https://clinicaltrials.gov/show/NCT02477254](https://clinicaltrials.gov/ct2/show/NCT02477254?term=bivalent+HPV+OR+quadrivalent+HPV+OR+HPV+vaccine+OR+human+papillomavirus+vaccine+OR+Cervarix+OR+Gardasil+OR+Gardasil+4+OR+Gardasil+9&rank=124) | Not identified |
| 35 | ‘Probably exists’ | HPV 2355 | NCT02567955 | [https://clinicaltrials.gov/show/NCT02567955](https://clinicaltrials.gov/ct2/show/NCT02567955?term=bivalent+HPV+OR+quadrivalent+HPV+OR+HPV+vaccine+OR+human+papillomavirus+vaccine+OR+Cervarix+OR+Gardasil+OR+Gardasil+4+OR+Gardasil+9&rank=1&submit_fld_opt=) | Not identified |
| 36 | ‘Definitely exists’ | MISP-53183 | NCT02750202 | [https://clinicaltrials.gov/show/NCT02750202](https://clinicaltrials.gov/ct2/show/NCT02750202?term=bivalent+HPV+OR+quadrivalent+HPV+OR+HPV+vaccine+OR+human+papillomavirus+vaccine+OR+Cervarix+OR+Gardasil+OR+Gardasil+4+OR+Gardasil+9&rank=5&submit_fld_opt=) | <http://apps.who.int/trialsearch/Trial2.aspx?TrialID=NCT02750202> |
| 37 | ‘Definitely exists’ | MITU-002 | NCT02834637 | [https://clinicaltrials.gov/show/NCT02834637](https://clinicaltrials.gov/ct2/show/NCT02834637?term=bivalent+HPV+OR+quadrivalent+HPV+OR+HPV+vaccine+OR+human+papillomavirus+vaccine+OR+Cervarix+OR+Gardasil+OR+Gardasil+4+OR+Gardasil+9&rank=2&submit_fld_opt=) | <http://apps.who.int/trialsearch/Trial2.aspx?TrialID=NCT02834637> |
| 38 | ‘Probably exists’ | 1603017415 | NCT02864147 | [https://clinicaltrials.gov/show/NCT02864147](https://clinicaltrials.gov/ct2/show/NCT02864147?term=bivalent+HPV+OR+quadrivalent+HPV+OR+HPV+vaccine+OR+human+papillomavirus+vaccine+OR+Cervarix+OR+Gardasil+OR+Gardasil+4+OR+Gardasil+9&rank=125) | Not identified |
| 39 | ‘Probably exists’ | cycdc2016-4 | NCT02888418 | [https://clinicaltrials.gov/show/NCT02888418](https://clinicaltrials.gov/ct2/show/NCT02888418?term=bivalent+HPV+OR+quadrivalent+HPV+OR+HPV+vaccine+OR+human+papillomavirus+vaccine+OR+Cervarix+OR+Gardasil+OR+Gardasil+4+OR+Gardasil+9&rank=39&submit_fld_opt=) | Not identified |
| 40 | ‘Definitely exists’ | Not identified | Not identified | <https://www.clinicaltrialsregister.eu/ctr-search/trial/2016-002083-13/GB> | <http://apps.who.int/trialsearch/Trial2.aspx?TrialID=EUCTR2016-002083-13-GB> |
| 41 | ‘Definitely exists’ | ACTRN12608000339358 | Not identified | <http://apps.who.int/trialsearch/Trial2.aspx?TrialID=ACTRN12608000339358> | <https://www.anzctr.org.au/Trial/Registration/TrialReview.aspx?ACTRN=12608000339358> |
| 42 | ‘Probably exists’ | ACTRN12613001207707 | Not identified | <https://www.anzctr.org.au/Trial/Registration/TrialReview.aspx?id=364244&isReview=true> | Not identified |
| 43 | ‘Probably exists’ | CTRI/2013/11/004140 | Not identified | <http://ctri.nic.in/Clinicaltrials/pmaindet2.php?trialid=5969&EncHid=&userName=papillomavirus> | Not identified |
| 44 | ‘Definitely exists’ | IPAR0001 | Not identified | <http://apps.who.int/trialsearch/Trial2.aspx?TrialID=EUCTR2012-000445-12-DK> | <https://www.clinicaltrialsregister.eu/ctr-search/trial/2012-000445-12/DK> |
| 45 | ‘Probably exists’ | Not identified | Not identified | [https://www.isrctn.com/ISRCTN14732216](https://www.isrctn.com/ISRCTN14732216?q=HPV vaccine&filters=&sort=&offset=4&totalResults=15&page=1&pageSize=10&searchType=basic-search) | Not identified |
| 46 | ‘Definitely exists’ | Not identified | Not identified | <http://www.isrctn.com/ISRCTN14732216> | <http://apps.who.int/trialsearch/Trial2.aspx?TrialID=ISRCTN14732216> |
| 47 | ‘Definitely exists’ | Not identified | Not identified | <https://www.isrctn.com/ISRCTN32729817> | <http://apps.who.int/trialsearch/Trial2.aspx?TrialID=ISRCTN32729817> |
| 48 | ‘Definitely exists’ | LTN0001 | Not identified | <http://apps.who.int/trialsearch/Trial2.aspx?TrialID=EUCTR2011-001871-37-DK> | <https://www.clinicaltrialsregister.eu/ctr-search/trial/2011-001871-37/DK> |
| 49 | ‘Definitely exists’ | 2010-1090 | Not identified | <http://apps.who.int/trialsearch/Trial2.aspx?TrialID=EUCTR2012-004007-13-DE> | <https://www.clinicaltrialsregister.eu/ctr-search/trial/2012-004007-13/DE> |
| 50 | ‘Definitely exists’ | P150957 | Not identified | <https://www.clinicaltrialsregister.eu/ctr-search/trial/2016-002455-20/FR> | <http://apps.who.int/trialsearch/Trial2.aspx?TrialID=EUCTR2016-002455-20-FR> |
| 51 | ‘Definitely exists’ | VACCAIN-P | Not identified | <http://apps.who.int/trialsearch/Trial2.aspx?TrialID=EUCTR2013-002009-70-NL> | <https://www.clinicaltrialsregister.eu/ctr-search/trial/2013-002009-70/NL> |
| 52 | ‘Probably exists’ | Not identified | Not identified | <http://www.tandfonline.com/doi/full/10.1080/21645515.2016.1277846?scroll=top&needAccess=true> | Not identified |
| 53 | ‘Probably exists’ | Not identified | Not identified | <http://www.sciencedirect.com/science/article/pii/S0264410X13008128> | Not identified |
| 54 | ‘Probably exists’ | Not identified | Not identified | <http://online.liebertpub.com/doi/abs/10.1089/jwh.2009.1753> | Not identified |
| **Other non-industry funded HPV vaccine studies** | | | | | |
| 55 | ‘Probably exists’ | 999909106 | NCT00867464 | [https://clinicaltrials.gov/show/NCT00867464](https://clinicaltrials.gov/ct2/show/NCT00867464?term=bivalent+HPV+OR+quadrivalent+HPV+OR+HPV+vaccine+OR+human+papillomavirus+vaccine+OR+Cervarix+OR+Gardasil+OR+Gardasil+4+OR+Gardasil+9&rank=304) | Not identified |
| 56 | ‘Probably exists’ | HP-00041372 | NCT00257738 | [https://clinicaltrials.gov/show/NCT00257738](https://clinicaltrials.gov/ct2/show/NCT00257738?term=bivalent+HPV+OR+quadrivalent+HPV+OR+HPV+vaccine+OR+human+papillomavirus+vaccine+OR+Cervarix+OR+Gardasil+OR+Gardasil+4+OR+Gardasil+9&rank=336&submit_fld_opt=) | Not identified |
| 57 | ‘Probably exists’ | HP-41372 | NCT00704041 | [https://clinicaltrials.gov/show/NCT00704041](https://clinicaltrials.gov/ct2/show/NCT00704041?term=bivalent+HPV+OR+quadrivalent+HPV+OR+HPV+vaccine+OR+human+papillomavirus+vaccine+OR+Cervarix+OR+Gardasil+OR+Gardasil+4+OR+Gardasil+9&rank=334&submit_fld_opt=) | Not identified |
| 58 | ‘Probably exists’ | CDR0000383786 | NCT00091130 | [https://clinicaltrials.gov/show/NCT00091130](https://clinicaltrials.gov/ct2/show/NCT00091130?term=bivalent+HPV+OR+quadrivalent+HPV+OR+HPV+vaccine+OR+human+papillomavirus+vaccine+OR+Cervarix+OR+Gardasil+OR+Gardasil+4+OR+Gardasil+9&rank=308) | Not identified |
| 59 | ‘Probably exists’ | PE1201 | NCT02564237 | [https://clinicaltrials.gov/show/NCT02564237](https://clinicaltrials.gov/ct2/show/NCT02564237?term=bivalent+HPV+OR+quadrivalent+HPV+OR+HPV+vaccine+OR+human+papillomavirus+vaccine+OR+Cervarix+OR+Gardasil+OR+Gardasil+4+OR+Gardasil+9&rank=221) | Not identified |
| 60 | ‘Probably exists’ | DRKS00005278 | Not identified | <https://www.drks.de/drks_web/navigate.do?navigationId=trial.HTML&TRIAL_ID=DRKS00005278> | Not identified |
| 61 | ‘Probably exists’ | Not identified | Not identified | <http://jnci.oxfordjournals.org/content/93/4/284.long> | Not identified |

Index of identified industry and non-industry funded publications in the HPV vaccine study programmes

The following is a list of identified journal publications of industry and non-industry funded studies:

| **Identified journal publications of industry funded studies** | | | | |
| --- | --- | --- | --- | --- |
| **No.** | **Study programme specific ID** | **Manufacturer specific ID** | **National Clinical Trial (NCT) ID** | **Publication source** |
| 1 | HPV-001 | 580299/001 | NCT00689741 | <http://www.thelancet.com/journals/lancet/article/PIIS0140-6736(04)17398-4/abstract> |
| 2 | HPV-001-NG-PRI | 109836 | NCT00478621 | <http://www.sciencedirect.com/science/article/pii/S0264410X14004095> |
| 3 | HPV-004 | 580299/004 | NCT00693615 | <http://www.sciencedirect.com/science/article/pii/S0264410X06007092> |
| 4 | HPV-005 | 580299/005 | NCT00693966 | <http://www.sciencedirect.com/science/article/pii/S0264410X06007092> |
| 5 | HPV-007 (HPV-001 follow-up) | 580299/007 | NCT00120848 | <http://www.thelancet.com/journals/lancet/article/PIIS0140-6736(09)61567-1/abstract> |
| 6 | HPV-008 | 580299/008 | NCT00122681 | <http://www.thelancet.com/journals/lancet/article/PIIS0140-6736(09)61248-4/abstract> |
| 7 | HPV-009 | 580299/009 | NCT00128661 | <http://www.sciencedirect.com/science/article/pii/S0002937816003094> |
| 8 | HPV-010 | 108933 | NCT00423046 | <https://www.ncbi.nlm.nih.gov/pmc/articles/PMC3338932/> |
| 9 | HPV-011 | 580299/011 | NCT00309166 | <http://www.jahonline.org/article/S1054-139X(08)00434-5/pdf> |
| 10 | HPV-012 | 107479 | NCT00337818 | <http://onlinelibrary.wiley.com/doi/10.1002/ijc.25887/full> |
| 11 | HPV-013 | 104904 | NCT00196924 | <http://www.jahonline.org/article/S1054-139X(10)00102-3/abstract> |
| 12 | HPV-013 follow-up | 104896 | NCT00316706 | <http://www.jahonline.org/article/S1054-139X(11)00615-X/abstract> |
| 13 | HPV-014 | 103514 | NCT00196937 | <http://www.sciencedirect.com/science/article/pii/S0264410X08015004> |
| 14 | HPV-015 | 104820 | NCT00294047 | <http://www.thelancet.com/journals/lancet/article/PIIS0140-6736(14)60920-X/abstract> |
| 15 | HPV-018 | 107682 | NCT00369824 | <https://www.ncbi.nlm.nih.gov/pubmed/21817954> |
| 16 | HPV-020 | 107863 | NCT00586339 | <http://www.sciencedirect.com/science/article/pii/S0264410X13012735> |
| 17 | HPV-021 | 106069 | NCT00481767 | <https://www.ncbi.nlm.nih.gov/pmc/articles/PMC3636781/> |
| 18 | HPV-023 (HPV-001 follow-up) | 109624 | NCT00518336 | <https://www.ncbi.nlm.nih.gov/pmc/articles/PMC4896780/> |
| 19 | HPV-025 (HPV-013 follow-up) | 111375 | NCT00877877 | <http://adisinsight.springer.com/trials/700229791> |
| 20 | HPV-026 | 111567 | NCT00637195 | <https://www.ncbi.nlm.nih.gov/pmc/articles/PMC3165228/> |
| 21 | HPV-029 | 110886 | NCT00578227 | <http://www.jahonline.org/article/S1054-139X(11)00353-3/pdf> |
| 22 | HPV-030 | 111507 | NCT00652938 | <http://www.sciencedirect.com/science/article/pii/S0264410X11012680> |
| 23 | HPV-031 | 104479 | NCT00344032 | <http://onlinelibrary.wiley.com/doi/10.1111/j.1447-0756.2009.01167.x/abstract> |
| 24 | HPV-032 | 104798 | NCT00316693 | <https://www.ncbi.nlm.nih.gov/pubmed/20606533> |
| 25 | HPV-033 | 104951 | NCT00290277 | <http://jkms.org/DOIx.php?id=10.3346/jkms.2010.25.8.1197> |
| 26 | HPV-035 | 106001 | NCT00306241 | <http://www.hkmj.org/abstracts/v16n3/171.htm> |
| 27 | HPV-036 | 105926 | NCT00345878 | <http://www.e-mjm.org/2014/v69n1/cervical-cancer-vaccine.pdf> |
| 28 | HPV-038 | 107291 | NCT00485732 | <https://www.ncbi.nlm.nih.gov/pmc/articles/PMC3152758/> |
| 29 | HPV-039 | 107638 | NCT00779766 | <https://www.ncbi.nlm.nih.gov/pmc/articles/PMC4277330/> |
| 30 | HPV-040 | 106636 | NCT00534638 | <http://www.sciencedirect.com/science/article/pii/S0264410X1401648X> |
| 31 | HPV-042 | 108464 | NCT00426361 | <http://www.jahonline.org/article/S1054-139X(09)00629-6/abstract> |
| 32 | HPV-044 | 109179 | NCT00552279 | <https://www.ncbi.nlm.nih.gov/pubmed/21273939?dopt=Abstract> |
| 33 | HPV-048 | 110659 | NCT00541970 | <https://www.ncbi.nlm.nih.gov/pmc/articles/PMC3338934/> |
| 34 | HPV-051 | 102115 | NCT00231413 | <http://www.sciencedirect.com/science/article/pii/S0264410X14004095> |
| 35 | HPV-058 (HPV-069 co-study) | 112022 | NCT00996125 | <https://www.ncbi.nlm.nih.gov/pmc/articles/PMC4186032/> |
| 36 | HPV-060 (HPV-013 follow-up) | 112772 | NCT00947115 | <https://www.ncbi.nlm.nih.gov/pmc/articles/PMC4489326/> |
| 37 | HPV-063 (HPV-032 follow-up) | 112949 | NCT00929526 | <https://www.ncbi.nlm.nih.gov/pmc/articles/PMC4186043/> |
| 38 | HPV-068 (HPV-023 follow-up) | 114379 | NCT01418937 | <http://adisinsight.springer.com/trials/700228771> |
| 39 | HPV-069 (HPV-058 co-study) | 114590 | NCT01277042 | <https://www.ncbi.nlm.nih.gov/pmc/articles/PMC4186032/> |
| 40 | HPV-070 | 114700 | NCT01381575 | <https://academic.oup.com/jid/article/215/11/1711/3862470/Sustained-Immunogenicity-of-2-dose-Human> |
| 41 | HPV-071 | 115411 | NCT01462357 | <https://www.ncbi.nlm.nih.gov/pmc/articles/PMC4514190/> |
| 42 | HPV-PRO-002 | Not identified | NCT01356823 | <https://www.ncbi.nlm.nih.gov/pubmed/26100924?dopt=Abstract> |
| 43 | V501-005 | 2006_515 | NCT00365378 | <http://www.thelancet.com/journals/lancet/article/PIIS0140-6736(07)60852-6/abstract> |
| 44 | V501-005 follow-up | Not identified | Not identified | [https://www.ncbi.nlm.nih.gov/pmc/articles/PMC2749988/#R10](https://www.ncbi.nlm.nih.gov/pmc/articles/PMC2749988/#R10) |
| 45 | V501-007 | 2006_516 | NCT00365716 | <https://www.ncbi.nlm.nih.gov/pubmed/15863374?dopt=Abstract> |
| 46 | V501-011 | 2007_576 | NCT00517309 | <https://www.ncbi.nlm.nih.gov/pubmed/18164106?dopt=Abstract> |
| 47 | V501-012 | 2004_080 | NCT00092482 | <https://www.ncbi.nlm.nih.gov/pmc/articles/PMC1951095/> |
| 48 | V501-013 | 2004_081 | NCT00092521 | [http://www.nejm.org/doi/full/10.1056/NEJMoa061760#t=article](http://www.nejm.org/doi/full/10.1056/NEJMoa061760#t=article) |
| 49 | V501-015 | 2004_082 | NCT00092534 | <http://www.thelancet.com/journals/lancet/article/PIIS0140-6736(07)60852-6/abstract> |
| 50 | V501-018 | 2004_084 | NCT00092547 | <http://pediatrics.aappublications.org/content/134/3/e657.long> |
| 51 | V501-019 | 2004_013 | NCT00090220 | <http://www.thelancet.com/journals/lancet/article/PIIS0140-6736(09)60691-7/abstract> |
| 52 | V501-020 | 2004_103 | NCT00090285 | <http://www.nejm.org/doi/full/10.1056/NEJMoa0909537> |
| 53 | V501-023 | 2005_066 | NCT00157950 | <https://www.ncbi.nlm.nih.gov/pubmed/17986242?dopt=Abstract> |
| 54 | V501-024 | 2005_093 | NCT00337428 | <https://www.ncbi.nlm.nih.gov/pubmed/19952980?dopt=Abstract> |
| 55 | V501-025 | 2005_092 | NCT00325130 | <https://www.ncbi.nlm.nih.gov/pubmed/19952980?dopt=Abstract> |
| 56 | V501-027 | 2006_032 | NCT00378560 | <http://onlinelibrary.wiley.com/doi/10.1111/cas.12106/abstract> |
| 57 | V501-030 | 2007_021 | NCT00496626 | <https://www.ncbi.nlm.nih.gov/pubmed/22433961> |
| 58 | V501-031 | 2010_019 | NCT01078220 | <http://jamanetwork.com/journals/jamapediatrics/fullarticle/1363509> |
| 59 | V501-033 | 2010_018 | NCT01077856 | <http://jnci.oxfordjournals.org/content/106/3/djt460.long> |
| 60 | V501-046 | Not identified | NCT01245764 | <https://www.ncbi.nlm.nih.gov/pmc/articles/PMC4514396/> |
| 61 | V502-001 | 2005_086 | NCT00260039 | <https://www.ncbi.nlm.nih.gov/pmc/articles/PMC4514333/> |
| 62 | V502-002 | 2009_552 | NCT00851643 | <http://adisinsight.springer.com/trials/700042113> |
| 63 | V503-001 | 2007_538 | NCT00543543 | <http://www.nejm.org/doi/full/10.1056/NEJMoa1405044> |
| 64 | V503-002 (immunobridging study) | Not identified | NCT00943722 | <http://pediatrics.aappublications.org/content/136/1/e28> |
| 65 | V503-002 (lot consistency study) | 2009_611 | NCT00943722 | <https://www.ncbi.nlm.nih.gov/pmc/articles/PMC4514432/> |
| 66 | V503-003 | 2012-002758-22 | NCT01651949 | <https://www.ncbi.nlm.nih.gov/pubmed/26144901?dopt=Abstract> |
| 67 | V503-005 | Not identified | NCT00988884 | <http://pediatrics.aappublications.org/content/136/3/e563.long> |
| 68 | V503-006 | 2010_504 | NCT01047345 | <http://www.sciencedirect.com/science/article/pii/S0264410X15011871> |
| 69 | V503-007 | 2010_512 | NCT01073293 | <https://www.ncbi.nlm.nih.gov/pubmed/25831420?dopt=Abstract> |
| 70 | V503-008 (V503-002 follow-up) | Not identified | NCT01254643 | [http://198.61.244.207/Upload/85_Applied%20Redaction%20V503%20P008%20CSR%20Synopsis.pdf](http://198.61.244.207/Upload/85_Applied Redaction V503 P008 CSR Synopsis.pdf) |
| 71 | V503-009 | GDS01C | NCT01304498 | <https://www.ncbi.nlm.nih.gov/pubmed/26090572> |
| 72 | V503-010 | 2013-001314-15 | NCT01984697 | <http://jamanetwork.com/journals/jama/article-abstract/2620075> |
| 73 | V503-020 | GDS07C | NCT02114385 | <http://www.sciencedirect.com/science/article/pii/S0264410X16304807> |
| 74 | V504-001 | 2007_566 | NCT00551187 | <https://www.ncbi.nlm.nih.gov/pmc/articles/PMC4514333/> |
| 75 | Not identified | Not identified | NCT00572832 | <https://www.ncbi.nlm.nih.gov/pubmed/20629576?dopt=Abstract> |
| 76 | Not identified | Not identified | NCT01489527 | <https://www.ncbi.nlm.nih.gov/pmc/articles/PMC4378717/> |
| **Identified journal publications of non-industry funded studies** | | | | |
| **No.** | **Study programme specific ID** | **Manufacturer specific ID** | **National Clinical Trial (NCT) ID** | **Publication source** |
| 77 | Not applicable | Not applicable | NCT00524745 | <http://jid.oxfordjournals.org/content/208/8/1325.long> |
| 78 | Not applicable | Not applicable | NCT00604175 | <https://www.ncbi.nlm.nih.gov/pmc/articles/PMC4305143/> |
| 79 | Not applicable | Not applicable | NCT00815282 | <http://ard.bmj.com/content/73/8/1500.long> |
| 80 | Not applicable | Not applicable | NCT00862810 | <http://jamanetwork.com/journals/jama/fullarticle/1682939> |
| 81 | Not applicable | Not applicable | NCT00923702 | <http://www.sciencedirect.com/science/article/pii/S1470204515004143> |
| 82 | Not applicable | Not applicable | NCT00949572 | <https://www.ncbi.nlm.nih.gov/pmc/articles/PMC3306286/> |
| 83 | Not applicable | Not applicable | NCT00956553 | <https://www.ncbi.nlm.nih.gov/pmc/articles/PMC3641072/> |
| 84 | Not applicable | Not applicable | NCT01173900 | <https://www.ncbi.nlm.nih.gov/pubmed/22711908> |
| 85 | Not applicable | Not applicable | NCT01386164 | <https://www.ncbi.nlm.nih.gov/pmc/articles/PMC4896591/> |
| 86 | Not applicable | Not applicable | NCT01717118 | <http://www.tandfonline.com/doi/full/10.1080/21645515.2015.1058458?scroll=top&needAccess=true> |
| 87 | Not applicable | Not applicable | NCT02276521 | <https://academic.oup.com/cid/article/doi/10.1093/cid/ciw865/2747464/Sustained-Antibody-Responses-6-Years-Following-1-2> |
| 88 | Not applicable | Not applicable | NCT02296255 | <http://www.tandfonline.com/doi/full/10.4161/hv.24337> |
| 89 | Not applicable | Not applicable | Not identified | <http://jnci.oxfordjournals.org/content/93/4/284.long> |
| 90 | Not applicable | Not applicable | Not identified | <http://online.liebertpub.com/doi/abs/10.1089/jwh.2009.1753> |
| 91 | Not applicable | Not applicable | Not identified | <http://www.tandfonline.com/doi/full/10.1080/21645515.2016.1277846?scroll=top&needAccess=true> |
| 92 | Not applicable | Not applicable | Not identified | <http://www.sciencedirect.com/science/article/pii/S0264410X13008128> |
