# Supplementary material for: Index of the human papillomavirus (HPV) vaccine industry clinical study programmes and non-industry funded studies: a necessary basis to address reporting bias in a systematic review
Source: Syst Rev. 2018 Jan 18;7:8. doi: 10.1186/s13643-018-0675-z (PMC5774129; doi:10.1186/s13643-018-0675-z)
Supplement: Supplementary file 5 — Data sharing agreement with GlaxoSmithKline.(PDF 301 kb) [file 13643_2018_675_MOESM5_ESM.pdf]

CSDR: The Sponsors have created the following standard contract template for clinical trial data sharing. This standard contract template was created in good faith to expedite the contracting process.

04/15/15 FINAL

DATA SHARING AGREEMENT

This DATA SHARING AGREEMENT (this "Agreement") is effective as of__________, ______ (the "Effective Date") between __________________ (the "Institution"), located at ________________________________ and ___________ ("Study Sponsor") located at________________________________ , regarding that certain **Research Proposal** submitted by __________________________________ and approved by the **Independent Review Panel**.

BACKGROUND

WHEREAS, Study Sponsor(s) and its Affiliates (as defined below) are engaged in the business of researching, developing manufacturing and marketing prescription pharmaceuticals and have accumulated certain data in clinical studies conducted by Study Sponsor(s) and its Affiliates; and

WHEREAS, Study Sponsor(s) wish to share data collected in clinical trials to support the free exchange of scientific information in accordance with standards found at https://clinicalstudydatarequest.com; and

WHEREAS, Institution desires to get access to and obtain a license to use certain data collected by Study Sponsor(s) accumulated in conduct of the clinical study/ies listed in Exhibit A (the "Clinical Studies") in order to conduct certain analyses; and

WHEREAS, Study Sponsor(s), on its behalf and on behalf of its Affiliates, is willing to grant such a license and access to these data via a secure, password protected analysis system subject to the terms and conditions set forth herein;

NOW, THEREFORE, in consideration of mutual promises, Study Sponsor(s) and Institution (each, a "Party" and collectively, the "Parties") agree as follows:

1. DEFINITIONS

1.1 "**Affiliate**" means any person or entity which, directly or indirectly, is controlled by, controls, or is under common control with a Study Sponsor

1.2 **"Access System"** is a single shared multi-sponsor environment that supports Researchers accessing the anonymized, patient-level clinical study data made available under this Agreement.

1.3 "**Analysis/Analyses**" refers to any and all analysis of the anonymized, patient-level data from the Clinical Studies, or any other source, as specifically described in the Research Proposal.

1.4 "**Analytical Tools**" includes, but is not limited to, any methodology, statistical methods, formulae or other methods or tools used in conducting the Analyses.

1.5 "**Independent Review Panel**" means the independent external review panel consisting of acknowledged experts which reviews the scientific merit of the request for data from the Clinical Studies, managed by the Wellcome Trust.

1.6 "**Lead Researcher**" means the researcher identified as the "Lead Researcher" in the Research Proposal or such other person that may be subsequently identified as a replacement Lead Researcher by the Institution in accordance with Section 2.4 of this Agreement.

1.7 "**New Intellectual Property**" means all data, discoveries, developments, inventions (whether patentable or not), improvements, methods of use or delivery, processes, know-how, or trade secrets which are made by a Researcher as a result of the conduct of Analyses or as a result of the use of any information provided to Institution or a Researcher by a Study Sponsor under this Agreement.

1.8 "**Research Proposa**l" means the research proposal as submitted by the Lead Researcher approved by the Independent Review Panel, including the plan for analyses of the anonymized, patient-level data from the Clinical Studies, or any other source, as described in detail in the research proposal, attached hereto as **Exhibit B**.

1.9 "**Researcher**" means each and any of the researchers listed in a Research Proposal or such other individuals that are employees, representatives and/or agents of Institution provided access to the data from the Clinical Studies.

1.10 "**Study Sponsor Confidential Information**" means all information (including, without limitation, anonymized patient-level data, research specifications or clinical trial/study protocols, reports, specifications, computer programs or models and related documentation, know-how, trade secrets, or business or research plans) of a Study Sponsor or any of its Affiliates that is provided to or otherwise made available to the Institution or a Researcher in connection with this Agreement.

1.11 "**Study Sponsor Uses**" means any and all uses of or related to any Study Sponsor data or a compound which is owned or controlled by a Study Sponsor on or after the Effective Date, including the compound(s) which was used to generate the patient-level data, which would otherwise be an infringement of any New Intellectual Property. For the avoidance of doubt, a related use includes, but is not limited to, a diagnostic test applicable to a disease treated by the compound or the class to which it belongs.

2. DATA SHARING

2.1 Study Sponsor hereby grants to Institution a one-time non-exclusive, non-transferable license for use by Researchers of the anonymized, patient-level data from the Clinical Studies for the sole purpose of conducting the Analyses according to the Research Proposal and for no other purpose. Study Sponsor(s) will provide the Researcher(s) with access to these data via the Access System.

2.2 Access to each Study Sponsor's data will be available to Institution for a twelve (12) month period. A Study Sponsor may extend Institution's access to its clinical data hereunder by written agreement of an authorized representative of such Study Sponsor.

2.3 Data provided by Study Sponsor(s) are Study Sponsor's(s') Confidential Information. This grant of a license shall not transfer any title or ownership rights in the Study Sponsor's(s') Confidential Information, including any intellectual property embodied therein, which title and ownership rights shall at all times remain with the Study Sponsor(s). The clinical trial data provided hereunder is provided 'AS IS' and Study Sponsor(s) makes no representations or warranties regarding the suitability of the data provided to Institution for Analysis.

2.4 Institution shall provide Study Sponsor(s) prior written notice of any replacement of the Lead Researcher and shall ensure that a replacement Lead Researcher acknowledges and agrees to the terms of this Agreement. A Researcher's access to and usage of the Access System will be subject to compliance by such Researcher with the access and usage conditions set forth on the Access System. Any Researcher may be denied access to the Access System and Institution shall be liable for any direct or indirect damages or liability arising from any non-compliance with the access and usage conditions. Study Sponsor(s) disclaim all liability to Institution or to any Researcher in connection with access or use of the Access System.

2.5 Institution shall not, and will ensure that Researchers shall not:

- download, save, edit, photograph, print, or transfer the whole or any portion of the Study Sponsor's(s') Confidential Information from the Access System for either the approved use or for any other purpose;
- remove, bypass, circumvent, neutralise or modify any technological protection measures of the Access System; or
- share any username, password or other account details with a third party or otherwise provide a third party with access to the Researcher's or Institution's account to the Access System.

2.6 Institution and Researchers shall comply with all applicable laws, regulations, codes, and guidelines, regarding handling, analysing and reporting analyses of clinical trial data, and all additional requirements set forth on Exhibit C identified by the Independent Review Panel which approved the Research Proposal.

2.7 Institution certifies that it is either non-profit or if not, a holder of a valid WHO-Drug Dictionary license with the Uppsala Monitoring Centre. This is a condition for the transfer of data sets containing WHO Drug Dictionary codes.

2.8 Institution shall inform the relevant Study Sponsor(s) immediately, no later than within twenty-four (24) hours, (and may also inform any regulatory authority) of any information concluded from the research conducted pursuant to the Research Proposal that in the Lead Researcher's judgment could impact the risk-benefit assessment of Study Sponsor's(s') product, including but not limited to, any safety concerns, identified as part of the Analysis. Sponsor(s) pharmacovigilance contact details are provided in Exhibit D. The relevant Study Sponsor(s) may take action regarding such information, including informing regulatory authorities or healthcare providers, or otherwise making such information public, even in advance of publication of the Analysis by Institution. Institution shall provide access and reasonable assistance to Study Sponsor(s) to utilize and implement any Analytical Tools for the sole purpose of reproducing the Analysis**.**

3. CONFIDENTIALITY

3.1 Institution shall ensure that Study Sponsor's(s') Confidential Information is kept confidential and is not used for any purposes other than the purpose(s) described in this Agreement. Neither Institution nor any of the

Researchers shall disclose Study Sponsor's(s') Confidential Information to third parties without the prior written approval of Study Sponsor(s) except as necessary for the purpose(s) described in this Agreement and under a written agreement by the third party (with the exception of regulatory authorities notified of Analysis results) to be bound by obligations at least as restrictive as those set forth in this Agreement in terms of maintaining the confidentiality of Study Sponsor Confidential Information. Institution shall safeguard Study Sponsor's(s') Confidential Information with the same standard of care that is used with Institution's most sensitive confidential information. At any time upon the request of relevant Study Sponsor, all tangible expressions, in any media, of Study Sponsor's(s') Confidential Information in Institution's or a Researcher's possession shall be delivered to the relevant Study Sponsor or, at the relevant Study Sponsor's option, destroyed.

3.2 The obligations of confidentiality and limited use under this Section shall not extend to any information: (i) which is or becomes publicly available, except through breach of this Agreement (ii) which Institution or a Researcher can demonstrate that it possessed free of any obligation of confidence prior to, or developed independently from, disclosure under this Agreement; (iii) which a Researcher or Institution receives from a third party which is not legally prohibited from disclosing such information; or (iv) which Institution or a Researcher is required by law to disclose, provided that the other party is notified of any such requirement with sufficient time to seek a protective order or other modifications to the requirement.

3.3 The obligations of this Section shall survive this Agreement for a period of fifteen (15) years after the Effective Date.

4. INTELLECTUAL PROPERTY

4.1 Study Sponsor's(s') Confidential Information and all tangible expressions, in any media, of Study Sponsor's(s') Confidential Information are the sole property of the Study Sponsor.

4.2 All New Intellectual Property shall be the sole property of Institution; however, Institution will notify each Study Sponsor, promptly and in writing, of any New Intellectual Property. Institution hereby grants to each Study Sponsor a perpetual, non-exclusive, fully-paid up, royalty-free, irrevocable, worldwide, unrestricted license under any New Intellectual Property for Study Sponsor Uses, with the right to sublicense through multiple tiers. Institution further grants an exclusive option, to be exercised within one hundred eighty (180) days from notice of the New Intellectual Property to negotiate in good faith an exclusive, fee-bearing, worldwide license with the right to sublicense through multiple tiers to any New Intellectual Property which Institution may have or obtain. If additional assistance from the Institution is requested beyond the rights provided by the non-exclusive license, Institution will provide reasonable assistance to each Study Sponsor, upon commercially reasonable terms that are at least as favorable to the Study Sponsor as the terms agreed with any other licensee for such assistance, to facilitate Study Sponsor in fully utilizing any New Intellectual Property.

4.3 If a Study Sponsor exercises its option to negotiate an exclusive license, Study Sponsor and Institution will exclusively negotiate in good faith, for up to one hundred eighty (180) days or such mutually agreeable longer period, regarding commercially reasonable terms for an exclusive, worldwide, fee-bearing license, including the right to sublicense, for the Study Sponsor and its Affiliates to make, have made, use, sell or otherwise dispose of the subject matter of the New Intellectual Property or products incorporating the subject matter of the New Intellectual Property subject to any non-exclusive licenses granted in section 4.2. In the event that the Study Sponsor does not exercise its option to negotiate an exclusive license, or in the event Institution and Study Sponsor fail to agree to commercially reasonable exclusive license terms following good faith negotiation, Institution may negotiate further non-exclusive license terms with third parties. Any such terms shall be consistent with the non-exclusive license granted to Study Sponsor in Section 4.2. Should any terms be agreed with a third party in accordance with this section, then for five (5) years after the Effective Date, Institution will notify Study Sponsor(s), within thirty (30) days of the effective date of any such agreement, of the identity of the third party.

4.4 Institution agrees to obtain written agreements with all Researchers which assign, without additional consideration, all rights, title and interests in New Intellectual Property to Institution for subsequent licensing to Study Sponsor(s).

4.5 Study Sponsor shall have no further obligations resulting from the assignment and/or exploitation of any New Intellectual Property.

4.6 The obligations of this Section shall survive termination of this Agreement.

5. PUBLICATION

5.1 Institution agrees to post a summary of the Analysis Plan on a publicly available internet register or website prior to conducting the Analysis. Institution agrees to post summary results of the Analysis on the same publicly available internet register or website within one (1) year of completing the Analysis and, thereafter, to permit Study Sponsor(s) to post a summary of the Analysis results on any publicly available internet register maintained by Study Sponsor(s).

5.2 Institution also agrees to submit the results of the Analysis for publication in peer-reviewed literature, in a manner consistent with the publication plan set forth in the Research Proposal (a "Publication"), with such Publication appropriately including citations or register identification numbers for the studies used in the analysis and disclosing the strengths and weaknesses of the Analysis methodology. Institution may use Study Sponsor’s name solely for that purpose.

5.3 Institution shall ensure compliance with the additional provisions regarding publication of the results of the Analyses set forth in Exhibit E.

5.4 Additionally, Institution shall provide Study Sponsor(s) a reference citation upon publication. Institution agrees, following publication, to provide other researchers with additional details of the Analysis on request and to provide access and reasonable assistance to those other researchers to utilize and

implement any Analytical Tools for the sole purpose of reproducing the Analysis, subject to adequate provisions to protect Study Sponsor's Confidential Information (see Section CONFIDENTIALITY).

5.5 The obligations of this Section 5 shall survive termination of this Agreement.

6. INDEPENDENT CONTRACTOR

The relationship of the parties is that of independent contractors. Neither party is the partner, joint venturer, or agent of the other and neither party has authority to make any statement, representation, commitment, or action of any kind which purports to bind the other without the other's prior written authorization.

7. ASSIGNMENT

A Study Sponsor may assign its rights and duties under this Agreement without Institution's consent. Any assignment of any rights or obligations under this Agreement by Institution is valid only upon the prior written consent of Study Sponsor(s). To the extent permitted above, this Agreement shall be binding upon and inure to the benefit of the parties and their permitted successors and assigns.

8. REPRESENTATIONS AND WARRANTIES

8.1 Institution represents and warrants that it does not have, and will not enter into, any legal or contractual obligations that would prevent it from complying with its obligations under this Agreement, including without limitation, the obligations of Section 4, without the written approval of Study Sponsor.

8.2 Institution shall be responsible for the compliance of the Lead Researcher and any other Researcher to the terms of this Agreement.

8.3 Lead Researcher will obtain any regulatory or ethics approvals necessary to conduct the Research Proposal.

9. DATA PROTECTION

Institution acknowledges the importance of data privacy of individuals to whom accessed data may relate, and commits to comply with all applicable federal, state and local laws and regulations relating to data protection and the privacy of subject health information, not to attempt to identify subjects, and not to combine accessed data with other sources of data that would lead to the identification of any individual.

10. TERM AND TERMINATION

10.1 The term of this Agreement shall commence on the Effective Date and continue through completion of the Research Proposal. Certain provisions of this Agreement survive termination of this Agreement as expressly set forth herein.

10.2 Institution's or a Researcher's use of any of Study Sponsor's(s') Confidential Information or Intellectual Property in violation of any law or of any terms or limitations imposed by this Agreement shall be a violation of this Agreement and Study Sponsor may immediately terminate the rights granted under this Agreement unless earlier terminated as set forth below.

10.3 Study Sponsor(s) may, in addition to any other rights and remedies available to Study Sponsor(s), terminate this Agreement by giving Institution written notice of such termination in the event Institution or any Researcher materially breaches any of the terms and conditions of this Agreement and fails to cure such breach or default as promptly as practicable and, in any event, not more than thirty (30) days after respective Study Sponsor gives Institution written notice specifying the details thereof. Notwithstanding the foregoing, in the event that any breach is not reasonably capable of cure within the thirty (30) day period, it shall be sufficient if Institution promptly commences appropriate action to cure and diligently pursues such action until complete.

11. GOVERNING LAW; VENUE

This Agreement shall be interpreted and governed exclusively by the laws of the country and, as applicable, the government subdivision, in which the Study Sponsor is located (as set forth at the beginning of this Agreement) without reference to its rules of conflict of law. Any dispute arising under or in connection with this Agreement shall be subject to the exclusive jurisdiction of the competent courts of that country or government subdivision.

12. ENTIRE AGREEMENT

This Agreement represents the entire and integrated agreement between the parties and supersedes all prior negotiations, representations or agreements, either written or oral, regarding its subject matter.

13. COUNTERPARTS

This Agreement may be executed in two or more counterparts, each of which shall be considered an original instrument. Each counterpart will be considered a valid and binding original, and all counterparts taken together shall constitute one and the same agreement. Faxed copies and scanned copies of original signatures shall be deemed as effective as original signatures. Once signed, any reproduction of this Agreement made by reliable means (e.g., photocopy, facsimile, scan) is considered an original.

IN WITNESS WHEREOF, the parties have executed this Agreement by their duly authorized officers or representatives on the date set forth below, effective as of the Effective Date.

**Study Sponsor** [●]	**Institution**
By: ______________________	By: ______________________
Name:______________________	Name:______________________
Title:______________________	Title:______________________
Date:______________________	Date: ______________________

CSDR: The Sponsors have created the following standard contract template for clinical trial data sharing. This standard contract template was created in good faith to expedite the contracting process.

Acknowledged and Agreed to by the Lead Researcher:

By: ______________________________________

Name:_______________________________

Title:________________________________

Institution: ___________________________

Date:________________________________

Attachments:
Exhibit A – Clinical Studies Listing
Exhibit B – Research Proposal–
Exhibit C – Independent Review Panel Requirements
Exhibit D – Study Sponsor PV Contact Information
Exhibit E – Additional Study Sponsor Publication Provisions

Exhibit A

Clinical Studies List

Exhibit B
Research Proposal

Exhibit C

Independent Review Panel Requirements

CSDR: The Sponsors have created the following standard contract template for clinical trial data sharing. This standard contract template was created in good faith to expedite the contracting process.

Exhibit D

Study Sponsor Pharmacovigilance Contact Information

CSDR: The Sponsors have created the following standard contract template for clinical trial data sharing. This standard contract template was created in good faith to expedite the contracting process.

Exhibit E

Additional Study Sponsor Publication Provisions

[This Exhibit may include the following, in whole or in part, in respect of the obligations of Institution/Lead Researcher to any particular Study Sponsor(s) regarding publication of the Analyses. Note that the applicability of the following obligations may vary among one or more Study Sponsors and any such variance would be reflected in this Exhibit. For example, not all Study Sponsors will require a thirty-day prior review of a publication of the Analyses.]

Publication Terms:

Lead Researcher shall submit to Study Sponsor(s) (i) a copy of the summary results of the Analysis at the time of posting the summary results as well as (ii) a copy of any proposed Publication at least thirty (30) days prior to submission to a scientific congress or journal to give Study Sponsor the opportunity for input regarding medical and scientific accuracy, supplementary scientific information, to object to any inclusion of Study Sponsor's(s') Confidential Information and to review for patentable subject matter. Lead Researcher is not required to accept Study Sponsor's comments regarding medical and scientific accuracy and supplementary scientific information. Lead Researcher shall submit to Study Sponsor(s) a copy of any proposed Publication within five (5) days after submission to a scientific congress or journal.
